# Functional brain biomarkers of self-referential bias in remitted depressed outpatients: a randomized controlled trial

**DOI:** 10.1016/j.nicl.2026.104025

**Published:** 2026-06-19

**Authors:** Liliana C. Wu, Jordan L. Livingston, Zindel V. Segal, Norman A.S. Farb

**Affiliations:** a Department of Psychology, University of Toronto Mississauga, 3359 Mississauga Road, Mississauga, ON L5L 1C6, Canada; b Graduate Department of Psychological Clinical Science, University of Toronto Scarborough, 1265 Military Trail, Toronto, ON M1C 1A4, Canada

**Keywords:** Depression, fMRI, Self-reference, Relapse, Sensory deactivation, Negative self

## Abstract

**Background:**

Negative self-referential bias (NSB) is a hallmark of depression, yet its neural signature and link to relapse vulnerability remain unclear. We investigated whether NSB and its associated neural activity predict relapse and depressive symptoms following prophylactic psychotherapy.

**Methods:**

This is a two-year prospective neuroimaging study nested within a randomized controlled trial of Mindfulness-Based Cognitive Therapy and Cognitive Behavior Therapy with a Well-Being focus. Remitted depressed outpatients (*N* = 81) completed the Self-Referential Encoding Task during fMRI pre- and post-treatment and were followed bi-monthly. Study preregistration: https://osf.io/s4n8j.

**Results:**

NSB predicted higher depressive symptoms (b = 0.10; 95% CI, 0.06 to 0.14; *p* ≤0.001) but not relapse. Greater NSB neural activity within frontal DMN and SN was associated with relapse status (DMN: b = 0.32; 95% CI, 0.09 to 0.55; SN: b = 0.36, 95% CI, 0.09 to 0.63), whereas DMN–SN connectivity was not. Static whole brain relapse biomarkers included elevated prefrontal activation and somatosensory deactivation. In non-relapsers, treatment-related attenuation of somatosensory deactivation predicted lower risk. Subgenual reactivity was the best prefrontal relapse indicator (Hazard Ratio = 1.84, 95% CI, 1.23 to 2.77), but somatosensory deactivation was the best overall relapse indicator (Hazard Ratio = 0.16; 95% CI, 0.05 to 0.52).

**Conclusions:**

Relapse vulnerability reflects not only heightened prefrontal negative self-processing but also insufficient recruitment of somatosensory systems supporting embodied self-experience, providing a more comprehensive model of depression relapse vulnerability.

**Trial registration:**

ClinicalTrials.gov Identifier: NCT01178424

## Highlights

• Prospective study of neural biomarkers of negative self-reference and relapse.
• Relapse was associated with prefrontal activation and somatosensory deactivation.
• Reduced somatosensory deactivation predicted lower relapse risk in non-relapsers.
• Targeting embodied awareness and sensory integration may optimize treatment.

## 1 Introduction

Major Depressive Disorder (MDD) is among the most prevalent psychiatric conditions (Kessler et al., 2012) and a leading cause of disability worldwide ([Bibr bb0085][Bibr bb0180]). MDD is highly recurrent; over half of individuals diagnosed with MDD experience multiple episodes, developing a more chronic course of illness (Burcusa and Iacono, 2007; Eaton et al., 2008; Monroe and Harkness, 2011).

Given its recurrent nature, elucidating the cognitive and neural mechanisms underlying depression vulnerability is critical. Vulnerability has long been linked to negative self-referential bias (NSB), whereby individuals engage in self-criticism, preferentially endorsing negative over positive traits (Brockmeyer et al., 2015; LeMoult et al., 2017; Pyszczynski et al., 1987; Watkins and Teasdale, 2004) —a bias associated with greater symptom severity (Bradley and Mathews, 1983; Gotlib et al., 2004; Timbremont and Braet, 2004).

At a neural level, the dorsal nexus hypothesis (Sheline et al., 2010) posits that excessive self-criticism and rumination in depression stem from resting-state hyperconnectivity of dorsal medial prefrontal cortex with three large-scale brain networks: the Default Mode Network (DMN), the Central Executive Network (CEN), and the Salience Network (SN). Critically, frontal DMN regions such as dmPFC support evaluative self-referential processing, the appraisal of self-relevant information for personal meaning and affective significance, which is functionally distinct from the autobiographical self-reference associated with posterior DMN regions such as precuneus(Buckner and Carroll, 2007; Lemogne et al., 2012). In parallel, SN regions including the anterior cingulate cortex contribute to assigning personal meaning to negative information and sustaining attention toward affectively relevant content (Burklund et al., 2014; Disner et al., 2011; Jiang, 2024). Frontal DMN and SN regions therefore represent the most theoretically motivated targets for paradigms probing self-critical evaluation.

Empirical evidence confirms prefrontal hyperconnectivity in active depression (Bertocci et al., 2023; Chou et al., 2023; Lemogne et al., 2010; Sabbah and Northoff, 2025; Sheline et al., 2009) and recurrent episodes (Bertocci et al., 2023; Dunlop et al., 2023; Hamilton et al., 2012). In addition to connectivity abnormalities, prior work has implicated altered task-related activation in frontal regions including medial prefrontal cortex, DLPFC, and anterior cingulate cortex during self-referential processing in depression, suggesting these regions also support dysphoric self-focus during task performance (Li et al., 2017; Lou et al., 2019; Nejad et al., 2013). While the dorsal nexus account implicates all three networks, the present study focused primarily on frontal DMN and SN regions given their established roles in self-referential evaluation and affective salience. CEN involvement, suggested by these broader accounts, is also considered in exploratory whole-brain findings. The extent to which abnormalities across these systems support dysphoric self-referential bias that promotes episode return remains unclear.

Yet vulnerability may stem from more than aberrant prefrontal processing alone. Emerging research implicates abnormal sensorimotor activity as an additional candidate vulnerability factor (Farb et al., 2011, Farb et al., 2022; Ray et al., 2021; Seth and Friston, 2016). Sensorimotor systems may support a more embodied representation of present-moment experience (Farb et al., 2007). Consistent with this view, prior analysis from the current cohort implicated both interoceptive and sensory deactivation, including the somatosensory cortex, insula, and visual cortex, with future relapse vulnerability (Farb et al., 2022). Such findings extend existing accounts that have linked prefrontal hyperactivity with relapse risk (Lemogne et al., 2012; Sheline et al., 2009) to include a profile of sensory hypoactivity. Related functional connectivity findings have likewise highlighted sensorimotor dysfunction as a potential predictor of MDD recurrence (Ray et al., 2021). Consistent with this framework, greater awareness of present-moment experience has been associated with less negatively biased self-processing (Ford et al., 2023). Therefore, relapse vulnerability may be marked not only by exaggerated prefrontal recruitment to negative self-relevant information, but also by reduced engagement of embodied processing systems that could otherwise buffer against habitual patterns of NSB marked by self-criticism.

This framework also has implications for interventions designed to prevent relapse. It remains unclear how prophylactic psychotherapy impacts candidate biomarkers of dysphoric bias, which is important for determining whether such markers reflect static vulnerability factors, modifiable treatment targets, or both following symptom remission.

The present study integrated indicators of NSB, prefrontal and sensorimotor processing, and the impact of prophylactic intervention in a single prospective study of MDD vulnerability. We operationalized NSB via patterns of both behavioral self-endorsement and neural (fMRI) activity—searching for indicators of future depression vulnerability in remitted individuals undergoing prophylactic psychotherapy.

We hypothesized that greater NSB would predict future relapse (primary hypothesis), higher depressive symptoms, and lower decentering, a measure of psychological distancing (secondary hypothesis). We also hypothesized that relapse would be predicted by dysphoric self-referential brain activity, indexed by greater activation for negative than positive traits within frontal regions of the DMN and SN, as well as by stronger functional connectivity between these regions (primary hypothesis). Finally, based on our previous findings in a mood induction task (Farb et al., 2022), we conducted an exploratory analysis that was not part of our preregistration. Specifically, we examined treatment-related changes in somatosensory responses during self-referential processing to assess whether these changes might serve as candidate marker of relapse vulnerability.

We conducted a prospective neuroimaging trial of prophylactic psychotherapy aimed at reducing depression relapse vulnerability. Participants underwent fMRI scanning before and after the intervention and were followed for two years. Self-referential processing was assessed using the Self-Referential Encoding Task (SRET**)** a validated paradigm (Derry and Kuiper, 1981) for measuring trait endorsement patterns and corresponding neural activations (see trial protocol in Supplement 1).

## 2 Methods

Clinical and psychometric data of this trial are reported in detail elsewhere (Farb et al., 2018; Segal et al., 2019) and in the Methods (Supplement 2). Briefly, fully remitted participants were randomized to receive either Mindfulness Based Cognitive Therapy (MBCT) (Segal et al., 2012) or Cognitive Behavior Therapy with a Well-Being focus (WB-CT) (Fava, 2016) before entering a 2-year follow-up period. Participants completed behavioral and fMRI assessments both before and after treatment. All provided informed consent under a protocol approved by the institutional review board at the Centre for Addiction and Mental Health (CAMH), and the study was pre-registered on the Open Science Framework (https://osf.io/s4n8j) before data inspection.

### 2.1 Participants

Eligibility criteria required that remitted depressed participants (1) did not currently meet a diagnosis of Major Depressive Disorder (MDD) according to DSM-IV criteria, (2) scored ≤12 on the Hamilton Depression Rating Scale (HDRS-17), (3) had ≥1 previous episode of MDD, (4) were between 18 and 65 years of age, (5) English speaking, and could provide informed consent. Excluded participants were (1) confirmed of a current diagnosis of Bipolar Disorder, Substance Abuse Disorder, Schizophrenia, or Borderline Personality Disorder, and (2) currently receiving psychotherapy or practicing meditation > once per week or yoga > twice per week.

Of 155 participants in the parent trial, 99 participants enrolled in the neuroimaging study. The neuroimaging subsample did not differ on demographic or clinical history variables compared to the parent trial sample at pre- or post-intervention scan time points.(Farb et al., 2018; Segal et al., 2019) Sample size justification, attrition, and retention rate over the two-year follow-up are reported elsewhere(Farb et al., 2022) and detailed in the Methods (Supplement 2). A final sample of 81 participants completed both baseline and post-intervention scans and showed adequate behavioral task variance. CONSORT flow information is presented in Fig. S1.

### 2.2 Randomization, masking, and intervention

Eligible patients were randomized in blocks of four, using computer generated quasi-random numbers, to receive eight weekly group sessions of either MBCT or WB-CT. Randomization was performed by the study coordinator via a randomization table; investigators were blind to group allocation during the intervention and follow-up assessment periods. Details of the prophylactic psychotherapy interventions are reported elsewhere (Farb et al., 2018) and described in the Methods (Supplement 2).

### 2.3 Assessment and outcomes

The primary outcome was time to relapse, defined as meeting DSM-IV criteria for a major depressive episode. Following treatment, participants entered a 2-year follow-up period during which relapse status was monitored every two months confirmed using SCID and HDRS-17 scores ≥16. Residual symptoms, lingering depressive symptoms after clinical remission, and decentering, reflecting psychological distance and perspective-taking (Segal et al., 2019), were also examined longitudinally across the follow-up interval.

#### 2.3.1 Functional magnetic resonance imaging task

Participants completed the SRET during fMRI at baseline and post-treatment, consisting of two 80-trial runs of the self-referential bias task, interleaved with a dysphoric mood induction task (Farb, 2010; Farb et al., 2011, Farb et al., 2022). On each trial, participants viewed a cue (self, other, or case) paired with a trait word (positive or negative) and made a yes/no response for endorsement. In the self condition, participants judged whether the adjective described themselves; in the other condition, they judged whether the adjective described another person; and in the case condition, they made a non-self-referential perceptual judgment about the word (i.e., whether the word was presented in upper- or lowercase letters). Thus, the self and other conditions indexed evaluative processing, whereas the case condition served as a non-evaluative comparison. Unlike our prior report in this cohort, which focused on neural responses related to dysphoric mood induction, the present study focuses on self-referential encoding and its relation to relapse vulnerability. The present analyses focused on behavioral and neural responses during the SRET conditions. Each fMRI session included two blocks of 48 task trials and 32 fixation trials. fMRI acquisition and preprocessing parameters were performed using a standardized pipeline (see Methods in Supplement 2 for full details). Briefly, functional images were spatially smoothed with an 8 mm FWHM Gaussian kernel, and first-level SPM models applied a 180 s high-pass filter and included motion regressors, CSF signal, framewise displacement, and aCompCor components as nuisance covariates.

SRET endorsement patterns were analyzed to derive participant-level positive and negative self-endorsement rates. Endorsement rates for positive and negative words were separately calculated as the proportion of “yes” responses across all valid trials of the corresponding valence. NSB was quantified as the behavioral index ratio of negative to positive endorsement rates at baseline and post-intervention. To mitigate the influence of outliers, self-referential bias scores were winsorized at the 5th and 95th percentiles.

### 2.4 Statistical analysis

#### 2.4.1 Positive and negative endorsement neural activity

Whole-brain, voxel wise analyses were conducted on participants (*N* = 81) who completed both fMRI scanning at pre- and post-intervention with adequate behavioral task variance. At the first level, event-related general linear models in SPM12 modeled the self, other, and case conditions, each crossed with positive and negative valence. Standard motion parameters and global CSF signals were included as nuisance regressors (Birn, 2012; Kong et al., 2012). Contrast images for each Condition × Valence combination were generated for each participant and session.

At the second level, these contrasts entered a mixed factorial model with within-subject factors of time (baseline, post-intervention), condition (self, other, case)**,** and valence (positive, negative). Positive and negative endorsement rates were included as between-subject covariates to account for individual differences in endorsement bias. Exploratory conjunction analyses tested two opposing endorsement-related neural response profiles: (1) self-criticism: regions associated with decreases in positive endorsement and increases in negative endorsement, and (2) self-affirmation: regions associated with increases in positive endorsement and decreases in negative endorsement. Conjunction analyses were performed by thresholding each component contrast at p = √0.005, yielding a conjoint significance level of *p* = .005, with cluster extent k = 400. Modelling self-criticism and self-affirmation in separate covariate models (as opposed to a single model with both covariates) did not meaningfully alter the observed response pattern.

#### 2.4.2 Associations of self-referential bias and depression vulnerability

Survival analyses were conducted to investigate whether NSB predicted relapse risk. Mixed-effects models examined associations between NSB, residual symptoms and decentering, adjusting for age, gender, past depressive episodes, and antidepressant medication (ADM). Additional linear mixed-effects models assessed whether baseline, and post-treatment NSB predicted residual symptoms and decentering over the follow-up period, controlling for the prior timepoint value, time, past episodes, age, and ADM. Variance Inflation Factor values for all covariates were below 5 and above 0.2, indicating no multicollinearity concerns (Table S1, Supplement 2). All analyses were conducted in R version 4.3.1 (R Core Team, 2023).

#### 2.4.3 Dysphoric self-referential activity and connectivity within frontal DMN and SN and relapse vulnerability

Dysphoric self-referential activity was quantified using region of interest (ROI)-based extraction from a priori anatomical masks derived from the Harvard–Oxford cortical atlas. The frontal DMN was defined as a dorsomedial prefrontal cortex (dmPFC) mask created by combining the superior frontal gyrus and paracingulate gyrus. The frontal SN ROI was defined as the anterior cingulate cortex (ACC), using the anterior portion of the cingulate cortex from the Havard-Oxford atlas. For each participant and session, contrast images for Self-Negative and Self-Positive conditions were extracted and median activation values were computed within each ROI. The median was used as a robust summary measure to reduce the influence of extreme voxel values within the mask; this approach was preregistered (Supplement 1). Dysphoric self-referential activity was indexed as the Self-Negative minus Self-Positive difference within each ROI, yielding separate frontal DMN and frontal SN metrics.

Functional connectivity between frontal DMN and SN was estimated using ROI-based correlations of denoised voxelwise BOLD time-series. For each participant and session, voxelwise time series were extracted from the anatomically defined dmPFC and ACC masks across the full task runs, mean-centered, band-pass filtered, and denoised by standard motion parameters and global CSF signal (Birn, 2012; Kong et al., 2012). Between-network and within-network connectivity were quantified as the mean Fisher z–transformed correlation between all frontal DMN and SN voxels.

Dysphoric self-referential activity and connectivity measures were entered into mixed-effects models comparing relapsers and non-relapsers, controlling for ADM, past episodes, residual symptoms, and their interaction with time. Cox proportional hazards models further tested whether frontal DMN and SN dysphoric self-referential activity predicted relapse, adjusting for baseline and post-treatment residual symptoms, ADM, and past episodes. Preregistration deviations are detailed in the Methods (Supplement 2). Briefly, preregistration deviations included replacing the endorsed-only contrast with the Self-Negative > Self-Positive contrast due to insufficient endorsed trials, using a priori anatomical ROIs rather than participant-specific functional-anatomical intersections, adopting the Self > Case contrast for later whole-brain analyses following sensitivity analyses, and not entering interoceptive dysfunction variables separately because they loaded onto the residual symptoms factor.

#### 2.4.4 Static and dynamic neural markers of relapse vulnerability during self-referential processing

Whole-brain static and dynamic neural markers were derived from the Self > Case contrast. First-level models estimated self, other, and case conditions collapsed across valence, and resulting contrasts were entered into second-level analyses to distinguish static vulnerability markers from dynamic treatment-related changes.

**Fig. 1:**
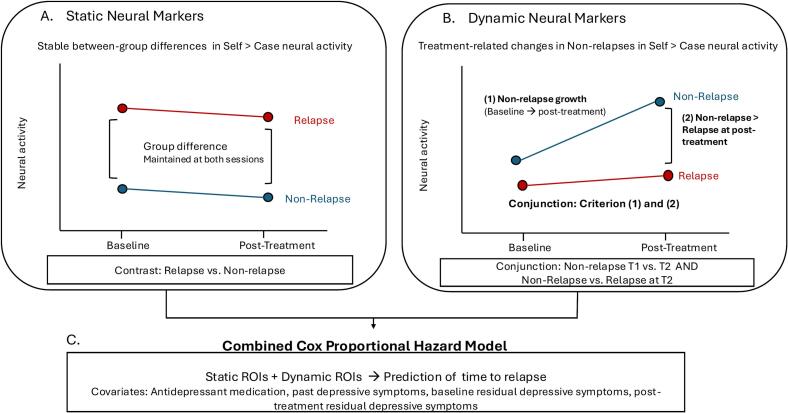
Schematic overview of the derivation of static and dynamic neural markers of relapse vulnerability. Whole brain markers were derived from the Self>Case contrasts at baseline and post-treatment. Panel A illustrates static markers, defined as stable between-group differences in Self > Case activity between relapsers and non-relapsers across baseline and post-treatment. Panel B illustrates dynamic markers, defined as treatment-related changes associated with non-relapse, identified from the conjunction of non-relapse growth and greater post-treatment activation in non-relapsers compared to relapsers. Panel C shows the combined Cox proportional hazards model composed of extracted static and dynamic ROI values predicting time to relapse, adjusting for antidepressant medication, past episodes, and baseline and post-treatment residual symptoms. Activation values are schematic and shown for illustration only.

Static markers were identified from the Relapse > Non-Relapse and Non-Relapse > Relapse contrasts within a 2 (Time: baseline, post-intervention) × 2 (Relapse status: Relapse, Non-relapse) × 2 (Treatment: MBCT, WB-CT) mixed factorial model. Thus, “static” refers to stable between-group differences in self-referential processing between relapsers and non-relapsers across assessment timepoints. Dynamic markers were identified using a conjunction analysis isolating regions showing consistent increases among non-relapsers across both time points (non-relapse growth) and regions where increases were greater in non-relapsers than relapsers at post-intervention session. Thus, “dynamic” refers to treatment-related changes in neural activity associated with non-relapse. Whole-brain results were thresholded at *p* < .005, cluster extent *k* = 400, and FDR corrected at *p* < .05. A schematic overview of the derivation of static and dynamic neural markers is shown in Fig. 1.

Parameter estimates from all significant ROIs were entered into mixed-effects models comparing relapsers and non-relapsers, while controlling for ADM, past episodes, residual symptoms, and their interaction with time. To identify optimal neural thresholds for relapse prediction and treatment response, receiver operating characteristic curve analyses were conducted using the *cutpointr* package(Thiele and Hirschfeld, 2021). Cox proportional hazards models tested whether static and dynamic markers, separately and jointly, predicted relapse, adjusting for baseline and post-treatment residual symptoms, ADM, and past episodes.

## 3 Results

Participants who relapsed reported higher residual symptoms, and fewer days well than non-relapsers. No other demographic or clinical history differences were observed (Table 1).

**Table 1:** Clinical and demographic characteristics by remitted depressed outpatients relapse status at two-year follow-up.

a*Demographics*	*Relapse (n = 27)*	*Non-relapse (n = 54)*	T-value	Chi-square	*P*-value
Intervention, n(%) MBCT	14(51.9%)	30(55.6%)	–	0.006	0.94

Age, mean (SD)	36.81(10.76)	40.20(12.80)	1.25	–	0.215

Gender, n (%) Female	21(77.7%)	33(61.1%)	–	1.56	0.21

Ethnicity, n(%)			–	1.82	0.61
Caucasian	24(88.9%)	44(84.6%)	–	–	–
Afro-Canadian	0	0	–	–	–
Asian/East-Asian	5(9.6%)	1(3.7%)	–	–	–
Hispanic	1(1.9%)	0	–	–	–
Other	2(3.8%)	2(7.4%)	–	–	–

Education, n(%)			–	2.95	0.23
High school	2(7.4%)	11(20.8%)	–	–	–
College/University	21(77.8%)	32(60.4%)	–	–	–
Graduate school	4(14.8%)	10(18.9%)	–	–	–

Employment, n(%)					
Full time job	15(60.0%)	29(59.2%)	–	2.98	0.39
Part time job	4(16.0%)	9(18.4%)	–	–	–
Unemployed	6(24.0%)	7(14.3%)	–	–	–
Student/other	0	4(8.2%)	–	–	–

Age of onset of first episode of depression, mean (SD)	20.56(10.65)	20.55(9.17)	−0.003	–	0.99

Number of past depression episodes, mean (SD)	4.52(2.68)	3.85(2.53)	−1.08	–	0.29

Previous hospitalization, n(%)	6(22.2%)	11(21.2%)	–	0.00	1

Suicide attempts, n(%)	3(11.1%)	6(11.3%)	–	0.00	1

Family Hx depression, n(%)	18(69.2%)	37(71.2%)	–	0.53	0.77
Antidepression at intake, n(%)	19(70.4%)	33(61.1%)	–	0.33	0.57

Previous or current psychotherapy, n(%)	22(81.5%)	43(82.7%)	–	0.53	0.77

Remission achieved via, n(%)			–	3.63	0.46
CBT	5(21.7%)	11(24.4%)	–	–	–
Psychotherapy	4(17.4%)	2(4.4%)	–	–	–
Medication	6(26.1%)	14(31.1%)	–	–	–
Medication & psychotherapy	6(26.1%)	11(24.4%)	–	–	–
Other	2(8.7%)	7(15.6%)	–	–	–

Number of treatment sessions attended, mean (SD)	6.74(1.87)	6.70(1.31)	0.106	–	0.92

bResidual Symptoms, mean (SD)	0.29(0.83)	−0.22(0.90)	−2.51	–	**0.015**

bDecentering, mean (SD)	−0.39(0.93)	−0.26(1.27)	0.53	–	0.60

cDays well, mean (SD)	307.11(236.50)	572.28(244.06)	4.71	–	**0.00002**
dPositive endorsement rate	0.87(0.13)	0.82(0.18)	−0.1.46	–	0.148
dNegative endorsement rate	0.25(0.20)	0.19(0.21)	−1.30	–	0.198
eNegative self-referential bias	0.31(0.27)	0.29(0.50)	−0.21	–	0.831

a Participants had the option to disclose demographic information at their discretion; as such, the total number of demographic variable responses may be less than the full sample size. Percentages are relative to the total number of respondents for each demographic variable. Significant group differences are shown in bold.

b Residual symptoms and Decentering were defined using the composite score described in Segal et al., 2019.

c Days well indicates the number of days without relapse.

d Positive endorsement rate was defined as the proportion of positive words endorsed in the self condition. Negative endorsement rate was defined as the proportion of negative words endorsed in the self condition.

e Negative self-referential bias was calculated as the ratio of negative to positive endorsement rates,

### 3.1 Self-referential patterns and depression vulnerability

**Fig. 2:**
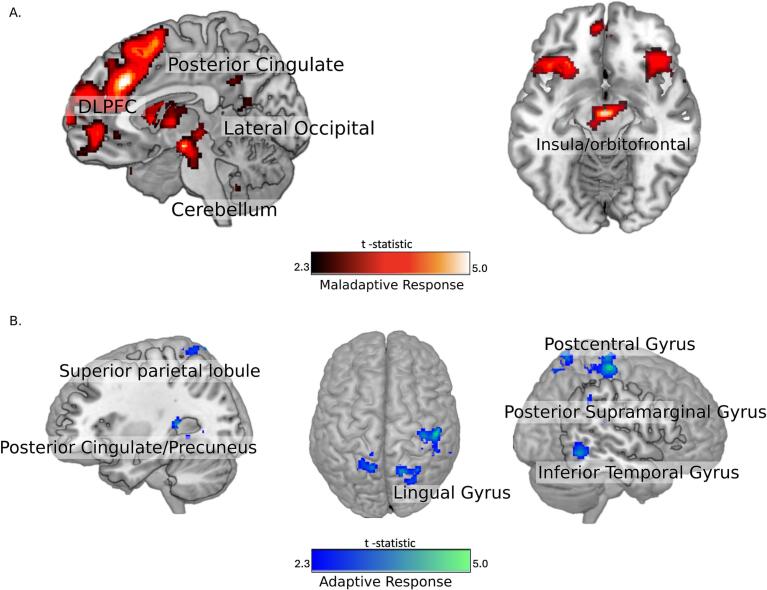
Neural activity correlates of self-criticism and self-affirmation. Panel A shows self-critical activity (red), identified as the conjunction of [activation associated with negative trait endorsement] and [deactivation associated positive trait endorsement]. Conjunction maps were thresholded by applying a voxelwise threshold of *p* < .071 to each component contrast, yielding a conjoint significance level of *p* < .005, with cluster extent *k* ≥ 400 voxels. Significant activations were observed in the orbitofrontal/insula region, cerebellum, left dorsolateral prefrontal cortex, left posterior cingulate gyrus and left lateral occipital cortex. Panel B shows self-affirming activity (blue), identified as the conjunction of [activation associated with positive trait endorsement] and [deactivation associated with negative negative-trait endorsement], using the same conjoint threshold. Significant activations were observed in the right postcentral gyrus, right inferior temporal gyrus, right lingual gyrus, right posterior supramarginal gyrus, left posterior cingulate/precuneus, and left superior parietal lobule. (For interpretation of the references to color in this figure legend, the reader is referred to the web version of this article.)

We first characterized behavioral and whole-brain neural correlates of self-referential bias at the task level. Fig. 2. shows neural activity correlates of self-criticism (Panel A) and self-affirmation (Panel B). Self-criticism was associated with higher activations in a broader set of prefrontal and posterior regions, including orbitofrontal/insula, cerebellum, left dorsolateral prefrontal cortex, left posterior cingulate gyrus, and left lateral occipital cortex, whereas self-affirming responses were linked to posterior somatosensory regions (Table S2, Supplement 2). Higher residual symptoms were positively associated NSB, while both Time and Decentering were negatively associated with NSB (Table S3 and Fig. S2, Supplement 2).

Greater NSB at post-treatment also predicted greater residual symptoms across the 4–24 month follow-up (Table S5, Supplement 2) and at months 2 and 24 when examined at discrete follow-up points (Table S4, Supplement 2). Lower NSB was associated with greater concurrent decentering, and lower post-treatment NSB broadly predicted greater decentering across follow-up (Table S6–8, Supplement 2).

However, no direct associations between behavioral NSB and relapse status were observed. Exploratory analyses of recognition memory performance were conducted in a subset participant (*n* = 65), but no significant associations with relapse or residual symptoms were detected (Tables S9–11, Supplement 2).

### 3.2 Frontal DMN–SN dysphoric self-referential activity and connectivity as markers of relapse vulnerability

We next tested the preregistered ROI-based hypotheses concerning dysphoric self-referential activity and connectivity within anatomically defined frontal DMN and SN regions. Greater dysphoric self-referential activity within both frontal DMN and SN regions was associated with relapse status. Post hoc analyses indicated that relapsers exhibited higher baseline dysphoric activity in both networks compared with non-relapsers. A significant Relapse × Time interaction was observed for frontal DMN activity only (Tables S12–13, Supplement 2). However, neither frontal DMN nor SN dysphoric activity independently predicted relapse risk in survival analyses (Table S14, Supplement 2).

No significant associations were observed between relapse status and between-network DMN–SN connectivity, within-DMN connectivity, or within-SN connectivity (Tables S15–17, Supplement 2).

### 3.3 Static markers of relapse vulnerability and dynamic markers of treatment response

**Fig. 3:**
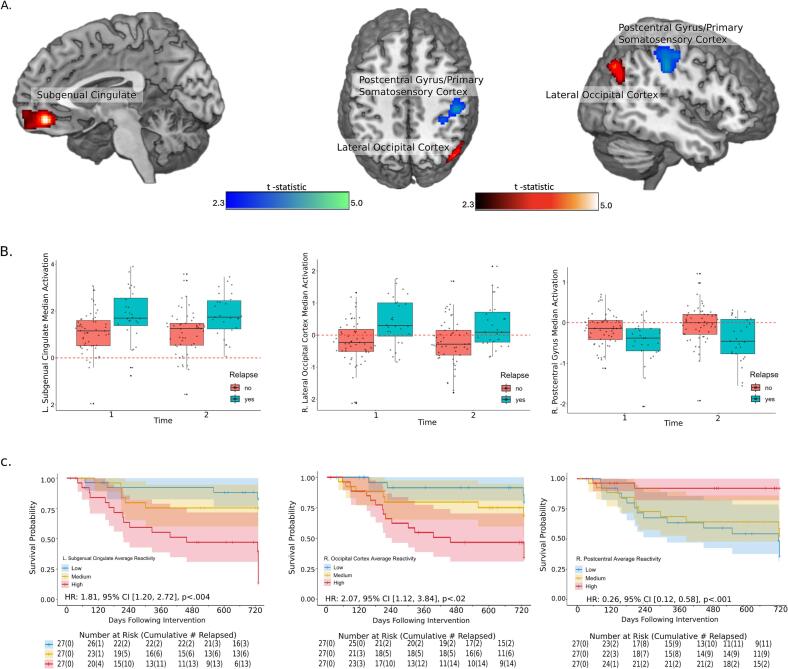
Static neural markers of relapse vulnerability during self-referential processing (Self > Case). Panel A shows group-level activation maps illustrating that relapse is associated with higher activation in the left subgenual cingulate and right lateral occipital cortex (red), and lower activation in the right postcentral gyrus/primary somatosensory cortex (blue). Panel B presents mean activation within each region of interest (ROI) across baseline and post-intervention sessions, with errors bars reflecting between-subject variability. Panel C displays survival curves over the follow-up period stratified by tertiles of average reactivity (Low, Medium, High) in the left subgenual cingulate, right occipital cortex, and right postcentral gyrus. Crosshatches indicate participants censored due to relapse or loss to follow-up. (For interpretation of the references to color in this figure legend, the reader is referred to the web version of this article.)

We next conducted whole-brain analyses to identify relapse-related neural markers beyond the a priori frontal DMN/SN ROI tests. Fig. 3 shows differences in whole-brain, self-referential activity by relapse status (Table S18, Supplement 2). Relapsers exhibited greater activation in the left subgenual cingulate (optimal cutpoint = 1.60; Table S19 and Fig. S3A, Supplement 2) and right lateral occipital cortex (optimal cutpoint =0.11; Table S20 and Fig. S3B, Supplement 2), and greater deactivation in the right postcentral gyrus (optimal cutpoint = −0.14; Table S21 and Fig. S3C, Supplement 2) than non-relapsers. Cox regression (Table S22, Supplement 2) revealed the Hazard Ratios (HR) associated with these patterns, with substantially increased relapse risk from elevated activity in the left subgenual cingulate (HR = 1.81) and right lateral occipital cortex (HR = 2.07), and from greater deactivation in the right postcentral gyrus (HR = 3.85).

Dynamic markers of treatment related response associated with non-relapse revealed attenuated reductions over time within somatosensory and associative cortices (Table S23, Supplement 2). Although longitudinal changes were observed, no significant treatment main effects, treatment-by-time effects, or post-intervention treatment effects within the non-relapse group were detected, suggesting that these longitudinal neural changes were observed across the intervention context. Each ROIs' Group and Time effects, optimal cut-points, and HR for relapse risk are available in Tables S24–30 and Fig. S4 (Supplement 2).

Exploratory general linear models examined whether static and dynamic neural markers predicted post-treatment residual symptoms while controlling for baseline residual symptoms. Greater activation in right lateral occipital cortex was associated with increases in residual symptoms over treatment (*b* = 0.28, *SE* = 0.12, *p* = .02). No other associations were observed.

**Fig. 4:**
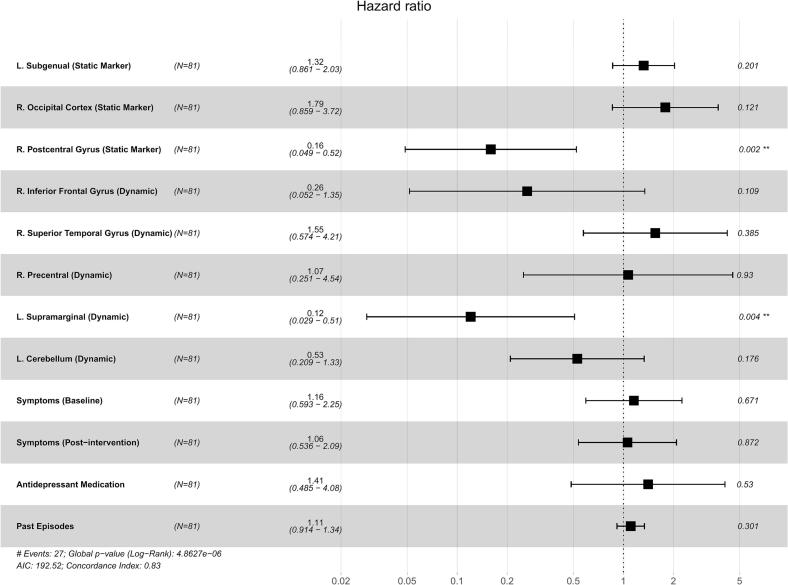
Combined Cox Model of Static and Dynamic Neural Relapse Markers. Hazard ratios are shown from the combined Cox regression model predicting relapse, incorporating both static neural markers (left subgenual cingulate, right occipital cortex, right postcentral gyrus) and dynamic markers reflecting change over the intervention period, right inferior frontal gyrus, right superior temporal gyrus, right precentral gyrus, left supramarginal gyrus, left cerebellum). The model was controlled for past depressive episodes, concurrent depressive symptoms, and antidepressant medication status.

A combined survival model incorporated both static and dynamic ROIs, adjusting for ADM, past episodes, and baseline and post-treatment residual symptoms (Fig. 4). Higher mean activation in the right postcentral gyrus and greater increases over time in the left supramarginal gyrus were the sole indicators associated with reduced relapse risk (Table S31, Supplement 2).

### 3.4 Additional analyses

As an exploratory follow-up to the whole-brain static relapse markers, we conducted whole-brain psychophysiological interaction analyses to test connectivity differences during self-referential processing and as a function of relapse. Task-modulated functional connectivity was estimated using generalized psychophysiological interaction (gPPI) analyses implemented in SPM12. gPPI models were run separately for each seed ROI, with seed time series extracted as the first eigenvariate. Connectivity was modeled condition-specific (i.e., self, other, case, and fixation conditions). The primary connectivity contrast of interest was Self > Case, yielding seed-specific whole-brain connectivity maps that were entered into second-level analyses to examine effects of relapse status. No significant group differences were observed (Table S32, Supplement 2).

## 4 Discussion

We conducted a prospective neuroimaging investigation of prophylactic psychotherapy, aimed at clarifying how self-referential processing contributes to relapse vulnerability. Greater NSB was associated with greater symptom burden and lower decentering both at post-treatment and across the two-year follow-up. Notably, endorsement patterns did not directly predict future depressive episodes.

Self-criticism, which consisted of affirming negative traits and denying positive traits, engaged a broader set of prefrontal and posterior regions in the whole-brain analysis, including medial prefrontal, orbitofrontal/insula, posterior cingulate, and occipital regions, consistent with models linking depression to exaggerated self-focused and affectively salient processing (Lemogne et al., 2009, Lemogne et al., 2010, Lemogne et al., 2012). This pattern included left DLPFC activation, a core node of the CEN, providing exploratory support for the third arm of the dorsal nexus hypothesis. DLPFC recruitment during negative self-referential processing may reflect increased cognitive control demands or compensatory regulatory effort in the face of dysphoric self-focus, consistent with accounts linking prefrontal control circuitry to the maintenance of negative attentional bias in depression (De Raedt and Koster, 2010; Johnstone et al., 2007). Within preregistered frontal DMN and SN regions, dysphoric self-referential activity was elevated among relapsers compared with non-relapsers. However, these specific ROI measures did not independently predict time to relapse in survival analyses, suggesting that frontal dysphoric self-referential activity may reflect relapse-related group differences without robustly forecasting episode timing on its own.

The divergence between behavioral NSB and frontal brain activity, where only frontal brain activity distinguished relapse status, may reflect the operation of partially distinct mechanisms underlying negative self-referential processing. Attentional bias toward negative self-relevant information, indexed behaviorally by endorsement rates, may primarily reflect a conscious, reportable tendency that tracks concurrent symptom burden without necessarily driving episode recurrence. By contrast, the neural activity patterns that predicted relapse, particularly sustained prefrontal recruitment during self-referential processing, may more closely reflect internal elaboration, an automatic amplification and maintenance of negative self-relevant content that contributes more directly to relapse risk (Disner et al., 2011; Watkins and Teasdale, 2004). State-level dysphoria, induced in the present study as part of the broader task protocol, may have further contributed to both neural and behavioral responses, making it difficult to fully separate vulnerability-related processing from mood-congruent activation. These distinctions suggest that behavioral and neural indicators of self-referential bias are best understood as complementary rather than redundant markers of depression vulnerability.

Whole brain analyses further characterized relapse vulnerability, specifically implicating the left subgenual cingulate, a region consistently implicated in persistent depressive risk and targeted in therapeutic interventions (Benschop et al., 2022; Drevets et al., 2008; Mayberg et al., 2005). Relapsers also showed greater activation in right lateral occipital cortex, a region more recently implicated in MDD as a site of perceptual disruption (Li et al., 2025; Wu et al., 2023), and whose baseline reactivity additionally predicted worsening residual symptoms across treatment- consistent with evidence that lateral occipital cortex hyperactivation reflects difficulty disengaging from negative emotional content in depression (Colich et al., 2017). Together, these findings align with attentional accounts of depression, where excessive engagement with negative schema-relevant information is maintained by prefrontal and posterior perceptual circuitry (De Raedt and Koster, 2010; Johnstone et al., 2007; Pizzagalli and Roberts, 2022).

By contrast, deficits in self-affirmation were associated with aberrant somatosensory processing. Greater positive and lower negative trait endorsement recruited the posterior somatosensory cortex, and relapsers consistently showed greater deactivations in this region, with treatment-related attenuation of these deactivations in somatosensory-parietal regions characterizing the non-relapse trajectory. Reduced somatosensory activity during self-referential processing may reflect diminished access to bodily and sensory cues that ground self-experience in the present moment (Farb et al., 2010, Farb et al., 2015), shifting self-evaluation toward more enduring, narrative forms of self-awareness that support rigid rather than flexible updating of self-relevant information (Farb et al., 2007, Farb et al., 2015).

This interpretation aligns with broader theoretical accounts proposing that self-representation is grounded in embodied sensory experience and ongoing bodily signals, such that accessible interoceptive representations support moment-to-moment adaptation while reduced access contributes to dysregulated self-processing(Paulus and Stein, 2010; Seth, 2013; Seth and Friston, 2016). Consistent with this view, somatosensory attention cultivated through mindfulness training may help regulate mind-wandering and support metacognitive flexibility (Kerr et al., 2013). Decentering, a construct linked to psychological distance and perspective taking (Segal et al., 2019), was negatively associated with depressive symptoms and self-critical endorsement but positively associated with self-affirmation, suggesting that greater somatosensory engagement over treatment may be expressed psychologically as an increased capacity to relate to self-relevant experience as contextual and changing, rather than as fixed evidence of negative self-worth.

Our combined survival model suggests that somatosensory markers explained unique variance in relapse risk when static and longitudinal treatment-related neural markers were considered together. Higher right postcentral activation and greater increases in left supramarginal activation predicted *lower* relapse likelihood, even after adjusting for clinical covariates. The left supramarginal gyrus may be especially relevant as a dynamic marker because treatment-related attenuated deactivations were associated with lower relapse risk. This region has been implicated in multisensory integration and somatosensory feedback (Isernia et al., 2023; Proske and Gandevia, 2012), and has also been implicated in emotion processing and regulation in psychiatric populations (Gong et al., 2020; Madeira et al., 2020). Accordingly, increased supramarginal engagement over treatment may reflect improved multisensory and embodied processing that supports self-affirmation and reduces vulnerability to relapse. In contrast, prefrontal markers, although predictive in isolation, no longer contributed significantly when somatosensory markers were included, suggesting that somatosensory markers may capture unique protective variance in this sample that is not fully accounted for by prefrontal indices alone.

These findings have important clinical implications. Much of the treatment literature focuses on reducing prefrontal engagement with negative content, challenging self-critical thoughts, reducing rumination, or modulating subgenual activity. Our results suggest that strengthening access to somatosensory representations may be at least as relevant for relapse prevention, and that the failure of prefrontal markers to add predictive value beyond somatosensory markers in the combined model suggests these systems may reflect partially independent pathways to resilience. Both WB-CT and MBCT emphasize updating negative beliefs and cultivating embodied awareness (Fava, 2016; Segal et al., 2012), and the neural pattern observed here, stronger somatosensory engagement predicting reduced relapse risk, provides a mechanistic account for why embodied components of these interventions may be particularly relevant for long-term protection against recurrence.

Contrary to expectations, relapse status was not associated with DMN–SN functional connectivity, despite prominent models proposing that depression vulnerability reflects hyperconnectivity between these networks (Bertocci et al., 2023; Sheline et al., 2010). Elevated prefrontal activation during self-referential processing may be more critical for relapse vulnerability than heightened inter-network coupling per se. Connectivity abnormalities may be less relevant indicators of NSB, instead reflecting broader or more context-general features of depression.

### 4.1 Limitations

The study has several limitations. First, the absence of a healthy control group limits inferences about whether the identified neural markers are depression-specific vulnerability or more general characteristics of negative self-referential processing. Inclusion of healthy comparison samples in future work would help establish disorder specificity. Second, psychiatric and medical comorbidities were not assessed. Given their high prevalence in MDD (Calarco and Lobo, 2021; Gold et al., 2020; Kalin, 2020), and known effects on neural functioning (Kunas et al., 2019; Zheng et al., 2022), and relapse risk (Nunes et al., 2024; Wang et al., 2021), systematic assessment of comorbidities would be important for evaluating generalizability across clinical presentations. Third, self-referential processing was operationalized using a Self > Case contrast, rather than the more conventional Self > Other + Case contrast. However, sensitivity analyses showed near-perfect convergence of ROI estimates across these functional definitions for frontal DMN and SN regions (Fig. S5, Supplement 2), suggesting that fewer task conditions may offer a parsimonious and reproducible analytic approach. Fourth, the sample was demographically homogeneous—predominantly Caucasian and university educated—limiting generalizability. Racialized and socioeconomically disadvantaged groups face disproportionate burdens of depression and differential relapse risk (Bailey et al., 2019; Cénat et al., 2025; Cohen et al., 2020). Increasing representation of racialized and lower-education populations is therefore critical for ensuring clinical and translational relevance. Fifth, because dysphoric mood induction was interleaved with the self-referential task, state-related influences cannot be fully ruled out. Therefore, the present findings are best interpreted as reflecting dysphoric self-referential processing in a context where trait-like vulnerability and induced negative state may both contribute. Sixth, the present study focused on preregistered frontal DMN and SN ROIs; formal tests of CEN involvement in relapse vulnerability, suggested by exploratory DLPFC findings and the broader dorsal nexus account, remain an important direction for future work. Seventh, our analysis of treatment-related changes in somatosensory responses during self-referential processing was exploratory and was not specified in our preregistration. The static relationship between somatosensory deactivation and relapse was a replicated finding in this cohort during dysphoric mood induction (Farb et al., 2022); the treatment-related somatosensory change reported here, however, was not preregistered and should be regarded as hypothesis-generating. Therefore, future confirmatory replication studies in an independent sample are needed. Finally, this study focused exclusively on self-referential processing and treatment-related change within MBCT and WB-CT. Other cognitive and emotional processes relevant to depression vulnerability, such as reward sensitivity (Berry et al., 2019; Kujawa, 2024) and autobiographical memory (Weiss-Cowie et al., 2023), remain to be evaluated using similar longitudinal designs.

## 5 Conclusions

This study challenges the traditional prefrontal-centric view of depression vulnerability by demonstrating that relapse risk may depend as much on insufficient recruitment of somatosensory systems as on heightened negative evaluative processing. Interventions that enhance embodied awareness, sensory integration, and bottom-up updating may therefore represent promising targets for future treatment optimization.

## Disclosure of competing interest

LW, JL, and NF reported no financial interests or potential conflicts of interest. ZS disclosed book royalties from Guilford Press, workshop fees from the Centre for Mindfulness studies, and revenue from online sales at Mindful Noggin Inc., which are all related to his work as a co-founder of Mindfulness-Based Cognitive Therapy (MBCT).

## Data sharing statement

Deidentified behavioral task and clinical data are publicly available on the Open Science Framework (https://osf.io/s4n8j). The authors are happy to provide deidentified fMRI-derived data upon request. Access to raw fMRI data requires execution of a material transfer agreement and approval by the originating institution.

## CRediT authorship contribution statement

**Liliana C. Wu:** Writing – review & editing, Writing – original draft, Visualization, Methodology, Investigation, Formal analysis, Data curation, Conceptualization. **Jordan L. Livingston:** Writing – review & editing, Formal analysis, Data curation. **Zindel V. Segal:** Writing – review & editing, Supervision, Resources, Methodology, Investigation, Funding acquisition, Conceptualization. **Norman A.S. Farb:** Writing – review & editing, Writing – original draft, Validation, Supervision, Resources, Project administration, Methodology, Investigation, Funding acquisition, Formal analysis, Data curation, Conceptualization.

## Data Availability

Data will be made available on request.

## Appendix A Supplementary data

Supplementary material 1 IMAGE 1

Supplementary material 2 IMAGE 2

## References

[bb0005] Bailey R.K., Mokonogho J., Kumar A. (2019). Racial and ethnic differences in depression: current perspectives. Neuropsychiatr. Dis. Treat..

[bb0010] Benschop L., Vanhollebeke G., Li J., Leahy R.M., Vanderhasselt M.-A., Baeken C. (2022). Reduced subgenual cingulate–dorsolateral prefrontal connectivity as an electrophysiological marker for depression. Sci. Rep..

[bb0015] Berry M.P., Tanovic E., Joormann J., Sanislow C.A. (2019). Relation of depression symptoms to sustained reward and loss sensitivity. Psychophysiology.

[bb0020] Bertocci M.A., Afriyie-Agyemang Y., Rozovsky R., Iyengar S., Stiffler R., Aslam H.A., Bebko G., Phillips M.L. (2023). Altered patterns of central executive, default mode and salience network activity and connectivity are associated with current and future depression risk in two independent young adult samples. Mol. Psychiatry.

[bb0025] Birn R.M. (2012). The role of physiological noise in resting-state functional connectivity. NeuroImage.

[bb0030] Bradley B., Mathews A. (1983). Negative self-schemata in clinical depression. Br. J. Clin. Psychol..

[bb0035] Brockmeyer T., Zimmermann J., Kulessa D., Hautzinger M., Bents H., Friederich H.-C., Herzog W., Backenstrass M. (2015). Me, myself, and I: self-referent word use as an indicator of self-focused attention in relation to depression and anxiety. Front. Psychol..

[bb0040] Buckner R.L., Carroll D.C. (2007). Self-projection and the brain. Trends Cogn. Sci..

[bb0045] Burcusa S.L., Iacono W.G. (2007). Risk for recurrence in depression. Clin. Psychol. Rev..

[bb0050] Burklund L.J., Creswell J.D., Irwin M.R., Lieberman M.D. (2014). The common and distinct neural bases of affect labeling and reappraisal in healthy adults. Front. Psychol..

[bb0055] Calarco C.A., Lobo M.K. (2021). Depression and substance use disorders: clinical comorbidity and shared neurobiology. Int. Rev. Neurobiol..

[bb0060] Cénat J.M., Moshirian Farahi S.M.M., Dalexis R.D. (2025). Prevalence and correlates of severe depressive symptoms among Arab, Asian, black, indigenous, white, and mixed-race individuals in Canada: a population-based study. Lancet Reg. Health - Am..

[bb0065] Chou T., Deckersbach T., Dougherty D.D., Hooley J.M. (2023). The default mode network and rumination in individuals at risk for depression. Soc. Cogn. Affect. Neurosci..

[bb0070] Cohen A.K., Nussbaum J., Weintraub M.L.R., Nichols C.R., Yen I.H. (2020). Association of adult depression with educational attainment, aspirations, and expectations. Prev. Chronic Dis..

[bb0075] Colich N.L., Ho T.C., Foland-Ross L.C., Eggleston C., Ordaz S.J., Singh M.K., Gotlib I.H. (2017). Hyperactivation in cognitive control and visual attention brain regions during emotional interference in adolescent depression. Biol. Psychiatry: Cognit. Neurosci. Neuroimaging.

[bb0080] De Raedt R., Koster E.H.W. (2010). Understanding vulnerability for depression from a cognitive neuroscience perspective: a reappraisal of attentional factors and a new conceptual framework. Cogn. Affect. Behav. Neurosci..

[bb0085] (2017). Depression and Other Common Mental Disorders: Global Health Estimates (WHO/MSD/MER/2017.2).

[bb0090] Derry P.A., Kuiper N.A. (1981). Schematic processing and self-reference in clinical depression. J. Abnorm. Psychol..

[bb0095] Disner S.G., Beevers C.G., Haigh E.A.P., Beck A.T. (2011). Neural mechanisms of the cognitive model of depression. Nat. Rev. Neurosci..

[bb0100] Drevets W.C., Savitz J., Trimble M. (2008). The subgenual anterior cingulate cortex in mood disorders. CNS Spectr..

[bb0105] Dunlop B.W., Cha J., Choi K.S., Nemeroff C.B., Craighead W.E., Mayberg H.S. (2023). Functional connectivity of salience and affective networks among remitted depressed patients predicts episode recurrence. Neuropsychopharmacology.

[bb0110] Eaton W.W., Shao H., Nestadt G., Lee B.H., Bienvenu O.J., Zandi P. (2008). Population-based study of first onset and chronicity in major depressive disorder. Arch. Gen. Psychiatry.

[bb0115] Farb N. (2010). Minding one’s emotions: mindfulness training alters the neural expression of sadness. Emotion.

[bb0120] Farb N.A.S., Segal Z.V., Mayberg H., Bean J., McKeon D., Fatima Z., Anderson A.K. (2007). Attending to the present: mindfulness meditation reveals distinct neural modes of self-reference. Soc. Cogn. Affect. Neurosci..

[bb0125] Farb N.A.S., Anderson A.K., Mayberg H., Bean J., McKeon D., Segal Z.V. (2010). Minding one’s emotions: mindfulness training alters the neural expression of sadness. Emotion.

[bb0130] Farb N.A.S., Anderson A.K., Bloch R.T., Segal Z.V. (2011). Mood-linked responses in medial prefrontal cortex predict relapse in patients with recurrent unipolar depression. Biol. Psychiatry.

[bb0135] Farb N., Daubenmier J., Price C.J., Gard T., Kerr C., Dunn B.D., Klein A.C., Paulus M.P., Mehling W.E. (2015). Interoception, contemplative practice, and health. Front. Psychol..

[bb0140] Farb N., Anderson A., Ravindran A., Hawley L., Irving J., Mancuso E., Gulamani T., Williams G., Ferguson A., Segal Z.V. (2018). Prevention of relapse/recurrence in major depressive disorder with either mindfulness-based cognitive therapy or cognitive therapy. J. Consult. Clin. Psychol..

[bb0145] Farb N.A.S., Desormeau P., Anderson A.K., Segal Z.V. (2022). Static and treatment-responsive brain biomarkers of depression relapse vulnerability following prophylactic psychotherapy: evidence from a randomized control trial. NeuroImage Clin..

[bb0150] Fava G.A. (2016). Well-being therapy: current indications and emerging perspectives. Psychother. Psychosom..

[bb0155] Ford C.G., Kiken L.G., Haliwa I., Shook N.J. (2023). Negatively biased cognition as a mechanism of mindfulness: a review of the literature. Curr. Psychol..

[bb0160] Gold S.M., Köhler-Forsberg O., Moss-Morris R., Mehnert A., Miranda J.J., Bullinger M., Steptoe A., Whooley M.A., Otte C. (2020). Comorbid depression in medical diseases. Nat. Rev. Dis. Primers.

[bb0165] Gong J., Wang J., Qiu S., Chen P., Luo Z., Wang J., Huang L., Wang Y. (2020). Common and distinct patterns of intrinsic brain activity alterations in major depression and bipolar disorder: voxel-based meta-analysis. Transl. Psychiatry.

[bb0170] Gotlib I.H., Kasch K.L., Traill S., Joormann J., Arnow B.A., Johnson S.L. (2004). Coherence and specificity of information-processing biases in depression and social phobia. J. Abnorm. Psychol..

[bb0175] Hamilton J.P., Etkin A., Furman D.J., Lemus M.G., Johnson R.F., Gotlib I.H. (2012). Functional neuroimaging of major depressive disorder: a meta-analysis and new integration of baseline activation and neural response data. Am. J. Psychiatry.

[bb0180] Herrman H., Patel V., Kieling C., Berk M., Buchweitz C., Cuijpers P., Furukawa T.A., Kessler R.C., Kohrt B.A., Maj M., McGorry P., Reynolds C.F., Weissman M.M., Chibanda D., Dowrick C., Howard L.M., Hoven C.W., Knapp M., Mayberg H.S., Wolpert M. (2022). Time for united action on depression: a lancet-world psychiatric association commission. Lancet.

[bb0185] Isernia S., Blasi V., Baglio G., Cabinio M., Cecconi P., Rossetto F., Cazzoli M., Blasi F., Bruckmann C., Giunco F., Sorbi S., Clerici M., Baglio F. (2023). The key role of depression and supramarginal gyrus in frailty: a cross-sectional study. Front. Aging Neurosci..

[bb0190] Jiang Y. (2024). A theory of the neural mechanisms underlying negative cognitive bias in major depression. Front. Psychol..

[bb0195] Johnstone T., van Reekum C.M., Urry H.L., Kalin N.H., Davidson R.J. (2007). Failure to regulate: counterproductive recruitment of top-down prefrontal-subcortical circuitry in major depression. J. Neurosci..

[bb0200] Kalin N.H. (2020). The critical relationship between anxiety and depression. Am. J. Psychiatry.

[bb0205] Kerr C.E., Sacchet M.D., Lazar S.W., Moore C.I., Jones S.R. (2013). Mindfulness starts with the body: somatosensory attention and top-down modulation of cortical alpha rhythms in mindfulness meditation. Front. Hum. Neurosci..

[bb0210] Kessler R.C., Petukhova M., Sampson N.A., Zaslavsky A.M., Wittchen H.-U. (2012). Twelve-month and lifetime prevalence and lifetime morbid risk of anxiety and mood disorders in the United States. Int. J. Methods Psychiatr. Res..

[bb0215] Kong Y., Jenkinson M., Andersson J., Tracey I., Brooks J.C.W. (2012). Assessment of physiological noise modelling methods for functional imaging of the spinal cord. NeuroImage.

[bb0220] Kujawa A. (2024). Reduced reward responsiveness and depression vulnerability: consideration of social contexts and implications for intervention. Psychophysiology.

[bb0225] Kunas S.L., Yang Y., Straube B., Kircher T., Gerlach A.L., Pfleiderer B., Arolt V., Wittmann A., Stroehle A., Wittchen H.-U., Lueken U. (2019). The impact of depressive comorbidity on neural plasticity following cognitive-behavioral therapy in panic disorder with agoraphobia. J. Affect. Disord..

[bb0230] Lemogne C., le Bastard G., Mayberg H., Volle E., Bergouignan L., Lehéricy S., Allilaire J.-F., Fossati P. (2009). In search of the depressive self: extended medial prefrontal network during self-referential processing in major depression. Soc. Cogn. Affect. Neurosci..

[bb0235] Lemogne C., Mayberg H., Bergouignan L., Volle E., Delaveau P., Lehéricy S., Allilaire J.-F., Fossati P. (2010). Self-referential processing and the prefrontal cortex over the course of depression: a pilot study. J. Affect. Disord..

[bb0240] Lemogne C., Delaveau P., Freton M., Guionnet S., Fossati P. (2012). Medial prefrontal cortex and the self in major depression. J. Affect. Disord..

[bb0245] LeMoult J., Kircanski K., Prasad G., Gotlib I.H. (2017). Negative self-referential processing predicts the recurrence of major depressive episodes. Clin. Psychol. Sci..

[bb0250] Li Y., Li M., Wei D., Kong X., Du X., Hou X., Sun J., Qiu J. (2017). Self-referential processing in unipolar depression: distinct roles of subregions of the medial prefrontal cortex. Psychiatry Res. Neuroimaging.

[bb0255] Li J., Tan Y., Zheng Z., Feng C., Fang W., Huang X., Lin S., So K.-F., Huang L., Ren C., Tao Q. (2025). Reduced neural suppression at occipital cortex in subthreshold depression. Transl. Psychiatry.

[bb0260] Lou Y., Lei Y., Mei Y., Leppänen P.H.T., Li H. (2019). Review of abnormal self-knowledge in major depressive disorder. Front. Psychol..

[bb0265] Madeira N., Duarte J.V., Martins R., Costa G.N., Macedo A., Castelo-Branco M. (2020). Morphometry and gyrification in bipolar disorder and schizophrenia: a comparative MRI study. NeuroImage Clin..

[bb0270] Mayberg H.S., Lozano A.M., Voon V., McNeely H.E., Seminowicz D., Hamani C., Schwalb J.M., Kennedy S.H. (2005). Deep brain stimulation for treatment-resistant depression. Neuron.

[bb0275] Monroe S.M., Harkness K.L. (2011). Recurrence in major depression: a conceptual analysis. Psychol. Rev..

[bb0280] Nejad A.B., Fossati P., Lemogne C. (2013). Self-referential processing, rumination, and cortical midline structures in major depression. Front. Hum. Neurosci..

[bb0285] Nunes A., Pavlova B., Cunningham J.E.A., Nuñez J.-J., Quilty L.C., Foster J.A., Harkness K.L., Ho K., Lam R.W., Li Q.S., Milev R., Rotzinger S., Soares C.N., Taylor V.H., Turecki G., Kennedy S.H., Frey B.N., Rudzicz F., Uher R. (2024). Depression-anxiety coupling strength as a predictor of relapse in major depressive disorder: a CAN-BIND wellness monitoring study report. J. Affect. Disord..

[bb0290] Paulus M.P., Stein M.B. (2010). Interoception in anxiety and depression. Brain Struct. Funct..

[bb0295] Pizzagalli D.A., Roberts A.C. (2022). Prefrontal cortex and depression. Neuropsychopharmacology.

[bb0300] Proske U., Gandevia S.C. (2012). The proprioceptive senses: their roles in signaling body shape, body position and movement, and muscle force. Physiol. Rev..

[bb0305] Pyszczynski T., Holt K., Greenberg J. (1987). Depression, self-focused attention, and expectancies for positive and negative future life events for self and others. J. Pers. Soc. Psychol..

[bb2000] R Core Team (2023). https://www.R-project.org/.

[bb0310] Ray D., Bezmaternykh D., Mel’nikov M., Friston K.J., Das M. (2021). Altered effective connectivity in sensorimotor cortices is a signature of severity and clinical course in depression. Proc. Natl. Acad. Sci. USA.

[bb0315] Sabbah S.G., Northoff G. (2025). The self in depression and anxiety as a transdiagnostic and differential-diagnostic neural marker: a systematic review. Neurosci. Biobehav. Rev..

[bb0320] Segal Z., Williams M., Teasdale J. (2012).

[bb0325] Segal Z.V., Anderson A.K., Gulamani T., Williams L.A.D., Desormeau P., Ferguson A., Walsh K., Farb N.A.S. (2019). Practice of therapy acquired regulatory skills and depressive relapse/ recurrence prophylaxis following cognitive therapy or mindfulness based cognitive therapy. J. Consult. Clin. Psychol..

[bb0330] Seth A.K. (2013). Interoceptive inference, emotion, and the embodied self. Trends Cogn. Sci..

[bb0335] Seth A.K., Friston K.J. (2016). Active interoceptive inference and the emotional brain. Philos. Trans. R. Soc. B.

[bb0340] Sheline Y.I., Barch D.M., Price J.L., Rundle M.M., Vaishnavi S.N., Snyder A.Z., Mintun M.A., Wang S., Coalson R.S., Raichle M.E. (2009). The default mode network and self-referential processes in depression. Proc. Natl. Acad. Sci..

[bb0345] Sheline Y.I., Price J.L., Yan Z., Mintun M.A. (2010). Resting-state functional MRI in depression unmasks increased connectivity between networks via the dorsal nexus. Proc. Natl. Acad. Sci..

[bb0350] Thiele C., Hirschfeld G. (2021). Cutpointr: improved estimation and validation of optimal cutpoints in R. J. Stat. Softw..

[bb0355] Timbremont B., Braet C. (2004). Cognitive vulnerability in remitted depressed children and adolescents. Behav. Res. Ther..

[bb0360] Wang M., Pinilla G., Leung C., Peddada A., Yu E., Akmal S., Cha Y., Dyson L., Kumar A., Kaplin A. (2021). Relapse risk factors for patients with comorbid affective disorders and substance abuse disorders from an intensive treatment unit. Am. J. Addict..

[bb0365] Watkins E., Teasdale J.D. (2004). Adaptive and maladaptive self-focus in depression. J. Affect. Disord..

[bb0370] Weiss-Cowie S., Verhaeghen P., Duarte A. (2023). An updated account of overgeneral autobiographical memory in depression. Neurosci. Biobehav. Rev..

[bb0375] Wu F., Lu Q., Kong Y., Zhang Z. (2023). A comprehensive overview of the role of visual cortex malfunction in depressive disorders: opportunities and challenges. Neurosci. Bull..

[bb0380] Zheng C.J., Van Drunen S., Egorova-Brumley N. (2022). Neural correlates of co-occurring pain and depression: an activation-likelihood estimation (ALE) meta-analysis and systematic review. Transl. Psychiatry.

