# Supplementary material for: Functional brain biomarkers of self-referential bias in remitted depressed outpatients: a randomized controlled trial
Source: Neuroimage Clin. 2026 Jun 19;51:104025. doi: 10.1016/j.nicl.2026.104025 (PMC13316189; doi:10.1016/j.nicl.2026.104025)
Supplement: Supplementary file 1 — Supplementary material 1 [file mmc1.docx]

Functional Brain Biomarkers of Self-Referential Bias and Depression Vulnerability in Remitted Depressed Individuals

**Registration Type**

OSF Preregistration

**Registration DOI**

[**10.17605/OSF.IO/S4N8J**](https://doi.org/10.17605/OSF.IO/S4N8J)

**Date Created**

Nov 7, 2024, 1:51 PM

**Date registered**

Nov 7, 2024, 1:51 PM

# Study Information

## Hypotheses

The current project focuses on analyzing both neuroimaging and behavioral data from a self-referential encoding task (SRET) that was collected in a longitudinal, randomized controlled study assessing depression risk and relapse.

The SRET consisted of 3 encoding conditions (self, other, case), and 2 word valences (positive, negative), and was performed at 2 time points (baseline, post-intervention). This yielded both endorsement data (yes, no) and recognition data (accurate, inaccurate).

This study will investigate how strongly negative self-referential bias—evidenced by endorsement, memory, and neural activation patterns—is associated with depression vulnerability, particularly in predicting relapse among remitted depressed individuals.

**Manipulation Checks:**

Memory effects:

Endorsed > non-endorsed (levels of processing)

Self > other > case (levels of processing)

Negative > Positive (negativity bias)

Self-enhancement bias: Generally more positive > negative endorsement

Neural effects (self-reference as DMN activation in the brain):

Self > Other + Case → greater default mode network (DMN) activation

Endorsed > non-endorsed self words → greater DMN activation

**Main Hypotheses:**

Endorsement. We expect that greater endorsement of negative words compared to positive words as a function of self (relative to other and case) will be associated with future relapse while controlling for residual symptoms, anti-depression medication, past depression episodes, interoceptive dysfunction, and decentering

Memory. We expect that better Recognition memory for endorsed negative words than endorsed positive words for self (relative to other and case), will be associated with future relapse while controlling for residual symptoms, anti-depression medication, past depression episodes, interoceptive dysfunction, and decentering

Neural activity- when comparing self-endorsed negative words to self-endorsed positive words, and while controlling for residual symptoms, anti-depression medication, past depression episodes, interoceptive dysfunction, and decentering:

- Self-referential network (DMN). We expect greater activity in the frontal aspect of the DMN (i.e., the dmPFC) in relapsers compared to non-relapsers.
- Dorsal salience network (SN). We expect greater activity in the frontal aspect of the SN (i.e., the dACC) in relapsers compared to non-relapsers.
- Dorsal nexus. We expect more positive (or less negative) functional connectivity between the prefrontal DMN and SN regions in relapsers compared to non-relapsers.

# Design Plan

## Study type

Experiment - A researcher randomly assigns treatments to study subjects, this includes field or lab experiments. This is also known as an intervention experiment and includes randomized controlled trials.

## Blinding

For studies that involve human subjects, they will not know the treatment group to which they have been assigned.

## Is there any additional blinding in this study?

No data

## Study design

The study was conducted using a longitudinal, two-group, randomized controlled design. Remitted depressed outpatients (N =85) were randomized to 8 weeks of Cognitive Therapy with a Well-Being focus (WB-CT) or Mindfulness Based Cognitive Therapy (MBCT). Participants were assessed at 1) baseline, 2) post-treatment (8 weeks), and 3) end of two-year follow up. During each fMRI session, participants completed two blocks of the SRET task consisting of 48 task trials and 32 fixation trials.

No files selected

## Randomization

Randomization occurred at the participant level following baseline assessment. Participants were randomly assigned to condition in blocks of four using computer-generated, quasi-random numbers.

# Sampling Plan

## Existing Data

Registration prior to analysis of the data

## Explanation of existing data

Previously published work has examined the effect of treatment on relapse status ( Farb et al., 2018; Segal et al., 2018), as well as the relationship between dysphoric reactivity and relapse following prophylactic intervention (Farb et al., 2022). Moreover, a lab member conducted a preliminary analysis investigating the role of self-referential bias as a predictive behavioral marker for depression relapse. However, functional activation in the brain related to self-referential processes has not been analyzed.

## Data collection procedures

Participants were screened for inclusion and exclusion criteria and provided informed consent. Inclusion criteria included:

1) absence of major depressive disorder (MDD) at time of screening (determined using DSM IV and HRSD-17), 2) at least one previous episode of MDD, 3) 18-65 years of age, 4) English speaking, and 5) qualification for MRI safety standards. Exclusion criteria included: 1) diagnosis of bipolar disorder, substance abuse disorder, schizophrenia, or borderline personality disorder at time of screening, 2) currently receiving cognitive therapy for depression, 3) meditating more than once per week, or 4) participating in yoga more than twice per week.

All data were acquired as part of an RCT evaluating the effectiveness of MBCT and WB-CT on depression relapse prevention. fMRI sessions were conducted at baseline, at the end of an 8-week treatment, and at a 2-year follow-up.

During each fMRI session, participants completed two runs of the self-referential bias task (presented in interleaved order with the film task), each consisting of 80 trials (48 task trials and 32 fixation trials). On task trials, participants viewed different cue (self, other, case) + trait word (positive and negative) combinations and were asked to respond (yes or no) to each trial. Reaction times and button responses were recorded. Trials across the conditions were presented using an event-related design, with each trial lasting 3.5 seconds. After each scan session, participants were asked to engage in a recognition task in which they were presented with trait words, one at a time, and indicated whether each word was “new” (had not been presented in the scanner) or “old” (had been presented in the scanner).

fMRI data acquisition

As described in Farb et al., 2022 Neuroimaging was performed at the Rotman Research Institute using a Siemens Trio 3.0-Tesla scanner, with slew rate of 400 T/m/s and a 12- channel asymmetric gradient head coil. During the pre- and posttreatment scans, 2 runs of 434 functional volumes were collected, for a total of 868 volumes per assessment.

## Sample size

A total of 85 participants have baseline and post-intervention scans and follow-up data.

## Sample size rationale

The sample size was determined to power detection of functional outcomes in the parent neuroimaging study, but should also apply to the current project (Farb et al., 2022).

## Stopping rule

N/A data has been collected

# Variables

## Manipulated variables

Interventions ----------------------

**Cognitive Behavior Therapy with a Well-Being focus (WB-CT)**

Conducted across 8 weekly, 2 hr long group sessions. Therapists utilized goal setting, self-monitoring, thought records, and cognitive restructuring to address symptoms of anxiety and irritability in Sessions 1– 4, to support lifestyle modification related to stress, interpersonal friction, and fatigue in Sessions 5 and 6 and, in Sessions 7 and 8, to enhance well-being by addressing environmental mastery, life purpose, self-acceptance, and optimizing positive interpersonal relationships in participant’s everyday lives.

**Mindfulness-Based Cognitive Therapy (MBCT)**

Conducted as described in Segal et al. (2013), across 8 weekly group meetings of 2 hr duration and a retreat day held between Sessions 6 and 7. In Sessions 1– 4 of MBCT, the practice of mindfulness meditation was tied to the development of attentional capacities for disengaging from habitual, relapse-related ruminative thought patterns, as well as increasing awareness of everyday activities. Sessions 5 and 6 were devoted to affect regulation, especially the investigation of negative emotional and physical experiences through curiosity and approach rather than avoidance. The final two MBCT sessions addressed developing an “action plan” for responding to early warning signs of relapse/ recurrence.

No files selected

## Measured variables

Self-reference networks

----------------------

Brain activation in dmPFC

Brain Activation in ACC

Functional connectivity in the dorsal nexus

Future relapse:

----------------------

Bivariate measure (yes/no)

Clinical Covariates:

----------------------

Residual Symptoms

Past Episodes

Antidepressant Medication Status (ADM)

Interoceptive dysfunction (i.e., Somatic Anxiety, Unaware) Decentering

No files selected

## Indices

Self-Referential Features:

----------------------

Endorsement: (yes vs. no)

Endorsement bias : frequency of endorsed negative self words - frequency of endorsed positive self words

Memory for Self-Referential Words: (old vs. new judgement)

Recognition Memory: was calculated using the d-prime statistic (discriminability) = proportion of “old” trials (in the relevant category) that participant accurately identified as “old” (hit rate) – proportion of “new” trials that participant inaccurately identified as “old” (false alarm rate). ( See pre-registration Livingston et al., 2019 https://osf.io/bh92n)

Task Factors:

----------------------

Valence (Positive vs. negative)

Task condition (Self vs. Other vs. Case)

Self-referential brain activity:

----------------------

The contrast of BOLD response between [self-endorsed negative words] - [self-endorsed positive words]

Residual symptoms & Decentering

----------------------

Residual symptoms and decentering scores were defined using the composite score described in Segal et al., 2019, Journal of Clinical and Consulting Psychology

Interoceptive dysfunction

----------------------

Interoceptive dysfunction (i.e., Somatic Anxiety and Unaware) were defined using the composite score described in Wu et al., 2024, manuscript submitted for publication

Anti-depression medication

----------------------

History of anti-depression medication over the study period (yes vs. no)

Past depression episodes

----------------------

Number of past depression episodes

Future relapse

----------------------

Depressive relapse will be assessed by dividing participants into two groups: participants who met the criteria for at least one MDD episode (relapse) and participants with no subsequent episodes (no relapse) during the two-year follow-up (see Farb et al., 2018 for more details).

Characterizing Brain Networks

----------------------

A seed-based correlation approach will be used. Bilateral spheres of 5 mm radius will be used as seeds for each network: 1) default mode network (DMN), using a posterior cingulate cortex (PCC) seed (Sridharan et al., 2008); 2) the salience network (SN) using an anterior insula seed (Toga, 2015).

We will specifically investigate the prefrontal regions identified by this network using the Harvard/Oxford atlas to mask only the prefrontal cortex.

For each participant:

Create seed regions of interest (ROIs) for the DMN and SN using established coordinates

Extract timecourse signal from these ROIs and use the ROIs as predictors of whole brain activity to identify the participant specific networks (DMN and SN)

Mask the networks with an anatomical atlas prefrontal cortex (e.g., Harvard Oxford Atlas) to focus on prefrontal aspects of the networks

The result is a separate, participant-specific ROI for the DMN and SN

To characterize the 3) dorsal nexus, a seed-based correlation will be used to compute the connectivity between the prefrontal ROIs for the DMN and SN.

No files selected

# Analysis Plan

## Statistical models

Obs. To account that some predictors vary across time points, a change score (post-treatment - baseline) for the variables : endorsement bias, recognition memory, residual symptoms, SomAnx, Unaware, and Decentering will be used in the model. This will enable to investigate whether changes in these predictors are associated with the hazard of relapse.

H1. Endorsement. We expect that greater endorsement of negative words compared to positive words as a function of self will be associated with future relapse while controlling for residual symptoms, anti-depression medication, past depression episodes, interoceptive dysfunction, and decentering.

A Cox Proportional Hazards Model will be performed in R using the following model structure :

Dependent Variable:

DaysWell: time in the study until relapse or opt-out.

Relapse Status: (1 = relapse, 0 = censored/opted-out).

Predictors:

Endorsement bias (self- condition) : Continuous variable representing the endorsement rate of negative versus positive words, captured for the self task condition

Control Variables:

Residual symptoms

Antidepressant medication

Past depression episodes

SomAnx

Unaware

Decentering

Example:

Model_1 <- coxph(Surv(DaysWell, Relapse) ~ Endorsement_Bias_Change + Residual_Symptoms_Change + Antidepressant_Medication + Past_Depression_Episodes + SomAnx + Unaware + Decentering_Change, data = endorsement_data)

H2. Memory. We expect that better Recognition memory for endorsed negative words than endorsed positive words for self will be associated with future relapse while controlling for residual symptoms, anti-depression medication, past depression episodes, interoceptive dysfunction, and decentering

A Cox Proportional Hazards Model will be performed in R using the following model structure :

Model Structure

Dependent Variable:

DaysWell: time in the study until relapse or opt-out.

Relapse Status: (1 = relapse, 0 = censored/opted-out).

Predictor:

Recognition Memory Bias (Self Condition Only): A continuous variable calculated using the d-prime statistic (discriminability) to measure recognition accuracy, specifically: d-prime (Negative Words) −d-prime (Positive Words)

Control Variables:

Residual symptoms

Antidepressant medication

Past depression episodes

SomAnx

Unaware

Decentering

Example:

Model_2<- coxph (Surv (DaysWell, Relapse) ~ Recognition_Memory_Bias_Change + Residual_Symptoms_Change + SomAnx_Change + Unaware_Change + Decentering_Change + Antidepressant_Medication + Past_Depression_Episodes, data = memory_data)

H3. Neural activity- when comparing self-endorsed negative words to self-endorsed positive words, and while controlling for residual symptoms, anti-depression medication, past depression episodes, interoceptive dysfunction, and decentering:

Self-referential network (DMN). We expect greater activity in the frontal aspect of the DMN

(i.e., the dmPFC) in relapsers compared to non-relapsers.

Dorsal salience network (SN). We expect greater activity in the frontal aspect of the SN

(i.e., the ACC) in relapsers compared to non-relapsers.

Dorsal nexus. We expect more positive (or less negative) functional connectivity between the

prefrontal DMN and SN regions in relapsers compared to non-relapsers.

fMRI data was preprocessed using fmriprep (https://fmriprep.org/en/stable/), including standard steps such as motion correction, slice timing correction, spatial normalization, and smoothing.

Characterization of brain networks (DMN, SN, and dorsal dexus) have been aforementioned under indices

Characterizing Dysphoric Self-Referential Neural Activity

----------------------------------------------------------------------------------------

We define dysphoric self-referential neural activity as [Self Negative Word evaluation > Self Positive Word evaluation].

We will characterize our regions of interest using the combination of a priori anatomical masks and within-dataset functional masks.

For the a priori anatomical masks, we will examine the dmPFC (Superior Frontal Gyrus + Paracingulate Gyrus) and the ACC (Cingulate Gyrus [anterior division]) from the Harvard-Oxford Atlas.

For the within dataset mask, we will isolate which brain regions are responsive to self-evaluation by contrasting BOLD responses associated with the [self > other + case] conditions. To maximize mask inclusiveness, this contrast will be run at p < .001 with no other correction. The dmPFC and the ACC will both be constrained by their intersection with the [Self > Other + Case] mask.

Next, to isolate self-endorsed negative words and self-endorsed positive words, only

trials where the participant responded "yes" to negative words and positive words (respectively) in the self condition will be included.

Subsequently, we will calculate the difference in BOLD response to create a contrast

map of [Self-Endorsed Negative words > Self-Endorsed Positive words]

Finally to extract ROI-specific contrast estimate, we will overlay the generated ROIs (

i.e., dmPFC and ACC) onto the contrast map for each participant, allowing the extraction of participant specific values

Therefore for each participant at each timepoint, we will identify 2 participant-specific ROIs that are more responsive to Self-Endorsed Negative words than Self-Endorsed Positive words- the first ROI will be constrained to the dmPFC anatomical mask, and the second ROI will be constrained to the ACC anatomical mask.

Both masks will be used to small volume correct for multiple comparisons in finding regions that are significantly more active for Self-Endorsed Negative > Self-Endorsed Positive at a corrected P < .05. The median value from all significant voxels within each mask will be used for that participant-specific brain activity score (DMN for H3A and SN for H3B).

Functional Connectivity Analysis for the Dorsal Nexus

-----------------------------------------------------------------------

For each participant, the correlation between the timecourses extracted from the dmPFC aspects of the DMN and the ACC aspects of the SN ROIs will be calculated, where the resultant connectivity value will represent the dorsal nexus connectivity between DMN and SN.

Statistical Analysis

----------------------

Obs. Similar to H1 and H2, to account that some predictors vary across time points, a change score (post-treatment - baseline) for the variables : dmPFC activity, SN_activity, dorsal nexus_activity residual symptoms, SomAnx, Unaware, and Decentering will be used in the model.

For H3A) Self-referential network (DMN). We expect greater activity in the frontal aspect of the DMN (i.e., the dmPFC) in relapsers compared to non-relapsers

A Cox Proportional Hazards Model will be performed in R using the following model structure :

Model Structure

Dependent Variable:

DaysWell: time in the study until relapse or opt-out.

Relapse Status: (1 = relapse, 0 = censored/opted-out).

Predictor:

dmPFC Activity (Self-Referential): Continuous variable representing the contrast

estimate for Self-Endorsed Negative vs. Self-Endorsed Positive in the dmPFC.

Control Variables:

Residual symptoms

Antidepressant medication

Past depression episodes

SomAnx

Unaware

Decentering

Example:

Model_ DMN <- coxph (Surv (DaysWell, Relapse) ~ dmPFC_activity_change + Residual_Symptoms_Change + SomAnx_Change + Unaware_Change + Decentering_Change + Antidepressant_Medication + Past_Depression_Episodes, data = neural_data)

For H3B) Dorsal salience network (SN). We expect greater activity in the frontal aspect of the SN (i.e., the ACC) in relapsers compared to non-relapsers.

A Cox Proportional Hazards Model will be performed in R using the following model structure :

Model Structure

Dependent Variable:

DaysWell: time in the study until relapse or opt-out.

Relapse Status: (1 = relapse, 0 = censored/opted-out).

Predictor:

ACC Activity (Self-Referential): Continuous variable representing the contrast estimate

for Self-Endorsed Negative vs. Self-Endorsed Positive in the ACC.

Control Variables:

Residual symptoms

Antidepressant medication

Past depression episodes

SomAnx

Unaware

Decentering

Example:

Model_ SN <- coxph (Surv (DaysWell, Relapse) ~ acc_activity_change + Residual_Symptoms_Change + SomAnx_Change + Unaware_Change + Decentering_Change + Antidepressant_Medication + Past_Depression_Episodes, data = neural_data)

For H3C) Dorsal nexus. We expect more positive (or less negative) functional connectivity between the prefrontal DMN and SN regions in relapsers compared to non-relapsers.

A Cox Proportional Hazards Model will be performed in R using the following model structure :

Model Structure

Dependent Variable:

DaysWell: time in the study until relapse or opt-out.

Relapse Status: (1 = relapse, 0 = censored/opted-out).

Predictors:

Dorsal nexus connectivity: correlation between the prefrontal DMN (dmPFC) and prefrontal SN (ACC) for each participant.

Control Variables:

Residual symptoms

Antidepressant medication

Past depression episodes

SomAnx

Unaware

Decentering

Example:

Model_ dorsal_nexus <-coxph (Surv (DaysWell, Relapse) ~ dorsal_nexus_activity_change + Residual_Symptoms_Change + SomAnx_Change + Unaware_Change + Decentering_Change + Antidepressant_Medication + Past_Depression_Episodes, data = neural_data)

No files selected

### Transformations

To address potential response biases or systematic misunderstandings of the task, participants with less than 25% accuracy in the following tasks were recoded in the opposite direction:

For the endorsement task, data for any participant whose accuracy on case trials is less than 25% were recoded in the opposite direction (yes becomes no, and no becomes yes).

For the recognition memory task, data for any participant whose overall accuracy is less than 25% were recoded in the opposite direction (old becomes new, and new becomes old).

### Inference criteria

Statistical significance will be set at alpha level of p < .05, accounting for multiple comparisons using the Holm-Bonferonni correction

### Data exclusion

Data exclusion has already been conducted at level of the clinical trial, as has been previously described ( Farb et al., 2022). At present we believe that the sample has complete functional and psychometric data (N = 85 x 2 time points).

Data exclusion from SRET behavioral task:

Participants who:

Do not complete both runs of the self-referential encoding task.

Fail to respond on a certain number of self-encoding task trials (3 standard deviations

below the group mean of completed task trials).

Have an overall mean reaction time on the self-encoding task that falls below a threshold

(3 standard deviations below the group mean reaction time).

Have an accuracy percentage that falls below a threshold (3 standard deviations below

the group mean accuracy) for case trials during the self-encoding task.

Have a variance of zero for responses in any condition (self / other/ case) of the self-

encoding task.

Exclusion for recognition data for participants who:

Do not complete the recognition memory task or for whom there is no recognition

memory data.

Fail to respond on a certain number of recognition memory task trials (3 standard

deviations below the group mean of completed task trials).

Have an overall recognition accuracy that falls below a threshold (3 standard deviations

below the group mean overall recognition accuracy).

Have a variance of zero across all responses in the recognition memory task.

In case a participant completed a run of either the self-encoding task or the recognition memory task multiple times (e.g., for technical reasons), the most recent run will be included in the analysis.

### Missing data

Should we discover participants with missing data, ther data will be excluded from the analysis.

### Exploratory analysis

E1) Residual Symptoms

Endorsement. We expect that residual symptoms will be associated with greater

endorsement of negative words compared to positive words as a function of self

Memory. We expect that residual symptoms will predict better Recognition memory for

endorsed negative words than endorsed positive words for self

Neural activity - when comparing self-endorsed negative words to self-endorsed positive

words

Self-referential network (DMN). We expect that residual symptoms will be

associated with greater activity in the frontal aspect of the DMN (i.e., the dmPFC)

Dorsal salience network (SN). We expect that residual symptoms will be

associated with greater activity in the frontal aspect of the SN (i.e., the dACC) in relapsers compared to non-relapsers.

Dorsal nexus. We expect that residual symptoms will be associated with more

positive (or less negative) functional connectivity between the prefrontal DMN and SN regions.

E1A) A multilevel linear model will be performed in R using the following model structure:

Dependent Variable: Endorsement bias (self- condition) : Continuous variable representing the

endorsement rate of negative versus positive words, captured for the self task condition

Level 1 (Within-subject - 2 time points for each subject):

Residual symptoms

Level 2 (Between-subject)

(Subject ID)

Example:

Model_residuals_endor<- glmer( Endorsement_Bias ~ Residual_Symptoms + (1 | Subject_ID), data = endorsement_data)

E1B) We will use a multilevel linear model where recognition memory is the dependent variable.

Dependent Variable: Recognition Memory Bias (Self Condition Only): A continuous variable

calculated using the d-prime statistic (discriminability) to measure recognition accuracy, specifically:

Recognition Memory Bias = d-prime (Negative Words) −d-prime (Positive Words)

Level 1 (Within-subject - 2 time points for each subject):

Residual symptoms

Level 2 (Between-subject)

(Subject ID)

Example: Model_residuals_memory<- glmer( Recognition Memory Bias~ Residual_Symptoms + (1 | Subject_ID), data = memory_data)

E1C) The aforementioned fMRI processing analysis be performed.

Self-referential network (DMN). We will use a multilevel linear model where dmPFC activity is

the dependent variable.

Model Structure:

Dependent Variable: dmPFC Activity (DMN) Contrast: Activation difference for self-endorsed

negative vs. positive words.

Level 1 (Within-subject - 2 time points for each subject):

Residual symptoms

Level 2 (Between-subject)

(Subject ID)

Example: Model_ residual_dmn <- glmer(dmPFC_activity ~ Residual_Symptoms + (1 | Subject_ID, data = roi_data)

Dorsal salience network (SN).We will use a multilevel linear model where ACC activity is the

dependent variable.

Model Structure:

Dependent Variable: ACC Activity (SN) Contrast: Activation difference for self-endorsed

negative vs. positive words.

Level 1 (Within-subject - 2 time points for each subject):

Residual symptoms

Level 2 (Between-subject)

(Subject ID)

Example:

Model_ residual_sn <- glmer(ACC_activity ~ Residual_Symptoms + (1 | Subject_ID, data = roi_data)

Dorsal nexus.We will use a multilevel linear model where dorsal nexus functional connectivity is

the dependent variable.

Model Structure:

Dependent Variable: Dorsal nexus connectivity: correlation between the prefrontal DMN

(dmPFC) and prefrontal SN (ACC) for each participant.

Level 1 (Within-subject - 2 time points for each subject):

Residual symptoms

Level 2 (Between-subject)

(Subject ID)

Example:

Model_ residual_anexus <- glmer( Dorsal nexus connectivity ~ Residual_Symptoms +

(1 | Subject_ID, data = roi_data)

E2) Decentering

Endorsement. We expect that decentering will be associated with less endorsement of

negative words compared to positive words as a function of self

Memory. We expect that decentering will predict less Recognition memory for endorsed

negative words than endorsed positive words for self

Neural activity - when comparing self-endorsed negative words to self-endorsed positive

words

Self-referential network (DMN). We expect that decentering will be associated

with less activity in the frontal aspect of the DMN (i.e., the dmPFC)

Dorsal salience network (SN). We expect that decentering will be associated with

less activity in the frontal aspect of the SN (i.e., the dACC) in relapsers compared to non-relapsers.

Dorsal nexus. We expect that decentering will be associated with more negative

(or positive) functional connectivity between the prefrontal DMN and SN regions.

A similar analysis from E1A, E1B, and E1C will be applied to E2A, E2B, and E2C, where decentering will be used as a predictor instead of residual symptoms

E3) Treatment effect

Endorsement: in general we expect treatment to reduce negative word endorsement rates,

and this effect to be weaker for those going on to relapse and with greater residual symptoms, but stronger for those with higher decentering:

We expect a 2-way interaction between time and valence on endorsement rates:

positive words will be endorsed more than negative words specifically in the self condition at Time 2 compared to Time 1

We expect a 3-way interaction between relapse, word valence and time on

endorsement, such that Relapse will be associated with less reduction of negative word endorsement compared to positive word endorsement in the self condition at Time 2 compared to Time 1

We expect a 3-way interaction between residual symptoms at baseline, word

valence and time, such that greater residual symptoms at baseline will be associated with a smaller reduction in negative word endorsement compared to positive word endorsement in the self condition at Time 2 compared to Time 1

We expect a 3-way interaction between decentering at baseline, word valence and

time, such that greater decentering at baseline will be associated with a greater reduction in negative word endorsement compared to positive word endorsement in the self condition at Time 2 compared to Time 1

Memory. in general we expect treatment to reduce recognition memory for negative word, and

this effect to be weaker for those going on to relapse and with greater residual symptoms, but stronger for those with higher decentering:

We expect a 2-way interaction between time and valence on recognition memory:

positive words will be remembered more than negative words at. Time 2 compared to Time 1

We expect a 3-way interaction between relapse, word valence and time on recognition

memory, such that Relapse will be associated with less reduction of recognition memory for negative word compared to recognition memory for positive word at Time 2 compared to Time 1

We expect a 3-way interaction between residual symptoms at baseline, word valence and

time, such that greater residual symptoms at baseline will be associated with a smaller reduction in recognition memory for negative word compared to recognition memory for positive word at Time 2 compared to Time 1

We expect a 3-way interaction between decentering at baseline, word valence and time,

such that greater decentering at baseline will be associated with a greater reduction in recognition memory for negative word compared to recognition memory for positive word at Time 2 compared to Time 1

Neural activity - in general we expect treatment to reduce dysphoric self-referential brain activity

(self negative > self positive) in the default mode network (DMN) and dorsal (SN), and reduce functional connectivity in the dorsal nexus. We expect this effect to be weaker for those going on to relapse and with greater residual symptoms, but stronger for those with higher decentering:

We expect that a reduction of self-referential ROI brain activity (dmPFC and ACC) and

dorsal nexus connectivity over time

We expect that Relapse will be associated with less reduction of dysphoric self-

referential brain activity over time

We expect that residual symptoms will be associated with less reduction of dysphoric

self-referential brain activity over time

We expect that decentering will be associated with greater reduction of dysphoric self-

referential brain activity over time

E3Aa) Endorsement: endorsement rates for positive words > endorsement rates for negative words specifically in the self condition at Time 2 > Time 1

Model Structure

Dependent variable: Endorsement Rate: Continuous variable representing the proportion of

words endorsed in the self-condition

Predictors: Time: two levels (Time 1 = baseline, Time 2 = post-treatment). Valence: two levels

(Positive vs. Negative)

Random effects

Subject ID

Interactions:

Time × Valence

Example Model structure: Model_endorsement_bias_ 1 <- lmer (Endorsement_Rate ~ Time * Valence + (1 |Subject_ID), data = endorsement_data)

E3Ab) Endorsement Relapse will be associated with less reduction of negative word

endorsement compared to positive word endorsement in the self condition at Time 2 compared to Time 1

Model Structure

Dependent Variable

Endorsement Rate: Continuous variable representing the proportion of endorsed words in the self condition

Predictors Time:

Categorical variable with two levels (Time 1 = baseline, Time 2 = post-treatment). Valence: Categorical variable with two levels (Positive vs. Negative).

Relapse Status: Binary variable (1 = relapse, 0 = non-relapse).

Interaction

Three-Way Interaction (Time × Valence × Relapse)

Random Effects

Subject ID

Example Model structure:

Model_endorsement_bias_2 <-lmer(Endorsement_Rate ~ Time * Valence * Relapse + (1 | Participant_ID), data = endorsement_data)

E3Ac) Endorsement greater residual symptoms at baseline will be associated with a smaller reduction in negative word endorsement compared to positive word endorsement in the self condition at Time 2 compared to Time 1

Model Structure

Dependent Variable

Endorsement Rate: Continuous variable representing the proportion of endorsed words in the self-condition

Predictors

Time: Categorical variable with two levels (Time 1 = baseline, Time 2 = post-treatment). Valence: Categorical variable with two levels (Positive vs. Negative).

Residual symptoms: continuous

Interaction

Three-Way Interaction (Time × Valence × Residual symptoms)

Random Effects

Subject ID

Example

Model structure: Model_endorsement_bias_3 <-lmer(Endorsement_Rate ~ Time * Valence * Residuals + (1 | Participant_ID), data = endorsement_data)

E3Ad) Endorsement greater decentering at baseline will be associated with a greater reduction in

negative word endorsement compared to positive word endorsement in the self condition at Time 2 compared to Time 1

Model Structure

Dependent Variable

Endorsement Rate: Continuous variable representing the proportion of endorsed words in the self-condition

Predictors

Time: Categorical variable with two levels (Time 1 = baseline, Time 2 = post-treatment). Valence: Categorical variable with two levels (Positive vs. Negative).

Decentering : continuous

Interaction

Three-Way Interaction (Time × Valence × Decentering)

Random Effects

Subject ID

Example

Model structure: Model_endorsement_bias_4 <-lmer(Endorsement_Rate ~ Time * Valence * Decentering + (1 | Participant_ID), data = endorsement_data)

E3Ba) Memory. Positive words will be remembered more than negative words at Time 2

compared to Time 1

Model Structure

Dependent Variable

Recognition memory score: Continuous variable representing the memory accuracy (e.g., d- prime) for positive and negative words

Predictors

Time: Categorical variable with two levels (Time 1 = baseline, Time 2 = post-

treatment).

Valence: Categorical variable with two levels (Positive vs. Negative).

Interaction

Time × Valence

Random Effects

Subject ID

Example

Model structure: model_recognition_memory_1 <- lmer (Recognition_Memory_Score ~ Time * Valence + (1 | Participant_ID), data = memory_data)

E3Bb) Memory. Relapse will be associated with less reduction of recognition memory for negative word compared to recognition memory for positive word at Time 2 compared to Time 1

Model Structure

Dependent Variable

Recognition memory score: Continuous variable representing the memory accuracy (e.g., d-prime) for positive and negative words

Predictors

Time: Categorical variable with two levels (Time 1 = baseline, Time 2 = post-treatment). Valence: Categorical variable with two levels (Positive vs. Negative).

Relapse Status: Binary variable (1 = relapse, 0 = non-relapse).

Interaction

Three-Way Interaction (Time × Valence × Relapse)

Random Effects

Subject ID

Example

Model structure: Model_memory_2 <-lmer( Recognition_memory_score ~ Time * Valence * Relapse + (1 | Participant_ID), data = memory_data)

E3Bc) Memory. Greater residual symptoms at baseline will be associated with a smaller reduction in recognition memory for negative word compared to recognition memory for positive word at Time 2 compared to Time 1 Model Structure

Dependent Variable

Recognition memory score: Continuous variable representing the memory accuracy (e.g., d- prime) for positive and negative words

Predictors

Time: Categorical variable with two levels (Time 1 = baseline, Time 2 = post-treatment). Valence: Categorical variable with two levels (Positive vs. Negative).

Residual symptoms : continuous variable

Interaction

Three-Way Interaction (Time × Valence × Residual Symptoms)

Random Effects

Subject ID

Example

Model structure: Model_memory_3 <-lmer( Recognition_memory_score ~ Time * Valence * Residual_Symptoms + (1 | Participant_ID), data = memory_data)

E3Bd) Memory. Greater decentering at baseline will be associated with a greater reduction in recognition memory for negative word compared to recognition memory for positive word at Time 2 compared to Time 1

Model Structure

Dependent Variable

Recognition memory score: Continuous variable representing the memory accuracy (e.g., d-prime) for positive and negative words

Predictors

Time: Categorical variable with two levels (Time 1 = baseline, Time 2 = post-treatment). Valence: Categorical variable with two levels (Positive vs. Negative).

Decentering : continuous variable

Interaction

Three-Way Interaction (Time × Valence × Decentering)

Random Effects

Subject ID

Example Model structure:

Model_memory_4 <-lmer( Recognition_memory_score ~ Time * Valence * Decentering + (1 | Participant_ID), data = memory_data)

Neural Activity Longitudinal analysis:

------------------------------------------------------------------

As noted earlier, fMRIPrep was used to preprocess images across both time 1 and time 2. Contrast maps for each time point will be computed for each participant in the defined ROIs (dmPFC and ACC) based on the difference in BOLD response for [Self-Endorsed Negative words > Self-Endorsed Positive words].

Next, change scores for each participant’s ROIs will be obtained by creating difference maps, representing the difference in BOLD activity between Time 2 and Time 1 for each ROI.

Functional Connectivity Longitudinal Analysis

----------------------------------------------------------------------------------------

Functional connectivity between the ROIs (e.g., dmPFC and ACC) will be computed for each participant at Time 1 and Time 2 by calculating the correlation of the BOLD https://cpa.ca/student-research-grants/ between these regions. Next, connectivity change scores will be calculated by subtracting the Time 1 connectivity values from the Time 2 connectivity values.

E3Ca) Neural activity. We expect a reduction of self-referential ROI brain activity (i.e., dmPFC

and ACC) and dorsal nexus connectivity over time

Model Structure

Dependent Variables (change score)

dmPFC Activity change score : Continuous variable representing BOLD signal for the

[Self- Endorsed Negative > Self-Endorsed Positive] contrast in the dmPFC ROI [ Time 2 - Time 1]

ACC Activity change score : Continuous variable representing BOLD signal for the same

contrast in the ACC ROI [ Time 2 - Time 1]

Dorsal Nexus Connectivity change score: Continuous variable representing functional

connectivity (correlation) between the dmPFC and ACC [ Time 2 - Time 1]

Example model:

dmPFC_longitudinal <- lmer(dmPFC_Activity_change ~ 1 , data = neural_data) ACC_longitudinal <- lmer(ACC_Activity_change ~ 1 , data = neural_data) dorsal_nexus_longitudinal <- lmer(Dorsal_Nexus_Connectivity_change ~ 1, data = neural_data)

E3Cb) Neural activity. Relapse will be associated with less reduction of dysphoric self-

referential brain activity over time

Model Structure

Dependent Variables (Separate Models for Each Measure)

dmPFC Activity change score ACC Activity change score

Dorsal Nexus Connectivity change score

Key Predictors

Relapse Status: Binary variable (1 = relapse, 0 = non-relapse).

Example model:

dmPFC_longitudinal_relapse <- lmer(dmPFC_Activity_change ~ Relapse_Status , data = neural_data)

ACC_longitudinal_relapse <- lmer(ACC_Activity_change ~ Relapse_Status , data = neural_data)

dorsal_nexus_longitudinal_relapse <- lmer(Dorsal_Nexus_Connectivity_change ~ Relapse_Status, data = neural_data)

E3Cc) Neural activity. residual symptoms will be associated with less reduction of dysphoric self-referential brain activity over time

Model Structure

Dependent Variables (Separate Models for Each Measure)

dmPFC Activity change score ACC Activity change score

Dorsal Nexus Connectivity change score

Key Predictors Residual Symptoms change score (continuous)

Example model:

dmPFC_longitudinal_residuals <- lmer(dmPFC_Activity_change ~ residuals_change , data = neural_data)

ACC_longitudinal_residuals <- lmer(ACC_Activity_change ~ residuals_change , data = neural_data)

Dorsal_nexus_longitudinal_residuals <- lmer(Dorsal_Nexus_Connectivity_change ~ residuals_change, data = neural_data)

E3Cd) Neural activity. Decentering will be associated with greater reduction of dysphoric self-referential brain activity over time

Model Structure

Dependent Variables (Separate Models for Each Measure)

dmPFC Activity change score ACC Activity change score

Dorsal Nexus Connectivity change score

Key Predictors

Decentering change score (continuous)

Example model:

dmPFC_longitudinal_decentering <- lmer(dmPFC_Activity_change ~ decentering_change , data = neural_data)

ACC_longitudinal_decentering <- lmer(ACC_Activity_change ~ decentering_change , data = neural_data)

Dorsal_nexus_longitudinal_decentering<- lmer(Dorsal_Nexus_Connectivity_change ~ decentering_change, data = neural_data)

## Other

**Other**

All neuroimaging data has been collected and preprocessed using fMRIprep (Esteban et al., 2017). Additionally, the behavioral SRET data has been previously pre-registered and analyzed (https://osf.io/bh92n) . However, no analysis were conducted from the SRET neuroimaging data.
