# Supplementary material for: Functional brain biomarkers of self-referential bias in remitted depressed outpatients: a randomized controlled trial
Source: Neuroimage Clin. 2026 Jun 19;51:104025. doi: 10.1016/j.nicl.2026.104025 (PMC13316189; doi:10.1016/j.nicl.2026.104025)
Supplement: Supplementary file 2 — Supplementary material 2 [file mmc2.docx]

**Supplemental Online Content**

**Methods**

**Table S1.** Variance Inflation Factor (VIF) values for covariates in general linear models predicting negative self-referential bias

**Table S2. Conjunction analysis of self-criticism and self-affirming response tendencies**

**Table S3.** Mixed-effects model analysis of residual symptoms predicting negative self-referential bias

**Table S4. Prediction of residual symptoms by negative self-referential bias**

**Table S5. Linear regression models of baseline and post-treatment negative self-referential bias predicting residual symptoms at discrete follow-up times**

**Table S6.** Mixed-effects model analysis of decenter predicting positive self-referential bias

**Table S7. Prediction of decenter by** positive self-referential bias

**Table S8. Linear regression models of baseline and post-treatment** positive self-referential bias **predicting decenter at discrete follow-up times**

**Table S9.** Mixed-effects model analysis of residual symptoms predicting memory recognition bias

**Table S10.** Linear mixed-effects models examined whether baseline and post-treatment memory recognition bias predicted residual symptoms over the follow-up period, while controlling for concurrent residual symptoms and time (months)

**Table S11. Linear regression models of baseline and post-treatment memory recognition bias predicting residual symptoms at discrete follow-up times**

**Table S12.** Mixed-effects model analysis of dysphoric self-referential activity in the frontal default mode network in relapse and non-relapse patients with Major Depressive Disorder (MDD)

**Table S13.** Mixed-effects model analysis of dysphoric self-referential activity in the frontal salience network in relapse and non-relapse patients with Major Depressive Disorder (MDD)

**Table S14.** Cox **proportional hazards models of dysphoric** self-referential activity in the frontal default mode network and the salience network

**Table S15.** Mixed-effects model analysis of mean functional connectivity between frontal component of the default mode network and the salience network in relapse and non-relapse patients with Major Depression Disorder (MDD).

**Table S16.** Mixed-effects model analysis of mean functional connectivity within the frontal default mode network in relapse and non-relapse patients with Major Depression Disorder (MDD)

**Table S17.** Mixed-effects model analysis of mean functional connectivity within the frontal salience network in relapse and non-relapse patients with Major Depression Disorder (MDD).

**Table S18. Static neural markers of relapse vulnerability**

**Table S19.** Mixed-effects model analysis of mean activation differences in the left subgenual cingulate between relapse and non-relapse patients with Major Depression Disorder (MDD)

**Table S20.** Mixed-effects model analysis of mean activation differences in the right lateral occipital cortex between relapse and non-relapse patients with Major Depression Disorder (MDD)

**Table S21.** Mixed-effects model analysis of mean activation differences in the right postcentral gyrus/ primary somatosensory between relapse and non-relapse patients with Major Depression Disorder (MDD)

**Table S22.** Cox **proportional hazards models of static neural markers predicting relapse**

**Table S23.** Dynamic neural markers of treatment-related non-relapse growth (conjunction analysis)

**Table S24.** Mixed-effects model analysis of mean activation differences in the L. cerebellum between relapse and non-relapse patients with Major Depression Disorder (MDD)

**Table S25.** Mixed-effects model analysis of mean activation differences in the L. supramarginal gyrus between relapse and non-relapse patients with Major Depression Disorder (MDD)

**Table S26.** Mixed-effects model analysis of mean activation differences in the R. precentral gyrus between relapse and non-relapse patients with Major Depression Disorder (MDD)

**Table S27.** Mixed-effects model analysis of mean activation differences in the R. superior temporal gyrus between relapse and non-relapse patients with Major Depression Disorder (MDD)

**Table S28.** Mixed-effects model analysis of mean activation differences in the R. inferior frontal gyrus between relapse and non-relapse patients with Major Depression Disorder (MDD)

**Table S29.** Mixed-effects model analysis of mean activation differences in the R. lateral occipital cortex between relapse and non-relapse patients with Major Depression Disorder (MDD)

**Table S30.** Cox **proportional hazards models of dynamic treatment neural markers predicting relapse.**

**Table S31.** Combined Cox regression model of relapse risk including static and dynamic neural markers

**Table S32. Psychophysiological interaction (PPI) analysis: main effects and predictors of relapse**

**Figure S1. Study consort diagram.**

**Figure S2. Mixed-effects models of residual symptoms predicting negative self-referential bias**

**Figure S3.** ROC curves of baseline brain activation predicting relapse status

**Figure S4.** ROC curves of dynamic risk markers associated with treatment response

**Figure S5**. Correlation analysis of the frontal default mode network and salience network dysphoric activity across self-referential contrasts ( Self>Case vs. Self>Other+Case)

**References**

This supplemental material has been provided by the authors to give readers additional information about their work.

**Methods**

**Clinical and Psychometric data**

Participants were remitted depressed outpatients recruited from a broader randomized controlled trial (RCT) of MBCT and WB-CBT(1,2). Before scanning, a licensed psychologist confirmed remission status using the Structured Clinical Interview for DSM (SCID) and the HRSD-17, and participants completed measures of acceptance (AAQ) and rumination (RSQ-R). Following scanning, participants were contacted monthly by staff psychologists to assess time to relapse using the SCID depression module. Symptom assessments occurred bimonthly via the Quick Inventory of Depressive Symptomatology (QIDS), alternating between online (Checkbox eSurvey) and telephone assessments. A QIDS score of 12 triggered a phone assessment with HAMD-17 and SCID. A patient was judged to have an episode of major depression if they scored 16 on HAMD and met criteria on the SCID depression module for that specific interval. Relapse was defined as meeting DSM-IV criteria for a major depressive episode and scoring ≥16 on the HRSD-17; diagnoses were confirmed by an experienced research psychiatrist. Once relapse was identified, participants were re-treated with antidepressant medication within 48 hours. Participants completed the Self-Referential Encoding Task (SRET) during fMRI at baseline and post-treatment, interleaved with a film task(3,4).

Psychometric outcomes were based on 17 self-report measures, comprised of 34 subscales assessing intervention-specific and transdiagnostic constructs linked to relapse vulnerability. A data-driven approach minimized researcher bias and enabled the detection of longitudinal latent structures across domains including negative and positive affect, depressive symptoms, dysfunctional attitudes, stressful life events, mindfulness, acceptance, self-compassion, somatic awareness, emotional intelligence, wellbeing, and life satisfaction. Exploratory factor analysis reduced the 34 scales to three factors identified via Horn’s parallel analysis: **Decentering** (mindfulness, psychological distance, growth orientation), **Distress Tolerance** (low self-judgment, isolation, and rumination), and **Residual Symptoms** (depression, anxiety, sleep difficulties), the latter a well-established prognostic marker following remission (2).

**Sample size, attrition, and retention rate.**

Of 166 participants enrolled in the RCT, 99 (60%) completed the baseline neuroimaging session and 85 (86% of scanned participants) returned for the post-intervention scan. Of the 85 participants entering the two-year follow-up, relapse data were available for 81% at one year and 60% at two years. The neuroimaging subsample did not differ from the full RCT sample on demographic or clinical characteristics at either scan timepoint, and relapse status showed no significant associations with demographics(1,2,5).

**Sample size justification.**

Sample size determination followed the power analysis reported in the parent study(5), which used fMRIPower to estimate the number of participants required to detect mood-related neural responses in prefrontal and sensory regions. Based on those estimates and anticipated attrition, the target enrollment for baseline fMRI was 100 participants.

**Interventions**

As described in Farb et al., 2018, WB-CT followed established protocols (6,7) and was delivered across eight weekly 2-hour group sessions Early sessions targeted anxiety and irritability using goal setting, self-monitoring, thought records, and cognitive restructuring; mid-program sessions emphasized lifestyle modification around stress and interpersonal difficulties; and final sessions focused on enhancing wellbeing through environmental mastery, life purpose, self-acceptance, and positive relationships.

MBCT (Segal et al., 2012) consisted of eight weekly 2-hour sessions plus a retreat day, integrating mindfulness meditation to disengage from ruminative patterns (Sessions 1–4), regulate negative affect through curiosity and approach (Sessions 5–6), and develop individualized relapse-prevention action plans (Sessions 7–8).

**fMRI Acquisition & Processing**

fMRI acquisition and preprocessing followed the protocol reported in Farb et al. (2022). For MRI scanner acquisition parameters, the 3D magnetization-prepared rapid acquisition gradient echo planar pulse sequence was used to construct T1-weighted structural brain images (TR = 2000 ms; TE = 2.63 ms; matrix = 256 x 160; field of view = 256 x 256; slice thickness = 1 mm thick; 160 oblique axial slices; total acquisition time = 6.5 min). Functional images were constructed from the blood oxygenation level-dependent (BOLD) fMRI signal using T2*-weighted gradient-echo echo-planar image pulse sequences (TR = 2000 ms; TE = 2.63 ms; flip angle = 270 degrees; acquisition matrix = 64 x 64; field of view = 200 mm; voxel resolution = 3.1 x 3.1 x 5 mm; 30 slices in oblique axial orientation). Scans were collected at the Rotman Research Institute on a Siemens Trio 3.0T scanner (12-channel asymmetric gradient head coil; 400 T/m/s slew rate). During the pre- and post-treatment scans, 2 runs of 434 functional volumes were collected, for a total of 868 volumes per assessment

Preprocessing for anatomical and functional data were performed with fMRIPrep 20.0.6 (8,9). Anatomical T1-weighted (T1w) images from both baseline and post-intervention were corrected for intensity non-uniformity (INU) with N4BiasFieldCorrection (10) distributed with ANTs 2.2.0 (11). The T1w-reference was then skull-stripped with a Nipype implementation of the antsBrainExtraction.sh workflow (from ANTs), using OASIS30ANTs as target template. Brain tissue segmentation of cerebrospinal fluid (CSF), white-matter (WM) and gray-matter (GM) was performed on the brain-extracted T1w using fast (FSL 5.0.9, RRID:SCR_002823, (12) . A T1w-reference map was computed after registration of 3 T1w images (after INU-correction) using mri_robust_template (FreeSurfer 6.0.1(13). Brain surfaces were reconstructed using recon-all (FreeSurfer 6.0.1, RRID:SCR_001847, (14) , and the brain mask estimated previously was refined with a custom variation of the method to reconcile ANTs-derived and FreeSurfer-derived segmentations of the cortical gray-matter of Mindboggle (RRID:SCR_002438)(15). Volume-based spatial normalization to one standard space (MNI152NLin2009cAsym) was performed through nonlinear registration with antsRegistration (ANTs 2.2.0), using brain-extracted versions of both T1w reference and the T1w template. The following template was selected for spatial normalization: ICBM 152 Nonlinear Asymmetrical template version 2009c (RRID:SCR_008796; TemplateFlow ID: MNI152NLin2009cAsym) (16).

Functional data preprocessing pipeline was performed for each of the BOLD runs found per subject (across all tasks and sessions), with a total of 868 functional volumes for each assessment across two runs. First, a reference volume and its skull-stripped version were generated using a custom methodology of fMRIPrep. Susceptibility distortion correction (SDC) was omitted. The BOLD reference was then co-registered to the T1w reference using bbregister (FreeSurfer) which implements boundary-based registration (17) . Co-registration was configured with six degrees of freedom. Head-motion parameters with respect to the BOLD reference (transformation matrices, and six corresponding rotation and translation parameters) are estimated before any spatiotemporal filtering using mcflirt (FSL 5.0.9, (18). BOLD runs were slice-time corrected using 3dTshift from AFNI 20160207 (RRID:SCR_005927)(19). The BOLD time-series (including slice-timing correction when applied) were resampled onto their original, native space by applying the transforms to correct for head-motion. These resampled BOLD time-series will be referred to as preprocessed BOLD in original space, or just preprocessed BOLD. The BOLD time-series were resampled into standard space, generating a preprocessed BOLD run in MNI152NLin2009cAsym space. First, a reference volume and its skull-stripped version were generated using a custom methodology of fMRIPrep. Several confounding time-series were calculated based on the preprocessed BOLD: framewise displacement (FD), DVARS and three region-wise global signals. FD and DVARS are calculated for each functional run, both using their implementations in Nipype (following the definitions by (20). The three global signals are extracted within the CSF, the WM, and the whole-brain masks. Additionally, a set of physiological regressors were extracted to allow for component-based noise correction (CompCor, (21). Principal components are estimated after high-pass filtering the preprocessed BOLD time-series (using a discrete cosine filter with 128s cut-off) for the two CompCor variants: temporal (tCompCor) and anatomical (aCompCor). tCompCor components are then calculated from the top 5% variable voxels within a mask covering the subcortical regions. This subcortical mask is obtained by heavily eroding the brain mask, which ensures it does not include cortical GM regions. For aCompCor, components are calculated within the intersection of the aforementioned mask and the union of CSF and WM masks calculated in T1w space, after their projection to the native space of each functional run (using the inverse BOLD-to-T1w transformation). Components are also calculated separately within the WM and CSF masks. For each CompCor decomposition, the k components with the largest singular values are retained, such that the retained components’ time series are sufficient to explain 50 percent of variance across the nuisance mask (CSF, WM, combined, or temporal). The remaining components are dropped from consideration. The head-motion estimates calculated in the correction step were also placed within the corresponding confounds file. The confound time series derived from head motion estimates and global signals were expanded with the inclusion of temporal derivatives and quadratic terms for each (22). Frames that exceeded a threshold of 0.5 mm FD or 1.5 standardised DVARS were annotated as motion outliers. All resamplings can be performed with a single interpolation step by composing all the pertinent transformations (i.e. head-motion transform matrices, susceptibility distortion correction when available, and co-registrations to anatomical and output spaces). Gridded (volumetric) resamplings were performed using antsApplyTransforms (ANTs), configured with Lanczos interpolation to minimize the smoothing effects of other kernels (23) Non-gridded (surface) resamplings were performed using mri_vol2surf (FreeSurfer).

**Analysis-specific preprocessing.** For the present task-based analyses, preprocessed BOLD images were entered into first-level SPM12 models. Functional images were spatially smoothed with an 8 mm full-width at half-maximum (FWHM) Gaussian kernel to optimize group-level inference (24). A high-pass filter of 180 s was applied at the first level. Nuisance regressors included 6 motion parameters (trans_x, trans_y, trans_z, rot_x, rot_y, rot_z) , their temporal derivatives (trans_x_derivative1, trans_y_derivative1, trans_z_derivative1, rot_x_derivative1, rot_y_derivative1, rot_z_derivative1)

, CSF signal, framewise displacement, and 6 anatomical CompCor components (a_comp_cor_00 to a_comp_cor_05). Motion outlier frames were identified during preprocessing using thresholds of FD > 0.5 mm or standardized DVARS > 1.5. Although fMRIPrep generated additional nuisance estimates including white matter signal, white matter was not included as a separate regressor in the final first-level models because nuisance variance from white matter and CSF compartments was already partially captured through the aCompCor components (21).

**Deviations from pre-registrations**

Several preregistered steps were modified in this study. First, the preregistered contrast **[Self-endorsed Negative > Self-endorsed Positive]** could not be estimated reliably because of insufficient endorsed trials per condition. We therefore removed the endorsement filter and operationalised the contrast by using all self-negative and self-positive trials, computing the contrast **[Self-Negative > Self-Positive]**. Secondly, we conducted an exploratory sensitivity analysis comparing variations of self-reference DMN and SN functional masks (i.e., Self> Case vs. Self > Other + Case). ROI estimates showed near-perfect convergence across masks (DMN: r = 0.99; ACC: r = 0.99; Figure 4). Therefore, subsequent analysis examining whole-brain **static** and **dynamic** neural markers of relapse vulnerability defined self-referential processing as Self>Case to maximize simplicity and reproducibility of the pipeline. Third, although we originally planned to create participant-specific ROIs by intersecting anatomical frontal DMN (i.e., dorsal medial prefrontal cortex; dmPFC) and frontal SN (i.e., anterior cingulate cortex; ACC ) regions with functional **self-reference** masks, ROIs were instead defined using a priori anatomical masks from the Harvard–Oxford atlas to ensure consistent spatial coverage across participants; median ROI values were extracted for the **Self-Negative – Self-Positive** contrast. This adjustment preserves the conceptual definition of dysphoric self-referential activity while improving robustness. Finally, interoceptive dysfunction (somatic anxiety and body unawareness) was preregistered as a covariate, but these constructs load onto the residual symptoms factor in our validated framework and were not included separately to avoid redundancy.

**Table S1.** Variance Inflation Factor (VIF) values for covariates in general linear models predicting negative self-referential bias

| Predictor | VIF | Tolerance |
| --- | --- | --- |
| Age | 1.13 | 0.88 |
| Gender | 1.05 | 0.94 |
| Past Episodes | 1.05 | 0.95 |
| Symptoms | 1.34 | 0.75 |
| Relapse | 1.49 | 0.67 |
| Antidepressant medication | 1.04 | 0.96 |
| Decenter | 1.23 | 0.81 |
| Days well | 1.43 | 0.70 |

**Note.** VIF values are below the commonly accepted threshold of 5, tolerance values are greater than 0.2, indicating that multicollinearity is not a concern.

**Table S2. Conjunction analysis of self-criticism and self-affirming response tendencies.**

|  |  |  |  | Cluster size |  | MNI coordinates (mm) | | |
| --- | --- | --- | --- | --- | --- | --- | --- | --- |
| *Description* | *Region* | *Side* | *p (FDR)* | *k* | *z-score* | *x* | *y* | *z* |
| **Self-criticism** response | DLPFC | L | 0 | 12358 | 5.24 | -4 | 32 | 32 |
|  | Posterior orbitofrontal cortex/anterior insula | M | 0 | 12786 | 4.88 | 0 | -12 | -12 |
|  | Cerebellum | M | .589 | 1217 | 2.84 | 0 | -50 | -42 |
|  | Posterior cingulate gyrus | L | .896 | 854 | 2.83 | -6 | -46 | 34 |
|  | Lateral occipital cortex | L | .971 | 560 | 2.69 | -66 | -62 | 18 |
| **Self-affirming** response | Postcentral gyrus | R | .245 | 2635 | 3.65 | 50 | -24 | 62 |
|  | Inferior temporal gyrus | R | .971 | 585 | 3.44 | 54 | -50 | -12 |
|  | Posterior cingulate/precuneus | L | .971 | 953 | 3.22 | -28 | -40 | 8 |
|  | Lingual gyrus | R | .971 | 633 | 3.11 | 20 | -62 | -6 |
|  | Superior parietal lobule | L | .971 | 874 | 3.03 | -12 | -56 | 70 |
|  | Posterior supramarginal gyrus | R | .971 | 695 | 2.72 | 58 | -42 | 34 |
| Positive endorsement (up) | Postcentral gyrus | R | 0 | 2450 | 5 | 50 | -28 | 64 |
|  | Superior parietal lobe | L | .013 | 424 | 4.58 | -16 | -58 | 74 |
|  | Lingual gyrus / Temporal occipital fusiform gyrus | R | 0 | 1207 | 4.43 | 26 | -54 | -14 |
|  | Inferior temporal gyrus | R | .013 | 456 | 4.36 | 52 | -48 | -8 |
|  | Insula | R | .005 | 586 | 4.29 | 40 | -10 | -2 |
|  | Somatosensory/ supramarginal gyrus | L | .013 | 417 | 4.25 | -62 | -32 | 38 |
|  | Lateral occipital cortex | R | 0 | 1254 | 4.22 | 26 | -68 | 52 |
|  | Middle temporal gyrus | L | .002 | 711 | 3.93 | -52 | -62 | 0 |
|  | Insula | L | .013 | 426 | 3.5 | -44 | -6 | -2 |
| Positive endorsement (down) | DLPFC | L | 0 | 16855 | 7.16 | -2 | 30 | 42 |
|  | Insula | R | 0 | 2163 | 5.47 | 30 | 20 | -6 |
|  | Posterior cingulate gyrus | L | .003 | 652 | 4.88 | -14 | -54 | 30 |
|  | Lingual gyrus | L | .017 | 422 | 4.06 | -40 | -52 | -44 |
|  | Middle frontal gyrus | L | .008 | 522 | 4.03 | -42 | 16 | 36 |
| Negative endorsement (up) | Orbitofrontal cortex / insula | L | 0 | 6376 | 5.88 | -40 | 26 | -4 |
|  | Anterior cingulate gyrus | L | 0 | 6453 | 5.34 | -6 | 30 | 28 |
|  | Lingual gyrus | R | 0 | 844 | 4.18 | 38 | -70 | -42 |
|  | Orbitofrontal cortex / insula | R | 0 | 980 | 3.95 | 54 | 30 | 2 |
| Negative endorsement (down) | Supramarginal gyrus | R | 0 | 1245 | 4.26 | 60 | -48 | 40 |

**Note.** A mixed 2 (time: wave 1, wave 2) × 3 (condition: self, other, case) × 2 (valence: positive, negative) factorial model was estimated, with positive and negative endorsement included as covariates. The self-cricitism response contrast was defined as the conjunction of positive endorsement decrease and negative endorsement increase, whereas self-affirming response contrast was defined as the conjunction of positive endorsement increase and negative endorsement decrease. Significant activations were observed in prefrontal default mode network and salience network for self-criticism response among patients with Major Depression Disorder (MDD), including the left posterior orbitofrontal cortex/anterior insula and the left dorsal lateral prefrontal cortex (DLPFC). In contrast, self-affirming response was associated with posterior somatosensory activations, including the right postcentral gyrus, inferior temporal gyrus, lingual gyrus and posterior supramarginal gyrus, as well as the left posterior cingulate/precuneus and superior parietal lobule. Conjunction results were thresholded at p < √0.005 (equivalent to conjoint probability control across contrasts), with a cluster extent of k = 400 voxels. Positive and Negative endorsement contrast results were thresholded at p < .005, cluster size k = 400.

**Table S3.** Mixed-effects model analysis of residual symptoms predicting negative self-referential bias.

|  | Residual symptoms effect | | | | Residual symptoms and Time effect | | | | | Residual symptoms x Time interaction | | | | Residual symptoms at Time 1 | | | | Residual symptoms at Time 2 | | | | Residual symptoms and Covariates effect | | | |
| --- | --- | --- | --- | --- | --- | --- | --- | --- | --- | --- | --- | --- | --- | --- | --- | --- | --- | --- | --- | --- | --- | --- | --- | --- | --- |
| *Predictors* | *Estimates* | *CI* | *p* | *Estimates* | | | *CI* | *p* | *Estimates* | | *CI* | *p* | *Estimates* | | *CI* | *p* | *Estimates* | | *CI* | *p* | *Estimates* | | *CI* | *p* |  |
| (Intercept) | .26 | 0.19; 0.33 | **<.001** | .39 | | .28; .49 | | **<.001** | .42 | | .27; .56 | **<.0001** | .31 | | .23; .40 | **<.001** | .21 | | .13; .29 | **<.001** | .44 | | .21; .66 | **<.001** |  |
| Residual symptoms | .11 | .07; .15 | **<.001** | .12 | | .08; .15 | | **<.001** | .11 | | .01; .21 | **.027** | .12 | | .06 ; .17 | **<.001** | .14 | | .09; .19 | **<.001** | .10 | | .06; .14 | **<.001** |  |
| ADM (yes) | -.07 | -.16; .02 | .121 | -.07 | | -.16; .02 | | .117 | -.12 | | -.30; .06 | .197 | -.09 | | -.19; .02 | .110 | -.05 | | -.15; .05 | .295 | -.07 | | -.15; .01 | .103 |  |
| Time |  |  |  | -.08 | | -.13; -.03 | | **.001** | -.10 | | -.09; -.02 | **.017** |  | |  |  |  | |  |  | -.07 | | -.12; -.01 | **.012** |  |
| Symptoms x Time |  |  |  |  | |  | |  | .00 | | -.05; .06 | .893 |  | |  |  |  | |  |  |  | |  |  |  |
| ADM (yes) x Time |  |  |  |  | |  | |  | .03 | | -.07; .14 | .547 |  | |  |  |  | |  |  |  | |  |  |  |
| Decenter |  |  |  |  | |  | |  |  | |  |  |  | |  |  |  | |  |  | -.04 | | -.08; -.01 | **.013** |  |
| Episodes |  |  |  |  | |  | |  |  | |  |  |  | |  |  |  | |  |  | -.00 | | -.02; .01 | .821 |  |
| Age |  |  |  |  | |  | |  |  | |  |  |  | |  |  |  | |  |  | -.00 | | -.01; .00 | .062 |  |
| Gender |  |  |  |  | |  | |  |  | |  |  |  | |  |  |  | |  |  | .03 | | -.05; .11 | .484 |  |
| **Random effects** |  |  |  |  | |  | |  |  | |  |  |  | |  |  |  | |  |  |  | |  |  |  |
| σ^2^ | .03 |  |  | .02 | |  | |  | .03 | |  |  |  | |  |  |  | |  |  | .03 | | | |  |
| τ₀₀ | .02_ID_ |  |  | .02_ID_ | |  | |  | .02_ID_ | |  |  |  | |  |  |  | |  |  | .02_ID_ | | | |  |
| ICC | .45 |  |  | .49 | |  | |  | .48 | |  |  |  | |  |  |  | |  |  | .43 | | | |  |
| N | 81_ID_ |  |  | 81_ID_ | |  | |  | 81_ID_ | |  |  |  | |  |  |  | |  |  | 81_ID_ | | | |  |
| Observations | 161 |  |  | 161 | |  | |  | 161 | |  |  | 81 | |  |  | 80 | |  |  | 161 | | | |  |
| Marginal R^2^ / Conditional R^2^ | .185/.551 |  |  | .219/.60 | |  | |  | .220 / .594 | |  |  | .203/.182 | |  |  | .281/.262 | |  |  | .292/.594 | | | |  |

**Note.** Negative self-referential bias was defined as the relative proportion of negative to positive endorsements in the self-condition among patients with Major Depression Disorder (MDD). A main effect of Residual symptoms and Time was significant across analysis. No significant Residual symptoms × Time interaction was found. Model fit improved with the inclusion of covariates, revealing additional main effect of decentering. All analyses were controlled for antidepressant medication (ADM), and self-referential bias scores were winsorized.

**Table S4. Prediction of residual symptoms by negative self-referential bias**

|  | Baseline self-reference and Time effect | | | | Post-treatment self-reference and Time effect | | | | Full model and Covariates effect | | | | |
| --- | --- | --- | --- | --- | --- | --- | --- | --- | --- | --- | --- | --- | --- |
| *Predictors* | *Estimates* | *CI* | *p* | *Estimates* | | *CI* | *p* | *Estimates* | | | *CI* | *p* |  |
| (Intercept) | -.16 | -.42; -.10 | .228 | -.11 | | -.35; .12 | .332 | -.24 | | -.74;.27 | | .364 |  |
| Time (months) | -.01 | -.02; .00 | .**033** | -.01 | | -.02; -.00 | .051 | -.01 | | -.02; .00 | | .122 |  |
| Baseline self-reference | 1.51 | .86; 2.16 | **<.001** |  | |  |  | .58 | | -.02; 1.19 | | .059 |  |
| Post-treatment self-reference |  |  |  | 1.55 | | .95; 2.16 | **<.001** | .91 | | .34; 1.48 | | **.002** |  |
| Previous timepoint residual symptoms |  |  |  |  | |  |  | .21 | | .13;.30 | | **<.001** |  |
| Episodes |  |  |  |  | |  |  | .02 | | -.02; .07 | | .333 |  |
| ADM |  |  |  |  | |  |  | .01 | | -.24; .25 | | .949 |  |
| Age |  |  |  |  | |  |  | -.00 | | -.01; .01 | | .854 |  |
| **Random effects** |  |  |  |  | |  |  |  | | | | |  |
| σ^2^ | .54 |  |  | .52 | |  |  | .55 | | | | |  |
| τ₀₀ | .42_ID_ |  |  | .39_ID_ | |  |  | .18 _ID_ | | | | |  |
| ICC | .44 |  |  | .43 | |  |  | .25 | | | | |  |
| N | 81_ID_ |  |  | 80_ID_ | |  |  | 80 _ID_ | | | | |  |
| Observations | 486 |  |  | 480 | |  |  | 480 | | | | |  |
| Marginal R^2^ / Conditional R^2^ | .124/.509 |  |  | .146 / .512 | |  |  | .234/.427 | | | | |  |

**Note.** Linear mixed-effects models examined whether baseline and post-treatment negative self-referential bias predicted residual symptoms over the follow-up period (4 – 24 months), while controlling for concurrent residual symptoms and time (months)**.** Negative self-referential bias was defined as the relative proportion of negative to positive endorsements in the self condition among patients with Major Depression Disorder (MDD). Both baseline and post-treatment negative self-referential bias were positively associated with higher residual symptoms. However, the full model with covariates effect provided the best fit, with only post-treatment negative self-referential bias remaining significant. Self-referential bias scores were winsorized.

**Table S5. Linear regression models of baseline and post-treatment negative self-referential bias predicting residual symptoms at discrete follow-up times.**

|  | Residual symptoms at Month 2 | | | Residual symptoms at Month 4 | | | | Residual symptoms at Month 8 | | | | Residual symptoms at Month 12 | | | Residual symptoms at Month 16 | | | Residual symptoms at Month 20 | | | | Residual symptoms at Month 24 | | | |
| --- | --- | --- | --- | --- | --- | --- | --- | --- | --- | --- | --- | --- | --- | --- | --- | --- | --- | --- | --- | --- | --- | --- | --- | --- | --- |
| *Predictors* | *Estimates* | *CI* | *p* | *Estimates* | *CI* | *p* | *Estimates* | | *CI* | *p* | *Estimates* | | *CI* | *p* | *Estimates* | *CI* | *p* | *Estimates* | *CI* | | *p* | *Estimates* | *CI* | | *p* |
| (Intercept) | -.09 | -.34; .15 | .45 | -.21 | -.54; .11 | .186 | -.16 | | -.45; .12 | .252 | -.17 | | -.41 ;.07 | .156 | -.23 | -.48; .03 | .081 | -.04 | -.31; .23 | | .761 | -.32 | -.56; -.07 | | **.011** |
| Previous timepoint residual symptoms | .53 | .33; .72 | **<.001** | .59 | .33 ;.85 | **<.001** | .24 | | .06 ;.43 | **.011** | .79 | | .61 ;.97 | **<.001** | .62 | .45; .80 | **<.001** | .47 | .29; .65 | | **<.001** | .44 | .26 ;.63 | | **<.001** |
| Baseline self-reference | -.19 | -1.01; .63 | .652 | 1.75 | .68 ;2.83 | **.002** | -.10 | | -1.12 ;.92 | .847 | .32 | | -.48; 1.12 | .425 | .82 | -.04; 1.69 | .061 | -.25 | -1.16; .66 | | .593 | .10 | -.73 ; .93 | | .807 |
| Post-treatment self-reference | 1.55 | .80; 2.30 | **<.001** | -.24 | -1.36; .88 | .667 | .80 | | -.12; 1.72 | .088 | .56 | | -.22 ;1.35 | .158 | .15 | -.70; 1.01 | .720 | .58 | -.28; 1.45 | | .184 | 1.33 | **.**51 ;2.14 | | **.002** |
| Observations | 80 |  |  | 80 |  |  | 80 | |  |  | 80 | |  |  | 80 |  |  | 80 |  |  | | 80 |  |  | |
| R^2^ / R^2^ Adjusted | .482/.461 |  |  | .380/.355 |  |  | .182/.149 | |  |  | .573/.556 | |  |  | .516/.497 |  |  | .335/.309 |  |  | | .427/.404 |  |  | |

**Note.** Models examined the effects of baseline and post-treatment negative self-referential bias on residual symptoms at 2, 4, 8, 12, 16, 20, and 24 months, while controlling for concurrent residual symptoms. Post-treatment negative self-referential bias was a significant positive predictor at 2 and 24 months, whereas baseline negative self-referential bias predicted higher residual symptoms at 4 months.

**Table S6.** Mixed-effects model analysis of decenter predicting positive self-referential bias

|  | | Decentering effect | | | | | Decentering and Time effect | | | | | Decentering x Time interaction | | | | Decentering at Time 1 | | | | Decentering at Time 2 | | | | Decentering and Covariates effect | | | |
| --- | --- | --- | --- | --- | --- | --- | --- | --- | --- | --- | --- | --- | --- | --- | --- | --- | --- | --- | --- | --- | --- | --- | --- | --- | --- | --- | --- |
| *Predictors* | | *Estimates* | | *CI* | *p* | *Estimates* | | | *CI* | *p* | *Estimates* | | *CI* | *p* | *Estimates* | | *CI* | *p* | *Estimates* | | *CI* | *p* | *Estimates* | | *CI* | *p* |  |
| (Intercept) | 9.16 | | 7.29; 11.03 | | **<.001** | 6.29 | | 3.24; 9.34 | | **<.001** | 5.75 | | 1.31; 10.19 | **.012** | 8.09 | | 5.89; 10.28 | **<.001** | 10.25 | | 7.81; 12.69 | **<.001** | 2.04 | | -4.14; 8.22 | .515 |  |
| Decenter | 2.53 | | 1.58; 3.47 | | **<.001** | 2.32 | | 1.37; .3.28 | | **<.001** | 2.35 | | -.00; 4.70 | .050 | 2.64 | | 1.52 ; 3.76 | **<.001** | 2.62 | | 1.22; 4.02 | **<.001** | 1.62 | | .62; 2.62 | **.002** |  |
| ADM (yes) | 1.74 | | -.59; 4.08 | | .143 | 1.72 | | -.62; 4.07 | | .148 | 2.59 | | -2.91; 8.09 | .354 | 2.04 | | -.68; 4.75 | .139 | 1.57 | | -1.50; 4.64 | .313 | 1.33 | | -.94; 3.60 | .249 |  |
| Time |  | |  | |  | 1.90 | | .30; 3.49 | | **.020** | 2.26 | | -.41; 4.93 | .097 |  | |  |  |  | |  |  | 2.41 | | .82; 3.99 | **.003** |  |
| Decenter x Time |  | |  | |  |  | |  | |  | -.01 | | -1.52; 1.49 | .985 |  | |  |  |  | |  |  |  | |  |  |  |
| ADM (yes) x Time | |  | |  |  |  | |  | |  | -.57 | | -3.90; 2.75 | .734 |  | |  |  |  | |  |  |  | |  |  |  |
| Residual symptoms | |  | |  |  |  | |  | |  |  | |  |  |  | |  |  |  | |  |  | -1.90 | | -3.07; -.07 | **.002** |  |
| Episodes | |  | |  |  |  | |  | |  |  | |  |  |  | |  |  |  | |  |  | -.16 | | -.58; .26 | .453 |  |
| Age | |  | |  |  |  | |  | |  |  | |  |  |  | |  |  |  | |  |  | .10 | | .00; .19 | **.039** |  |
| Gender | |  | |  |  |  | |  | |  |  | |  |  |  | |  |  |  | |  |  | .38 | | -1.93; 2.69 | .748 |  |
| **Random effects** | |  | |  |  |  | |  | |  |  | |  |  |  | |  |  |  | |  |  |  | |  |  |  |
| σ^2^ | | 27.13 | |  |  | 25.53 | |  | |  | 26.13 | |  |  |  | |  |  |  | |  |  | .03 | | | |  |
| τ₀₀ | | 12.36_ID_ | |  |  | 13.34_ID_ | |  | |  | 13.06_ID_ | |  |  |  | |  |  |  | |  |  | .02_ID_ | | | |  |
| ICC | | .31 | |  |  | .34 | |  | |  | .33 | |  |  |  | |  |  |  | |  |  | .43 | | | |  |
| N | | 81_ID_ | |  |  | 81_ID_ | |  | |  | 81_ID_ | |  |  |  | |  |  |  | |  |  | 81_ID_ | | | |  |
| Observations | | 161 | |  |  | 161 | |  | |  | 161 | |  |  | 81 | |  |  | 80 | |  |  | 161 | | | |  |
| Marginal R^2^ / Conditional R^2^ | | .176/.434 | |  |  | .182/.46 | |  | |  | .182 / .45 | |  |  | .233/.213 | |  |  | .158/.136 | |  |  | .292/.594 | | | |  |

**Note.** Positive self-referential bias was defined as the relative proportion of positive to negative endorsements in the self-condition among patients with Major Depression Disorder (MDD). A main effect of Decenter was significant across analysis. No significant Decenter × Time interaction was found. Model fit improved with the inclusion of covariates, revealing additional main effect of Residuals symptoms, Age and Time. All analyses were controlled for antidepressant medication (ADM), and self-referential bias scores were winsorized.

**Table S7. Prediction of decenter by** positive self-referential bias

|  | Baseline self-reference and Time effect | | | | Post-treatment self-reference and Time effect | | | | Full model and Covariates effect | | | | |
| --- | --- | --- | --- | --- | --- | --- | --- | --- | --- | --- | --- | --- | --- |
| *Predictors* | *Estimates* | *CI* | *p* | *Estimates* | | *CI* | *p* | *Estimates* | | | *CI* | *p* |  |
| (Intercept) | -.44 | -.74; -.13 | .**005** | -.56 | | -.90; -.23 | **.001** | -.39 | | -.86;.07 | | .099 |  |
| Time (months) | .01 | .00; .02 | .**003** | .01 | | .00; -.02 | **.005** | .01 | | -.00; .02 | | .077 |  |
| Baseline self-reference | .04 | .01; .06 | **.005** |  | |  |  | .01 | | -.01; .03 | | .301 |  |
| Post-treatment self-reference |  |  |  | .04 | | .02; .07 | **.001** | .02 | | .00; .04 | | **.032** |  |
| Previous timepoint Decenter |  |  |  |  | |  |  | .34 | | .25;.42 | | **<.001** |  |
| Episodes |  |  |  |  | |  |  | -.01 | | -.05; .03 | | .663 |  |
| ADM |  |  |  |  | |  |  | -.03 | | -.27; .20 | | .784 |  |
| Age |  |  |  |  | |  |  | .00 | | -.01; .01 | | .694 |  |
| **Random effects** |  |  |  |  | |  |  |  | | | | |  |
| σ^2^ | .38 |  |  | .52 | |  |  | .41 | | | | |  |
| τ₀₀ | .55_ID_ |  |  | .39_ID_ | |  |  | .19 _ID_ | | | | |  |
| ICC | .59 |  |  | .43 | |  |  | .32 | | | | |  |
| N | 81_ID_ |  |  | 80_ID_ | |  |  | 80 _ID_ | | | | |  |
| Observations | 486 |  |  | 480 | |  |  | 480 | | | | |  |
| Marginal R^2^ / Conditional R^2^ | .069/.622 |  |  | .091 / .625 | |  |  | .258/.496 | | | | |  |

**Note.** Linear mixed-effects models examined whether baseline and post-treatment positive self-referential bias predicted decenter over the follow-up period (4 – 24 months), while controlling for concurrent decenter and time (months)**. P**ositive self-referential bias was defined as the relative proportion of positive to negative endorsements in the self-condition among patients with Major Depression Disorder (MDD). Both baseline and post-treatment positive self-referential bias bias were associated with higher decenter. However, the full model with covariates effect provided the best fit, with only post-treatment positive self-referential bias remaining significant. Self-referential bias scores were winsorized.

**Table S8. Linear regression models of baseline and post-treatment** positive self-referential bias **predicting decenter at discrete follow-up times.**

|  | Decenter at Month 2 | | | Decenter at Month 4 | | | | Decenter at Month 8 | | | | Decenter at Month 12 | | | Decenter at Month 16 | | | Decenter at Month 20 | | | | Decenter at Month 24 | | | |
| --- | --- | --- | --- | --- | --- | --- | --- | --- | --- | --- | --- | --- | --- | --- | --- | --- | --- | --- | --- | --- | --- | --- | --- | --- | --- |
| *Predictors* | *Estimates* | *CI* | *p* | *Estimates* | *CI* | *p* | *Estimates* | | *CI* | *p* | *Estimates* | | *CI* | *p* | *Estimates* | *CI* | *p* | *Estimates* | *CI* | | *p* | *Estimates* | *CI* | | *p* |
| (Intercept) | -.04 | -.43; .34 | .835 | -.42 | -.82; -.02 | .**039** | -.00 | | -.37; .36 | .996 | -.25 | | -.54 ;.05 | .098 | .07 | -.24; .38 | .659 | .05 | -.25; .36 | | .725 | -.13 | -.44; .18 | | .416 |
| Previous timepoint Decenter | .62 | .46; .78 | **<.001** | .60 | .41 ;.80 | **<.001** | .45 | | .28 ;.62 | **<.001** | .82 | | .65 ;.99 | **<.001** | .78 | .63; .94 | **<.001** | .65 | .49; .80 | | **<.001** | .65 | .48 ;.82 | | **<.001** |
| Baseline self-reference | .01 | -.02; .04 | .451 | .02 | -.02 ;.05 | .321 | -.00 | | -.03 ;.03 | .778 | .02 | | -.01; .04 | .222 | -.00 | -.03; .03 | .911 | -.01 | -.04; .02 | | .477 | .00 | -.03 ; .03 | | .970 |
| Post-treatment self-erence | .01 | -.02; .04 | .457 | .01 | -.02; .04 | .431 | .02 | | -.01; .05 | .204 | .01 | | -.02 ;.03 | .463 | .01 | -.02; .03 | .646 | -.00 | -.03; .03 | | .971 | .02 | **-**.01 ;.04 | | .143 |
| Observations | 80 |  |  | 80 |  |  | 80 | |  |  | 80 | |  |  | 80 |  |  | 80 |  |  | | 80 |  |  | |
| R^2^ / R^2^ Adjusted | .560/.543 |  |  | .458/.436 |  |  | .352/.326 | |  |  | .614/.599 | |  |  | .609/.594 |  |  | .493/.473 |  |  | | .474/.453 |  |  | |

**Note.** Models examined the effects of baseline and post-treatment low negative self-referential bias on residual symptoms at 2, 4, 8, 12, 16, 20, and 24 months, while controlling for concurrent decenter scores.

**Table S9.** Mixed-effects model analysis of residual symptoms predicting memory recognition bias.

|  | Residual symptoms effect | | | Residual symptoms and Time effect | | | | Residual symptoms x Time interaction | | | | Residual symptoms at Time 1 | | | Residual symptoms at Time 2 | | | Residual symptoms and Covariates effect | | |
| --- | --- | --- | --- | --- | --- | --- | --- | --- | --- | --- | --- | --- | --- | --- | --- | --- | --- | --- | --- | --- |
| *Predictors* | *Estimates* | *CI* | *p* | *Estimates* | *CI* | *p* | *Estimates* | | *CI* | *p* | *Estimates* | | *CI* | *p* | *Estimates* | *CI* | *p* | *Estimates* | *CI* | *p* |
| (Intercept) | 1.06 | 0.84; 1.28 | **<.001** | 1.23 | .80; 1.67 | **<.001** | 1.49 | | .85; 2.14 | **<.0001** | 1.21 | | .89 ;1.53 | **<.001** | .93 | .65; 1.22 | **<.001** | 1.14 | .32; .197 | **<.001** |
| Residual symptoms | -.09 | -.23; .06 | .240 | -.08 | -.23; .07 | .303 | .09 | | -.37; .56 | .0691 | -.02 | | -.25 ; .22 | .882 | -.12 | -.30; .06 | .193 | -.14 | -.30; .03 | .101 |
| ADM (yes) | .17 | -.12; .45 | .244 | .17 | -.11; .45 | .242 | -.25 | | -1.06; .57 | .546 | .03 | | -.38 ; .43 | .898 | .30 | -.06; .66 | .098 | .17 | -.12; .46 | .252 |
| Time |  |  |  | -.11 | -.36; .14 | .375 | -.28 | | -.68; .12 | .169 |  | |  |  |  |  |  | -.08 | -.33; .18 | .555 |
| Residual symptoms x Time |  |  |  |  |  |  | -.11 | | -.39; .18 | .454 |  | |  |  |  |  |  |  |  |  |
| ADM (yes) x Time |  |  |  |  |  |  | .28 | | -.23; .79 | .287 |  | |  |  |  |  |  |  |  |  |
| Decenter |  |  |  |  |  |  |  | |  |  |  | |  |  |  |  |  | -.11 | -.25; .02 | .106 |
| Episodes |  |  |  |  |  |  |  | |  |  |  | |  |  |  |  |  | -.00 | -.06 ;.05 | .870 |
| Age |  |  |  |  |  |  |  | |  |  |  | |  |  |  |  |  | .00 | -.01; .02 | .504 |
| Gender |  |  |  |  |  |  |  | |  |  |  | |  |  |  |  |  | -.07 | -.39 ;.25 | .660 |
| **Random effects** |  |  |  |  |  |  |  | |  |  |  | |  |  |  |  |  |  |  |  |
| σ^2^ | .51 |  |  | . 51 |  |  | . 51 | |  |  |  | |  |  |  |  |  | .52 | | |
| τ₀₀ | .06_ID_ |  |  | . 06_ID_ |  |  | . 06_ID_ | |  |  |  | |  |  |  |  |  | .06_ID_ | | |
| ICC | .10 |  |  | .10 |  |  | .10 | |  |  |  | |  |  |  |  |  | .10 | | |
| N | 65_ID_ |  |  | 65_ID_ |  |  | 65_ID_ | |  |  |  | |  |  |  |  |  | 65_ID_ | | |
| Observations | 130 |  |  | 130 |  |  | 130 | |  |  | 65 | |  |  | 65 |  |  | 130 | | |
| Marginal R^2^ / Conditional R^2^ | .023/.123 |  |  | .028/.126 |  |  | .04 / .139 | |  |  | .001/-.032 | |  |  | .073/.043 |  |  | .05/.143 | | |

**Note.** Memory recognition bias was defined as the relative tendency to correctly recognize previously endorsed negative versus positive words, calculated as the ratio of negative hit rate to positive hit rate, with higher values indicating a stronger bias toward recognizing negative information. No significant effects were observed. All analyses were controlled for antidepressant medication (ADM), and memory recognition bias scores were winsorized.

**Table S10.** Linear mixed-effects models examined whether baseline and post-treatment memory recognition bias predicted residual symptoms over the follow-up period, while controlling for concurrent residual symptoms and time (months)**.**

|  | Concurrent residual symptoms effect | | | Baseline recognition bias and Time effect | | | | Post-treatment recognition bias and Time effect | | | | Baseline recognition bias, Time effect, and Concurrent residual symptoms effect | | | Post-treatment recognition bias, Time effect, and Concurrent residual symptoms effect | | | Full model | | | Full model and Covariates effects | | |
| --- | --- | --- | --- | --- | --- | --- | --- | --- | --- | --- | --- | --- | --- | --- | --- | --- | --- | --- | --- | --- | --- | --- | --- |
| *Predictors* | *Estimates* | *CI* | *p* | *Estimates* | *CI* | *p* | *Estimates* | | *CI* | *p* | *Estimates* | | *CI* | *p* | *Estimates* | *CI* | *p* | *Estimates* | *CI* | *p* | *Estimates* | *CI* | *P* |
| (Intercept) | .06 | -.13; .26 | .513 | .06 | -.30; .41 | .744 | .14 | | -.21; .49 | .429 | .02 | | -.26 ;.31 | .864 | .07 | -.21; .36 | .607 | .04 | -.30; .38 | .830 | .19 | -.38; .76 | .514 |
| Concurrent residual symptoms | .25 | .15; .34 | **<.001** |  |  |  |  | |  |  | .25 | | .15 ;.34 | **<.001** | .24 | .15; .34 | **<.001** | .24 | .15; .34 | **<.001** | .25 | .15; .35 | **<.001** |
| Time (months) | -.00 | -.01; .001 | .442 | -.01 | -.02; .00 | .198 | -.01 | | -.02; .00 | .198 | -.00 | | -.01; .01 | .442 | -.00 | -.01; .01 | .442 | -.00 | -.01; .01 | .442 | -.00 | -.01; .01 | .448 |
| Baseline recognition bias |  |  |  | .04 | -.18; .27 | .717 |  | |  |  | .03 | | -.14 ;.20 | .710 |  |  |  | .03 | -.14; .21 | .701 | .04 | -.13; .21 | .631 |
| Post-treatment recognition bias |  |  |  |  |  |  | -.03 | | -.27; .22 | .824 |  | |  |  | -.01 | -.20; .18 | .928 | -.01 | -.20; .18 | .896 | .01 | -.18; .20 | .918 |
| Episodes |  |  |  |  |  |  |  | |  |  |  | |  |  |  |  |  |  |  |  | .02 | -.03; .08 | .402 |
| ADM |  |  |  |  |  |  |  | |  |  |  | |  |  |  |  |  |  |  |  | -.17 | -.44; .11 | .227 |
| age |  |  |  |  |  |  |  | |  |  |  | |  |  |  |  |  |  |  |  | -.00 | -.02; .01 | .425 |
| **Random effects** |  |  |  |  |  |  |  | |  |  |  | |  |  |  |  |  |  |  |  |  | | |
| σ^2^ | .47 |  |  | .43 |  |  | .43 | |  |  | .47 | |  |  | .47 |  |  | .47 | | | .47 | | |
| τ₀₀ | .22_ID_ |  |  | .45_ID_ |  |  | .45_ID_ | |  |  | .22_ID_ | |  |  | .22_ID_ |  |  | .22_ID_ | | | .21_ID_ | | |
| ICC | .32 |  |  | .51 |  |  | .51 | |  |  | .32 | |  |  | .32 |  |  | .32 | | | .31 | | |
| N | 65_ID_ |  |  | 65_ID_ |  |  | 65_ID_ | |  |  | 65_ID_ | |  |  | 65_ID_ |  |  | 65_ID_ | | | 65_ID_ | | |
| Observations | 390 |  |  | 390 |  |  | 390 | |  |  | 390 | |  |  | 390 |  |  | 390 | | | 390 | | |
| Marginal R^2^ / Conditional R^2^ | .073/.372 |  |  | .003/.509 |  |  | .003 / .509 | |  |  | .074/.372 | |  |  | .073/.372 |  |  | .074/.373 | | | .101/.375 | | |

**Note.** Memory recognition bias was defined as the relative tendency to correctly recognize previously endorsed negative versus positive words, calculated as the ratio of negative hit rate to positive hit rate, with higher values indicating a stronger bias toward recognizing negative information. No significant recognition bias effects were observed. Memory recognition bias scores were winsorized.

**Table S11. Linear regression models of baseline and post-treatment memory recognition bias predicting residual symptoms at discrete follow-up times.**

|  | Residual symptoms at Month 2 | | | Residual symptoms at Month 4 | | | | Residual symptoms at Month 8 | | | | Residual symptoms at Month 12 | | | Residual symptoms at Month 16 | | | Residual symptoms at Month 20 | | | Residual symptoms at Month 24 | | |
| --- | --- | --- | --- | --- | --- | --- | --- | --- | --- | --- | --- | --- | --- | --- | --- | --- | --- | --- | --- | --- | --- | --- | --- |
| *Predictors* | *Estimates* | *CI* | *p* | *Estimates* | *CI* | *p* | *Estimates* | | *CI* | *p* | *Estimates* | | *CI* | *p* | *Estimates* | *CI* | *p* | *Estimates* | *CI* | *p* | *Estimates* | *CI* | *p* |
| (Intercept) | .35 | -.13; .82 | .150 | -.07 | -.54; .39 | .759 | -.07 | | -.49; .35 | .746 | -.11 | | -.51 ;.29 | .581 | .16 | -.29; .60 | .486 | -.08 | -.52; .35 | .698 | -.06 | -.57; .44 | .800 |
| Concurrent residual symptoms | .59 | .34; .83 | **<.001** | .71 | .50 ;.92 | **<.001** | .36 | | .18 ;.53 | **<.001** | .71 | | .50 ;.93 | **<.001** | .84 | .62; 1.06 | **<.001** | .41 | .23; .59 | **<.001** | .58 | .32 ;.84 | **<.001** |
| Baseline recognition bias | .02 | -.24; .29 | .869 | .12 | -.14 ;.37 | .368 | -.06 | | -.30 ;.17 | .579 | .09 | | -.13; .31 | .430 | .01 | -.24; .25 | .941 | -.03 | -.27; .21 | .817 | .01 | -.27 ;.29 | .938 |
| Post-treatment recognition bias | -.15 | -.44; .14 | .317 | .02 | -.27; .30 | .915 | .04 | | -.22; .29 | .768 | -.01 | | -.25 ;.24 | .965 | -.11 | -.38; .16 | .418 | .09 | -.17; .36 | .480 | .05 | -26 ;.35 | .768 |
| Observations | 65 |  |  | 65 |  |  | 65 | |  |  | 65 | |  |  | 65 |  |  | 65 | | | 65 | | |
| R^2^ / R^2^ Adjusted | .298/.263 |  |  | .438/.411 |  |  | .214/.176 | |  |  | .414/.386 | |  |  | .501/.476 |  |  | .257/.221 | | | .251/.214 | | |

**Note.** Models examined the effects of baseline and post-treatment **memory recognition bias** on residual symptoms at 2, 4, 8, 12, 16, 20, and 24 months, while controlling for concurrent residual symptoms. No significant recognition bias effects were observed.

**Table S12.** Mixed-effects model analysis of dysphoric self-referential activity in the frontal default mode network (DMN; superior frontal gyrus and paracingulate gyrus) in relapse and non-relapse patients with Major Depressive Disorder (MDD).

|  | Group effect | | | Group and Time effect | | | | Group x Time interaction | | | | Group effect at Time 1 | | | | Group effect at Time 2 | | | |
| --- | --- | --- | --- | --- | --- | --- | --- | --- | --- | --- | --- | --- | --- | --- | --- | --- | --- | --- | --- |
| *Predictors* | *Estimates* | *CI* | *p* | *Estimates* | *CI* | *p* | *Estimates* | | *CI* | *p* | *Estimates* | | *CI* | *p* | *Estimates* | | *CI* | *p* |  |
| (Intercept) | -.00 | -.18; .18 | .984 | .05 | -.14; .25 | .610 | .00 | | -.24; .25 | .970 | .00 | | -.25; .26 | .975 | -.01 | | -.25; .23 | .952 |  |
| Relapse (yes) | .17 | -.01; .34 | .064 | .16 | -.01; .34 | .070 | .32 | | .09; .55 | **.008** | .32 | | .08; .56 | **.010** | -.00 | | -.24; .23 | .984 |  |
| Time |  |  |  | -.10 | -.24; .04 | .152 | -.01 | | -.34; .31 | .941 |  | |  |  |  | |  |  |  |
| ADM (yes) | -.04 | -.20; .13 | .665 | -.04 | -.20; .13 | .666 | -.09 | | -.31; .13 | .404 | -.09 | | -.32; .13 | .415 | .03 | | -.19; .24 | .814 |  |
| Episodes | .01 | -.02; .04 | .690 | .01 | -.02; .04 | .695 | .01 | | -.03; .05 | .522 | .01 | | -.03; .06 | .529 | -.00 | | -.04; .04 | .944 |  |
| Symptoms | -.02 | -.10; .07 | .685 | -.01 | -.10; .08 | .799 | -.03 | | -.15; .09 | .666 | -.03 | | -.15; .10 | .651 | .01 | | -.11; .13 | .871 |  |
| Relapse x Time |  |  |  |  |  |  | -.32 | | -.64; -.01 | **.045** |  | |  |  |  | |  |  |  |
| ADM (yes) x Time |  |  |  |  |  |  | .12 | | -.17; .41 | .425 |  | |  |  |  | |  |  |  |
| Episodes x Time |  |  |  |  |  |  | -.01 | | -.07; .04 | .594 |  | |  |  |  | |  |  |  |
| Symptoms x Time |  |  |  |  |  |  | .04 | | -.13; .20 | .639 |  | |  |  |  | |  |  |  |
| **Random effects** |  |  |  |  |  |  |  | |  |  |  | |  |  |  | |  |  |  |
| σ^2^ | .21 |  |  | .20 |  |  | .20 | |  |  |  | |  |  |  | |  |  |  |
| τ₀₀ | .02_ID_ |  |  | . 02_ID_ |  |  | . 03_ID_ | |  |  |  | |  |  |  | |  |  |  |
| ICC | .10 |  |  | .11 |  |  | .12 | |  |  |  | |  |  |  | |  |  |  |
| N | 81_ID_ |  |  | 81_ID_ |  |  | 81_ID_ | |  |  |  | |  |  |  | |  |  |  |
| Observations | 161 |  |  | 161 |  |  | 161 | |  |  | 81 | |  |  | 80 | |  |  |  |
| Marginal R^2^ / Conditional R^2^ | .026/.124 |  |  | .037/141 |  |  | .062 / .171 | |  |  | .097/ .050 | |  |  | .001/-.052 | |  |  |  |

**Note.** A significant main effect of Group and a Group × Time interaction was observed. Post hoc models indicated that relapsers showed higher activation at baseline (Time 1) compared to non-relapsers, while no group difference was present at post-treatment (Time 2). All models controlled for antidepressant medication (ADM), number of past episodes, and residual symptoms. Dysphoric self-reference was indexed using the contrast Self_neg > Self_pos isolating neural responses to self-referential negative versus positive traits. Higher values indicate stronger negative self-referential bias.

**Table S13.** Mixed-effects model analysis of dysphoric self-referential activity in the frontal salience network (SN; anterior cingulate cortex) in relapse and non-relapse patients with Major Depressive Disorder (MDD).

|  | Group effect | | | Group and Time effect | | | | Group x Time interaction | | | | Group effect at Time 1 | | | | Group effect at Time 2 | | | |
| --- | --- | --- | --- | --- | --- | --- | --- | --- | --- | --- | --- | --- | --- | --- | --- | --- | --- | --- | --- |
| *Predictors* | *Estimates* | *CI* | *p* | *Estimates* | *CI* | *p* | *Estimates* | | *CI* | *p* | *Estimates* | | *CI* | *p* | *Estimates* | | *CI* | *p* |  |
| (Intercept) | -.17 | -.38; .05 | .134 | -.12 | -.36; .11 | .298 | -.22 | | -.51; .07 | .133 | -.22 | | -.50; .06 | .122 | -.12 | | -.41; .18 | .432 |  |
| Relapse (yes) | .21 | -.00; .42 | .051 | .21 | -.00; .42 | .055 | .36 | | .09; .63 | .**010** | .36 | | .09; .63 | **.009** | .05 | | -.24; .34 | .757 |  |
| Time |  |  |  | -.08 | -.24; .07 | .294 | .10 | | -.27; .47 | .582 |  | |  |  |  | |  |  |  |
| ADM (yes) | .03 | -.16; .23 | .728 | .03 | -.16; .23 | .728 | .09 | | -.16; .35 | .470 | .09 | | -.16; .35 | .461 | -.02 | | -.29; .25 | .881 |  |
| Episodes | .01 | -.03; .04 | .683 | .01 | -.03; .04 | .688 | .01 | | -.04; .06 | .716 | .01 | | -.04; .06 | .701 | .01 | | -.04; .06 | .799 |  |
| Symptoms | -.01 | -.11; .09 | .825 | -.01 | -.11; .10 | .918 | -.03 | | -.18; .11 | .632 | -.04 | | -.18; .10 | .568 | .02 | | -.12; .17 | .757 |  |
| Relapse x Time |  |  |  |  |  |  | -.32 | | -.67; .04 | .081 |  | |  |  |  | |  |  |  |
| ADM (yes) x Time |  |  |  |  |  |  | -.11 | | -.45; .22 | .495 |  | |  |  |  | |  |  |  |
| Episodes x Time |  |  |  |  |  |  | -.00 | | -.07; .06 | .937 |  | |  |  |  | |  |  |  |
| Symptoms x Time |  |  |  |  |  |  | .06 | | -.12; .25 | .504 |  | |  |  |  | |  |  |  |
| **Random effects** |  |  |  |  |  |  |  | |  |  |  | |  |  |  | |  |  |  |
| σ^2^ | .26 |  |  | .26 |  |  | .26 | |  |  |  | |  |  |  | |  |  |  |
| τ₀₀ | .05_ID_ |  |  | .05_ID_ |  |  | .05_ID_ | |  |  |  | |  |  |  | |  |  |  |
| ICC | .17 |  |  | .17 |  |  | .17 | |  |  |  | |  |  |  | |  |  |  |
| N | 81_ID_ |  |  | 81_ID_ |  |  | 81_ID_ | |  |  |  | |  |  |  | |  |  |  |
| Observations | 161 |  |  | 161 |  |  | 161 | |  |  | 81 | |  |  | 80 | |  |  |  |
| Marginal R^2^ / Conditional R^2^ | .033/.198 |  |  | .038/.205 |  |  | .057 / .221 | |  |  | .103/ .056 | |  |  | .005/-.048 | |  |  |  |

Note. A significant main effect of Group was observed. Post hoc models indicated that relapsers showed higher activation at baseline (Time 1) compared to non-relapsers, while no group difference was present at post-treatment (Time 2). All models controlled for antidepressant medication (ADM), number of past episodes, and residual symptoms. Dysphoric self-reference was indexed using the contrast Self_neg > Self_pos, isolating neural responses to self-referential negative versus positive traits. Higher values indicate stronger negative self-referential bias.

**Table SS14.** Cox **proportional hazards models of dysphoric** self-referential activity in the frontal default mode network (DMN; superior frontal gyrus and paracingulate gyrus) and the salience network (SN; anterior cingulate cortex)

|  | DMN | | | SN | | |
| --- | --- | --- | --- | --- | --- | --- |
| *Predictors* | *Estimates* | *CI* | *p* | *Estimates* | *CI* | *p* |
| ROI value (average) | 2.37 | .78; 7.15 | .127 | 2.51 | .95; 6.62 | .062 |
| Symptoms (Baseline) | 1.21 | .71; 2.06 | .473 | 1.22 | .72; 2.07 | .452 |
| Symptoms (Post-Intervention) | 1.57 | .99; 2.48 | .053 | 1.59 | 1.01; 2.50 | **.047** |
| ADM | 1.62 | .70; 3.79 | .263 | 1.42 | .60; 3.34 | .428 |
| Episodes | .97 | .83; 1.13 | .678 | .98 | .84; 1.14 | .787 |
| Observations | 80 |  |  | 80 |  |  |
| R^2^ Nagelkerke | .155 |  |  | .168 |  |  |

**Note.** Cox models assessed whether average dysphoric self-referential activity in the frontal DMN and SN predicted time-to-relapse while controlling for residual symptoms (baseline and post-treatment), ADM, and past episodes. Only post-treatment residual symptoms in the SN model were associated with greater relapse risk. Dysphoric self-reference was indexed using the contrast Self_neg > Self_pos , isolating neural responses to self-referential negative versus positive traits. Higher values indicate stronger negative self-referential bias.

**Table S15.** Mixed-effects model analysis of mean functional connectivity between frontal component of the default mode network (DMN; superior frontal gyrus and paracingulate gyrus) and the salience network (SN; anterior cingulate cortex) in relapse and non-relapse patients with Major Depression Disorder (MDD).

|  | Group effect | | | Group and Time effect | | | | Group x Time interaction | | | | Group effect at Time 1 | | | | Group effect at Time 2 | | | |
| --- | --- | --- | --- | --- | --- | --- | --- | --- | --- | --- | --- | --- | --- | --- | --- | --- | --- | --- | --- |
| *Predictors* | *Estimates* | *CI* | *p* | *Estimates* | *CI* | *p* | *Estimates* | | *CI* | *p* | *Estimates* | | *CI* | *p* | *Estimates* | | *CI* | *p* |  |
| (Intercept) | .17 | .15; .20 | **<.001** | .17 | .15; .20 | **<.001** | .17 | | .14; .20 | **<.001** | .17 | | .14; .21 | **<.001** | .17 | | .14; .20 | **<.001** |  |
| Relapse (yes) | -.01 | -.04; .01 | .383 | -.01 | -.04; .01 | .370 | -.02 | | -.05; .01 | .135 | -.02 | | -.05; .01 | .125 | -.00 | | -.03; .03 | .884 |  |
| Time |  |  |  | -.00 | -.02; .01 | .454 | -.00 | | -.03; .03 | .978 |  | |  |  |  | |  |  |  |
| ADM (yes) | .02 | -.01;.04 | .208 | .02 | -.01; .04 | .207 | .03 | | .00; .06 | **.046** | .03 | | -.00; .06 | .052 | .00 | | -.02; .03 | .832 |  |
| Episodes | .00 | .00; .01 | .393 | .00 | -.00; .01 | .396 | .00 | | -.00; .01 | .581 | .00 | | -.00; .01 | .625 | .00 | | -.00; .01 | .326 |  |
| Symptoms | -.00 | -.02; .01 | .411 | -.00 | -.01; .01 | .488 | -.00 | | -.02; .01 | .594 | -.00 | | -.02; .02 | .930 | -.00 | | -.02; .01 | .799 |  |
| Relapse x Time |  |  |  |  |  |  | .02 | | -.01; .05 | .125 |  | |  |  |  | |  |  |  |
| ADM (yes) x Time |  |  |  |  |  |  | -.03 | | -.05; .00 | .055 |  | |  |  |  | |  |  |  |
| Episodes x Time |  |  |  |  |  |  | .00 | | -.00; .01 | .653 |  | |  |  |  | |  |  |  |
| Symptoms x Time |  |  |  |  |  |  | -.00 | | -.02; .01 | .840 |  | |  |  |  | |  |  |  |
| **Random effects** |  |  |  |  |  |  |  | |  |  |  | |  |  |  | |  |  |  |
| σ^2^ | .00 |  |  | .00 |  |  | .00 | |  |  |  | |  |  |  | |  |  |  |
| τ₀₀ | .00_ID_ |  |  | .00_ID_ |  |  | .00_ID_ | |  |  |  | |  |  |  | |  |  |  |
| ICC | .56 |  |  | .55 |  |  | .57 | |  |  |  | |  |  |  | |  |  |  |
| N | 81_ID_ |  |  | 81_ID_ |  |  | 81_ID_ | |  |  |  | |  |  |  | |  |  |  |
| Observations | 161 |  |  | 161 |  |  | 161 | |  |  | 81 | |  |  | 80 | |  |  |  |
| Marginal R^2^ / Conditional R^2^ | .034/.574 |  |  | .035/.572 |  |  | .050 / .587 | |  |  | .075/.027 | |  |  | .014/-.038 | |  |  |  |

**Note.** No significant group effects or group x time interactions were observed across analysis. All models were controlled for antidepressant medication (ADM), number of past episodes, and Residual symptoms. Connectivity estimates were Fisher z-transformed

**Table S16.** Mixed-effects model analysis of mean functional connectivity within the frontal default mode network (DMN; superior frontal gyrus and paracingulate gyrus) in relapse and non-relapse patients with Major Depression Disorder (MDD).

|  | Group effect | | | Group and Time effect | | | | Group x Time interaction | | | | Group effect at Time 1 | | | | Group effect at Time 2 | | | |
| --- | --- | --- | --- | --- | --- | --- | --- | --- | --- | --- | --- | --- | --- | --- | --- | --- | --- | --- | --- |
| *Predictors* | *Estimates* | *CI* | *p* | *Estimates* | *CI* | *p* | *Estimates* | | *CI* | *p* | *Estimates* | | *CI* | *p* | *Estimates* | | *CI* | *p* |  |
| (Intercept) | .17 | .14; .19 | **<.001** | .17 | .14; .20 | **<.001** | .17 | | .14; .20 | **<.001** | .17 | | .14; .20 | **<.001** | .16 | | .13; .19 | **<.001** |  |
| Relapse (yes) | -.01 | -.03; .02 | .669 | -.01 | -.03; .02 | .644 | -.02 | | -.04; .01 | .280 | -.02 | | -.04; .01 | .287 | .00 | | -.03; .03 | .859 |  |
| Time |  |  |  | -.01 | -.02; .01 | .287 | -.01 | | -.04; .02 | .427 |  | |  |  |  | |  |  |  |
| ADM (yes) | .01 | -.01; .04 | .315 | .01 | -.01; .04 | .314 | .02 | | -.01; .05 | .152 | .02 | | -.01; .05 | .159 | .00 | | -.02; .03 | .733 |  |
| Episodes | .00 | -.00; .01 | .334 | .00 | -.00; .01 | .338 | .00 | | -.00; .01 | .619 | .00 | | -.00; .01 | .630 | .00 | | -.00; .01 | .227 |  |
| Symptoms | -.00 | -.01; .01 | .630 | -.00 | -.01; .01 | .758 | .00 | | -.01; .01 | .884 | .00 | | -.01; .02 | .864 | -.00 | | -.02; .01 | .872 |  |
| Relapse x Time |  |  |  |  |  |  | .02 | | -.01; .05 | .127 |  | |  |  |  | |  |  |  |
| ADM (yes) x Time |  |  |  |  |  |  | -.02 | | -.04; .01 | .214 |  | |  |  |  | |  |  |  |
| Episodes x Time |  |  |  |  |  |  | .00 | | -.00; .01 | .422 |  | |  |  |  | |  |  |  |
| Symptoms x Time |  |  |  |  |  |  | -.01 | | -.02; .01 | .453 |  | |  |  |  | |  |  |  |
| **Random effects** |  |  |  |  |  |  |  | |  |  |  | |  |  |  | |  |  |  |
| σ^2^ | .00 |  |  | . 00 |  |  | .00 | |  |  |  | |  |  |  | |  |  |  |
| τ₀₀ | .00_ID_ |  |  | .00_ID_ |  |  | .00_ID_ | |  |  |  | |  |  |  | |  |  |  |
| ICC | .60 |  |  | .60 |  |  | .60 | |  |  |  | |  |  |  | |  |  |  |
| N | 81_ID_ |  |  | 81_ID_ |  |  | 81_ID_ | |  |  |  | |  |  |  | |  |  |  |
| Observations | 161 |  |  | 161 |  |  | 161 | |  |  | 81 | |  |  | 80 | |  |  |  |
| Marginal R^2^ / Conditional R^2^ | .022/.610 |  |  | .024/.610 |  |  | .035 / .617 | |  |  | .039/-.011 | |  |  | .023/-.029 | |  |  |  |

**Note.** No significant main effects or interactions were observed across analysis. All models were controlled for antidepressant medication (ADM), number of past episodes, and Residual symptoms. Connectivity estimates were Fisher z-transformed

**Table S17.** Mixed-effects model analysis of mean functional connectivity within the frontal salience network (SN; anterior cingulate cortex) in relapse and non-relapse patients with Major Depression Disorder (MDD).

|  | Group effect | | | Group and Time effect | | | | Group x Time interaction | | | | Group effect at Time 1 | | | | Group effect at Time 2 | | | |
| --- | --- | --- | --- | --- | --- | --- | --- | --- | --- | --- | --- | --- | --- | --- | --- | --- | --- | --- | --- |
| *Predictors* | *Estimates* | *CI* | *p* | *Estimates* | *CI* | *p* | *Estimates* | | *CI* | *p* | *Estimates* | | *CI* | *p* | *Estimates* | | *CI* | *p* |  |
| (Intercept) | .28 | .25; .31 | **<.001** | .28 | .25; .31 | **<.001** | .27 | | .23; .30 | **<.001** | .27 | | .23; .31 | **<.001** | .29 | | .26; .32 | **<.001** |  |
| Relapse (yes) | -.01 | -.04; .02 | .455 | -.01 | -.04; .02 | .456 | -.02 | | -.05; .01 | .234 | -.02 | | -.06; .01 | .200 | -.00 | | -.03; .03 | .923 |  |
| Time |  |  |  | .00 | -.02; .02 | .924 | .02 | | -.02; .06 | .248 |  | |  |  |  | |  |  |  |
| ADM (yes) | .02 | -.01; .05 | .114 | .02 | -.01; .05 | .114 | .04 | | .01; .07 | **.017** | .04 | | .01; .07 | **.022** | .00 | | -.03; .03 | .771 |  |
| Episodes | .00 | -.00; .01 | .476 | .00 | -.00; .01 | .476 | .00 | | -.00; .01 | .397 | .00 | | -.00; .01 | .461 | .00 | | -.00; .01 | .704 |  |
| Symptoms | -.00 | -.02; .01 | .624 | -.00 | -.02; .01 | .625 | -.00 | | -.02; .01 | .655 | .00 | | -.02; .02 | .862 | -.00 | | -.02; .01 | .807 |  |
| Relapse x Time |  |  |  |  |  |  | .02 | | -.02; .06 | .274 |  | |  |  |  | |  |  |  |
| ADM (yes) x Time |  |  |  |  |  |  | -.03 | | -.07; -.00 | **.045** |  | |  |  |  | |  |  |  |
| Episodes x Time |  |  |  |  |  |  | -.00 | | -.01; .00 | .664 |  | |  |  |  | |  |  |  |
| Symptoms x Time |  |  |  |  |  |  | -.00 | | -.02; .02 | .944 |  | |  |  |  | |  |  |  |
| **Random effects** |  |  |  |  |  |  |  | |  |  |  | |  |  |  | |  |  |  |
| σ^2^ | .00 |  |  | .00 |  |  | .00 | |  |  |  | |  |  |  | |  |  |  |
| τ₀₀ | .00_ID_ |  |  | .00_ID_ |  |  | .00_ID_ | |  |  |  | |  |  |  | |  |  |  |
| ICC | .44 |  |  | .43 |  |  | .44 | |  |  |  | |  |  |  | |  |  |  |
| N | 81_ID_ |  |  | 81_ID_ |  |  | 81_ID_ | |  |  |  | |  |  |  | |  |  |  |
| Observations | 161 |  |  | 161 |  |  | 161 | |  |  | 81 | |  |  | 80 | |  |  |  |
| Marginal R^2^ / Conditional R^2^ | .033/.455 |  |  | .033/.451 |  |  | .050 / .469 | |  |  | .086/.038 | |  |  | .004/-.049 | |  |  |  |

**Note.** A significant main effect of antidepression medication (ADM) and an ADM × Time interaction was observed. Post hoc models indicated that patients who a history of ADM showed higher activation at baseline (Time 1) compared to those without a history of ADM, while no difference were observed at post-treatment (Time 2). Connectivity estimates were Fisher z-transformed

**Table S18. Static neural markers of relapse vulnerability.**

|  |  |  |  | Cluster size |  | MNI coordinates (mm) | | |
| --- | --- | --- | --- | --- | --- | --- | --- | --- |
| *Description* | *Region* | *Side* | *p (FDR)* | *k* | *z-score* | *x* | *y* | *z* |
| Relapse > Non-Relapse | Subgenual cingulate | L | .03 | 550 | 4.80 | -2 | 48 | -12 |
|  | Lateral occipital cortex | R | .03 | 546 | 4.79 | 50 | -74 | 44 |
| Non-Relapse > Relapse | Postcentral gyrus/ Primary somatosensory | R | .07 | 508 | 3.80 | 50 | -26 | 42 |

**Note. Results from** a mixed 2 (time: wave 1, wave 2) × 2 (relapse status: relapse, non-relapse) × 2 ( treatment group: MBCT, WB-CT) factorial model examining self-referential processing (Self vs Case). Whole-brain effects were thresholded at p < .005, cluster extent k = 400, and FDR corrected for multiple comparisons. Relapsers showed greater activation in the left subgenual cingulate and right lateral occipital cortex, whereas non-relapsers showed greater activation in the right postcentral gyrus/primary somatosensory cortex.

**Table S19.** Mixed-effects model analysis of mean activation differences in the left subgenual cingulate between relapse and non-relapse patients with Major Depression Disorder (MDD).

|  | Group effect | | | Group and Time effect | | | | Group x Time interaction | | | | Group effect at Time 1 | | | | Group effect at Time 2 | | | |
| --- | --- | --- | --- | --- | --- | --- | --- | --- | --- | --- | --- | --- | --- | --- | --- | --- | --- | --- | --- |
| *Predictors* | *Estimates* | *CI* | *p* | *Estimates* | *CI* | *p* | *Estimates* | | *CI* | *p* | *Estimates* | | *CI* | *p* | *Estimates* | | *CI* | *p* |  |
| (Intercept) | 1.32 | .89; 1.75 | **<.001** | 1.34 | .90; 1.78 | **<.001** | 1.37 | | .88; 1.86 | **<.001** | 1.41 | | .90; 1.92 | **<.001** | 1.27 | | .80; 1.75 | **<.001** |  |
| Relapse (yes) | .74 | .33; 1.15 | **<.001** | .74 | .33; 1.14 | **<.001** | 0.74 | | .28; 1.20 | **.002** | .69 | | .21; 1.17 | **.005** | .69 | | .22; 1.16 | **.005** |  |
| Time |  |  |  | -.03 | -.23; .17 | .775 | -.11 | | -.57; .36 | .656 |  | |  |  |  | |  |  |  |
| ADM (yes) | -.35 | -.74; .03 | .073 | -.35 | -.74; .03 | .073 | -.27 | | -.71; .17 | .226 | -.27 | | -.72; .19 | .248 | -.43 | | -.87; .00 | .052 |  |
| Episodes | .00 | -.07 ; .08 | .914 | .00 | -.07 ; .08 | .917 | -.02 | | -.10; .06 | .647 | -.02 | | -.11; .06 | .583 | .03 | | -.06; .11 | .539 |  |
| Symptoms | .08 | -.09 ; .25 | .347 | .08 | -.08 ; .25 | .327 | .09 | | -.13; .30 | .423 | .18 | | -.07; .44 | .148 | .15 | | -.09; .38 | .221 |  |
| Relapse x Time |  |  |  |  |  |  | -.02 | | -.47; .44 | .944 |  | |  |  |  | |  |  |  |
| ADM (yes) x Time |  |  |  |  |  |  | -.17 | | -.59 ;.26 | .440 |  | |  |  |  | |  |  |  |
| Episodes x Time |  |  |  |  |  |  | .05 | | -.03; .13 | .256 |  | |  |  |  | |  |  |  |
| Symptoms x Time |  |  |  |  |  |  | .00 | | -.25 ; .26 | .979 |  | |  |  |  | |  |  |  |
| **Random effects** |  |  |  |  |  |  |  | |  |  |  | |  |  |  | |  |  |  |
| σ^2^ | .40 |  |  | . 41 |  |  | . 42 | |  |  |  | |  |  |  | |  |  |  |
| τ₀₀ | .51_ID_ |  |  | . 50_ID_ |  |  | . 50_ID_ | |  |  |  | |  |  |  | |  |  |  |
| ICC | .56 |  |  | .55 |  |  | .54 | |  |  |  | |  |  |  | |  |  |  |
| N | 81_ID_ |  |  | 81_ID_ |  |  | 81_ID_ | |  |  |  | |  |  |  | |  |  |  |
| Observations | 161 |  |  | 161 |  |  | 161 | |  |  | 81 | |  |  | 80 | |  |  |  |
| Marginal R^2^ / Conditional R^2^ | .151/.624 |  |  | .152/.620 |  |  | .155 / .614 | |  |  | .158/.114 | |  |  | .194/.151 | |  |  |  |

**Note.** A main effect of Group was significant across analysis. No significant Time effect or Group × Time interaction was found. All models were controlled for antidepressant medication (ADM), number of past episodes, and Residual symptoms.

**Table S20.** Mixed-effects model analysis of mean activation differences in the right lateral occipital cortex between relapse and non-relapse patients with Major Depression Disorder (MDD).

|  | Group effect | | | Group and Time effect | | | | Group x Time interaction | | | | Group effect at Time 1 | | | | Group effect at Time 2 | | | |
| --- | --- | --- | --- | --- | --- | --- | --- | --- | --- | --- | --- | --- | --- | --- | --- | --- | --- | --- | --- |
| *Predictors* | *Estimates* | *CI* | *p* | *Estimates* | *CI* | *p* | *Estimates* | | *CI* | *p* | *Estimates* | | *CI* | *p* | *Estimates* | | *CI* | *p* |  |
| (Intercept) | -.20 | -.52; .12 | .214 | -.17 | -.50 ;.15 | .296 | -.13 | | -.48; .22 | .472 | -.10 | | -.45; .26 | .586 | -.27 | | -.62; .08 | .133 |  |
| Relapse (yes) | .55 | .25; .85 | **<.001** | .55 | .24; .85 | **<.001** | .61 | | .28; .95 | **<.001** | .57 | | .23; .91 | **.001** | .40 | | .05; .75 | **.024** |  |
| Time |  |  |  | -.05 | -.19; .09 | .462 | -.15 | | -.46; .16 | .341 |  | |  |  |  | |  |  |  |
| ADM (yes) | .01 | -.28; .30 | .953 | .01 | -.28; .30 | .952 | -.11 | | -.21; .43 | .503 | .11 | | -.21; .43 | .483 | -.08 | | -.40; .24 | .632 |  |
| Episodes | -.00 | -.06 ; .05 | .884 | -.00 | -.06 ; .05 | .875 | -.04 | | -.10; .02 | .230 | -.04 | | -.10; .02 | .186 | .03 | | -.03; .09 | .384 |  |
| Symptoms | .02 | -.10 ; .14 | .706 | .03 | -.09 ; .15 | .617 | -.01 | | -.16; .14 | .899 | .08 | | -.10; .25 | .387 | .18 | | .01; .36 | **.037** |  |
| Relapse x Time |  |  |  |  |  |  | -.14 | | -.44; .16 | .348 |  | |  |  |  | |  |  |  |
| ADM (yes) x Time |  |  |  |  |  |  | -.20 | | -.47 ;.08 | .168 |  | |  |  |  | |  |  |  |
| Episodes x Time |  |  |  |  |  |  | .07 | | .01; .12 | **.015** |  | |  |  |  | |  |  |  |
| Symptoms x Time |  |  |  |  |  |  | .08 | | -.08 ;.25 | .325 |  | |  |  |  | |  |  |  |
| **Random effects** |  |  |  |  |  |  |  | |  |  |  | |  |  |  | |  |  |  |
| σ^2^ | .19 |  |  | . 19 |  |  | . 18 | |  |  |  | |  |  |  | |  |  |  |
| τ₀₀ | .29_ID_ |  |  | . 29_ID_ |  |  | . 30_ID_ | |  |  |  | |  |  |  | |  |  |  |
| ICC | .61 |  |  | .60 |  |  | .62 | |  |  |  | |  |  |  | |  |  |  |
| N | 81_ID_ |  |  | 81_ID_ |  |  | 81_ID_ | |  |  |  | |  |  |  | |  |  |  |
| Observations | 161 |  |  | 161 |  |  | 161 | |  |  | 81 | |  |  | 80 | |  |  |  |
| Marginal R^2^ / Conditional R^2^ | .128/.656 |  |  | .129/.653 |  |  | .149 / .677 | |  |  | .174/.131 | |  |  | .182/.138 | |  |  |  |

**Note.** A main effect of Group was significant across analysis. Significant Episodes × Time interaction indicated increased activation among patients with higher number of past episodes at follow-up. However, no group × time interaction was found. All models were controlled for antidepressant medication (ADM), number of past episodes, and Residual symptoms.

**Table S21.** Mixed-effects model analysis of mean activation differences in the right postcentral gyrus/ primary somatosensory between relapse and non-relapse patients with Major Depression Disorder (MDD).

|  | Group effect | | | Group and Time effect | | | | Group x Time interaction | | | | Group effect at Time 1 | | | | Group effect at Time 2 | | | |
| --- | --- | --- | --- | --- | --- | --- | --- | --- | --- | --- | --- | --- | --- | --- | --- | --- | --- | --- | --- |
| *Predictors* | *Estimates* | *CI* | *p* | *Estimates* | *CI* | *p* | *Estimates* | | *CI* | *p* | *Estimates* | | *CI* | *p* | *Estimates* | | *CI* | *p* |  |
| (Intercept) | -.08 | -.27; .10 | .374 | -.13 | -.33; .07 | .190 | -.24 | | -.48; .00 | .050 | -.24 | | -.46; -.02 | **.037** | .08 | | -.18; .34 | .541 |  |
| Relapse (yes) | -.33 | -.51; -.15 | **<.001** | -.33 | -.51; -.15 | **<.001** | -.34 | | -.57; -.11 | **.004** | -.34 | | -.56; -.13 | **.002** | -.30 | | -.56; -.04 | **.022** |  |
| Time |  |  |  | .09 | -.04; .23 | .165 | .32 | | .02; .62 | **.037** |  | |  |  |  | |  |  |  |
| ADM (yes) | .00 | -.16; .17 | .956 | .00 | -.16; .17 | .956 | .18 | | -.04; .40 | .103 | .18 | | -.02; .38 | .078 | -.18 | | -.41; .06 | .143 |  |
| Episodes | -.00 | -.03 ;.03 | .936 | -.00 | -.03 ;.03 | .951 | .00 | | -.04; .04 | .990 | -.00 | | -.04; .04 | .979 | -.00 | | -.05; .04 | .893 |  |
| Symptoms | -.04 | -.12 ;.05 | .400 | -.04 | -.13 ;.04 | .318 | .00 | | -.12; .11 | .937 | .01 | | -.11 ;.11 | .990 | -.08 | | -.21; .04 | .193 |  |
| Relapse x Time |  |  |  |  |  |  | .04 | | -.25; .33 | .773 |  | |  |  |  | |  |  |  |
| ADM (yes) x Time |  |  |  |  |  |  | -.35 | | -.62; -.08 | **.011** |  | |  |  |  | |  |  |  |
| Episodes x Time |  |  |  |  |  |  | -.00 | | -.05; .05 | .922 |  | |  |  |  | |  |  |  |
| Symptoms x Time |  |  |  |  |  |  | -.08 | | -.24; .07 | .295 |  | |  |  |  | |  |  |  |
| **Random effects** |  |  |  |  |  |  |  | |  |  |  | |  |  |  | |  |  |  |
| σ^2^ | .18 |  |  | . 18 |  |  | . 17 | |  |  |  | |  |  |  | |  |  |  |
| τ₀₀ | .04_ID_ |  |  | . 04_ID_ |  |  | . 05_ID_ | |  |  |  | |  |  |  | |  |  |  |
| ICC | .19 |  |  | .20 |  |  | .22 | |  |  |  | |  |  |  | |  |  |  |
| N | 81_ID_ |  |  | 81_ID_ |  |  | 81_ID_ | |  |  |  | |  |  |  | |  |  |  |
| Observations | 161 |  |  | 161 |  |  | 161 | |  |  | 81 | |  |  | 80 | |  |  |  |
| Marginal R^2^ / Conditional R^2^ | .113/.285 |  |  | .122/.298 |  |  | .152 / .339 | |  |  | .152/.107 | |  |  | .154/.108 | |  |  |  |

**Note.** A significant main effect of group was observed, with relapsers showing lower activation than non-relapsers across analysis. A main effect of time was also detected, and an ADM × time interaction indicated reduced activation among ADM users at follow-up. However, no group × time interaction was found. In follow-up models stratified by time, group differences remained significant at both baseline (Time 1) and post-intervention (Time 2). All models were controlled for antidepressant medication (ADM), number of past episodes, and Residual symptoms.

**Table S22.** Cox **proportional hazards models of static neural markers predicting relapse.**

|  | L. Subgenual Cingulate | | | R. Lateral occipital Cortex | | | R. Postcentral Gyrus | | |
| --- | --- | --- | --- | --- | --- | --- | --- | --- | --- |
| *Predictors* | *Estimates* | *CI* | *p* | *Estimates* | *CI* | *p* | *Estimates* | *CI* | *p* |
| ROI value (average) | 1.81 | 1.20 ; 2.72 | **.004** | 2.07 | 1.12; 3.84 | **.021** | .26 | .12 ;.58 | **<.001** |
| Symptoms (Baseline) | 1.00 | .57 ; 1.74 | .989 | 1.05 | .62; 1.75 | .866 | 1.04 | .60; 1.80 | .887 |
| Symptoms (Post-Intervention) | 1.46 | .90 ; 2.37 | .124 | 1.39 | .83; 2.34 | .208 | 1.59 | .97; 2.58 | .064 |
| ADM | 1.51 | .65; 3.51 | .334 | 1.51 | .63; 3.62 | .351 | 1.65 | .70; 3.90 | .254 |
| Episodes | 1.04 | .89; 1.21 | .634 | .99 | .84; 1.15 | .862 | 1.03 | .88; 1.19 | .735 |
| Observations | 81 |  |  | 81 |  |  | 81 |  |  |
| R^2^ Nagelkerke | .208 |  |  | .180 |  |  | .228 |  |  |

**Note.** Cox regression models examined whether average activation in the left subgenual cingulate, right lateral occipital cortex, and right postcentral gyrus predicted time-to-relapse, controlling for concurrent residual symptoms at baseline and post-treatment, antidepressant medication (ADM), and past episodes. Higher activation in the subgenual cingulate and lateral occipital cortex predicted increased relapse risk, whereas higher postcentral activation was associated with reduced relapse risk

**Table S23.** Dynamic neural markers of treatment-related non-relapse growth (conjunction analysis).

|  |  |  |  | Cluster size |  | MNI coordinates (mm) | | |
| --- | --- | --- | --- | --- | --- | --- | --- | --- |
| *Description* | *Region* | *Side* | *p (FDR)* | *k* | *z-score* | *x* | *y* | *z* |
| Conjunction (Dynamic Treatment response) | Cerebellum | L | .976 | 496 | 2.96 | -22 | -56 | -60 |
|  | Supramarginal gyrus | L | .976 | 456 | 2.92 | -58 | -48 | 18 |
|  | Precentral gyrus | R | .976 | 628 | 2.70 | 34 | -14 | 62 |
|  | Lateral occipital cortex | R | .976 | 588 | 2.63 | 20 | -66 | 42 |
|  | Superior temporal gyrus | R | .976 | 567 | 2.63 | 44 | -14 | -6 |
|  | Inferior frontal gyrus | R | .976 | 533 | 2.71 | 40 | 10 | 22 |

**Note.** An exploratory conjunction analysis identified regions showing consistent increases among non-relapsers across both time points (non-relapse growth), as well as regions where growth was specific to the post-intervention session. Conjunction results were thresholded at p < √0.005 (equivalent to conjoint probability control across contrasts), with a cluster extent of k = 400 voxels. Significant clusters were observed predominantly within **somatosensory and associative cortices**, including the bilateral occipital cortex, left supramarginal, and right precentral, superior temporal, and inferior frontal gyrus.

**Table S24.** Mixed-effects model analysis of mean activation differences in the L. cerebellum between relapse and non-relapse patients with Major Depression Disorder (MDD).

|  | Group effect | | | Group and Time effect | | | | Group x Time interaction | | | | Group effect at Time 1 | | | | Group effect at Time 2 | | | |
| --- | --- | --- | --- | --- | --- | --- | --- | --- | --- | --- | --- | --- | --- | --- | --- | --- | --- | --- | --- |
| *Predictors* | *Estimates* | *CI* | *p* | *Estimates* | *CI* | *p* | *Estimates* | | *CI* | *p* | *Estimates* | | *CI* | *p* | *Estimates* | | *CI* | *p* |  |
| (Intercept) | .00 | -.18; .17 | .980 | -.09 | -.27; .10 | .357 | -.17 | | -.40; .06 | .140 | -.17 | | -.37; .04 | .104 | -.16 | | -.09; .42 | .205 |  |
| Relapse (yes) | -.14 | -.31; .03 | .102 | -.13 | -.30; .03 | .118 | .06 | | -.16; .28 | .592 | .05 | | -.14; .24 | .590 | -.33 | | -.58; -.08 | **.011** |  |
| Time |  |  |  | .16 | .03; .30 | **.017** | .34 | | .04; .64 | **.027** |  | |  |  |  | |  |  |  |
| ADM (yes) | .02 | -.14; .18 | .784 | .02 | -.14; .18 | .786 | .10 | | -.11; .30 | .358 | .10 | | -.08; .28 | .290 | -.05 | | -.28; .18 | .661 |  |
| Episodes | -.01 | -.04 ;.02 | .520 | -.01 | -.04; .02 | .542 | -.02 | | -.05; .02 | .432 | -.02 | | -.05; .02 | .349 | -.00 | | -.05; .04 | .903 |  |
| Symptoms | .03 | -.06; .11 | .523 | .01 | -.07; .10 | .722 | .02 | | -.05; .02 | .432 | .03 | | -.07; .13 | .538 | .02 | | -.11; .14 | .772 |  |
| Relapse x Time |  |  |  |  |  |  | -.39 | | -.68; -.10 | **.008** |  | |  |  |  | |  |  |  |
| ADM (yes) x Time |  |  |  |  |  |  | -.15 | | -.42; .12 | .281 |  | |  |  |  | |  |  |  |
| Episodes x Time |  |  |  |  |  |  | .01 | | -.04; .06 | .618 |  | |  |  |  | |  |  |  |
| Symptoms x Time |  |  |  |  |  |  | .00 | | -.15 ;.16 | .950 |  | |  |  |  | |  |  |  |
| **Random effects** |  |  |  |  |  |  |  | |  |  |  | |  |  |  | |  |  |  |
| σ^2^ | .19 |  |  | .18 |  |  | .17 | |  |  |  | |  |  |  | |  |  |  |
| τ₀₀ | .02_ID_ |  |  | . 02_ID_ |  |  | . 03_ID_ | |  |  |  | |  |  |  | |  |  |  |
| ICC | .09 |  |  | .12 |  |  | .15 | |  |  |  | |  |  |  | |  |  |  |
| N | 81_ID_ |  |  | 81_ID_ |  |  | 81_ID_ | |  |  |  | |  |  |  | |  |  |  |
| Observations | 161 |  |  | 161 |  |  | 161 | |  |  | 81 | |  |  | 80 | |  |  |  |
| Marginal R^2^ / Conditional R^2^ | .022/.107 |  |  | .052/164 |  |  | .097 / .235 | |  |  | .034/ -.017 | |  |  | .095/.047 | |  |  |  |

**Note.** A significant main effect of Time and a Group × Time interaction was observed. Post hoc models indicated that non-relapsers showed higher activation at post-treatment (Time 2) compared to relapsers, while no group difference was present at baseline (Time 1). All models controlled for antidepressant medication (ADM), number of past episodes, and residual symptoms.

**Table SS25.** Mixed-effects model analysis of mean activation differences in the L. supramarginal gyrus between relapse and non-relapse patients with Major Depression Disorder (MDD).

|  | Group effect | | | Group and Time effect | | | | Group x Time interaction | | | | Group effect at Time 1 | | | | Group effect at Time 2 | | | |
| --- | --- | --- | --- | --- | --- | --- | --- | --- | --- | --- | --- | --- | --- | --- | --- | --- | --- | --- | --- |
| *Predictors* | *Estimates* | *CI* | *p* | *Estimates* | *CI* | *p* | *Estimates* | | *CI* | *p* | *Estimates* | | *CI* | *p* | *Estimates* | | *CI* | *p* |  |
| (Intercept) | .10 | -.05; .26 | .182 | .06 | -.11; .22 | .493 | .02 | | -.16; .21 | .804 | .04 | | -.14; .22 | .702 | .18 | | -.01; .37 | .067 |  |
| Relapse (yes) | -.16 | -.31; -.01 | **.036** | -.15 | -.30; -.01 | **.402** | -.03 | | -.20; .15 | .777 | -.04 | | -.22; .14 | .636 | -.29 | | -.48; -.11 | **.003** |  |
| Time |  |  |  | .09 | .00; .19 | .052 | .15 | | -.05; .36 | .148 |  | |  |  |  | |  |  |  |
| ADM (yes) | -.10 | -.24; .04 | .172 | -.10 | -.24; .04 | .172 | -.06 | | -.23; .11 | .462 | -.06 | | -.23; .11 | .472 | -.13 | | -.30; .04 | .140 |  |
| Episodes | .00 | -.02; .03 | .723 | .01 | -.02; .03 | .702 | -.00 | | -.04; .03 | .850 | -.00 | | -.04 ; .03 | .771 | .01 | | -.02; .05 | .405 |  |
| Symptoms | .03 | -.04; .10 | .368 | .02 | -.05; .09 | .536 | -.00 | | -.09; .09 | .993 | .03 | | -.06; .13 | .460 | .05 | | -.04; .15 | .269 |  |
| Relapse x Time |  |  |  |  |  |  | -.27 | | -.47; -.07 | **.010** |  | |  |  |  | |  |  |  |
| ADM (yes) x Time |  |  |  |  |  |  | -.07 | | -.25 ; .12 | .490 |  | |  |  |  | |  |  |  |
| Episodes x Time |  |  |  |  |  |  | .02 | | -.02; .05 | .345 |  | |  |  |  | |  |  |  |
| Symptoms x Time |  |  |  |  |  |  | .05 | | -.06; .16 | .371 |  | |  |  |  | |  |  |  |
| **Random effects** |  |  |  |  |  |  |  | |  |  |  | |  |  |  | |  |  |  |
| σ^2^ | .09 |  |  | .09 |  |  | .08 | |  |  |  | |  |  |  | |  |  |  |
| τ₀₀ | .05_ID_ |  |  | . 05_ID_ |  |  | . 05_ID_ | |  |  |  | |  |  |  | |  |  |  |
| ICC | .34 |  |  | .36 |  |  | .39 | |  |  |  | |  |  |  | |  |  |  |
| N | 81_ID_ |  |  | 81_ID_ |  |  | 81_ID_ | |  |  |  | |  |  |  | |  |  |  |
| Observations | 161 |  |  | 161 |  |  | 161 | |  |  | 81 | |  |  | 80 | |  |  |  |
| Marginal R^2^ / Conditional R^2^ | .056/.376 |  |  | .068/.404 |  |  | .096 / .450 | |  |  | .017/ -.035 | |  |  | .151/.106 | |  |  |  |

Note. A significant Group × Time interaction was observed. Post hoc models indicated that non-relapsers showed higher activation at post-treatment (Time 2) compared to relapsers, while no group difference was present at baseline (Time 1). All models controlled for antidepressant medication (ADM), number of past episodes, and residual symptoms.

**Table S26.** Mixed-effects model analysis of mean activation differences in the R. precentral gyrus between relapse and non-relapse patients with Major Depression Disorder (MDD).

|  | Group effect | | | Group and Time effect | | | | Group x Time interaction | | | | Group effect at Time 1 | | | | Group effect at Time 2 | | | |
| --- | --- | --- | --- | --- | --- | --- | --- | --- | --- | --- | --- | --- | --- | --- | --- | --- | --- | --- | --- |
| *Predictors* | *Estimates* | *CI* | *p* | *Estimates* | *CI* | *p* | *Estimates* | | *CI* | *p* | *Estimates* | | *CI* | *p* | *Estimates* | | *CI* | *p* |  |
| (Intercept) | -.04 | -.19; .12 | .635 | -.09 | -.25; .08 | .295 | -.23 | | -.41; -.04 | **.021** | -.22 | | -.39; -.05 | **.011** | .15 | | -.06; .36 | .163 |  |
| Relapse (yes) | -.11 | -.26; .04 | .151 | -.10 | -.25; .04 | .168 | .04 | | -.14; .22 | .667 | .04 | | -.12; .20 | .656 | -.26 | | -.46; -.05 | **.015** |  |
| Time |  |  |  | .10 | -.01; .20 | .068 | .37 | | .15; .59 | **.001** |  | |  |  |  | |  |  |  |
| ADM (yes) | -.10 | -.24; .04 | .166 | -.10 | -.24; .04 | .167 | .05 | | -.12; .22 | .596 | .05 | | -.11; .20 | .548 | -.24 | | -.43; -.05 | **.013** |  |
| Episodes | .01 | -.02; .03 | .695 | .01 | -.02; .03 | .677 | .01 | | -.03; .04 | .755 | .00 | | -.02; .03 | .741 | .01 | | -.03; .04 | .756 |  |
| Symptoms | -.01 | -.08; .06 | .818 | -.02 | -.09; .05 | .642 | -.02 | | -.12; .07 | .594 | -.02 | | -.10; .07 | .668 | .01 | | -.09; .11 | .875 |  |
| Relapse x Time |  |  |  |  |  |  | -.29 | | -.50; -.08 | **.008** |  | |  |  |  | |  |  |  |
| ADM (yes) x Time |  |  |  |  |  |  | -.29 | | -.48; -.09 | **.005** |  | |  |  |  | |  |  |  |
| Episodes x Time |  |  |  |  |  |  | .00 | | -.04; .04 | .951 |  | |  |  |  | |  |  |  |
| Symptoms x Time |  |  |  |  |  |  | .02 | | -.10; .13 | .785 |  | |  |  |  | |  |  |  |
| **Random effects** |  |  |  |  |  |  |  | |  |  |  | |  |  |  | |  |  |  |
| σ^2^ | .11 |  |  | . 11 |  |  | . 09 | |  |  |  | |  |  |  | |  |  |  |
| τ₀₀ | .04_ID_ |  |  | . 04_ID_ |  |  | . 05_ID_ | |  |  |  | |  |  |  | |  |  |  |
| ICC | .24 |  |  | .26 |  |  | .33 | |  |  |  | |  |  |  | |  |  |  |
| N | 81_ID_ |  |  | 81_ID_ |  |  | 81_ID_ | |  |  |  | |  |  |  | |  |  |  |
| Observations | 161 |  |  | 161 |  |  | 161 | |  |  | 81 | |  |  | 80 | |  |  |  |
| Marginal R^2^ / Conditional R^2^ | .036/.270 |  |  | .051/.300 |  |  | .116 / .412 | |  |  | .011/ -.041 | |  |  | .162/.118 | |  |  |  |

**Note.** A significant main effect of Time, Group × Time, and ADM x Time interactions were observed. Post hoc models indicated that non-relapsers showed higher activation at post-treatment (Time 2) compared to relapsers, while no group difference was present at baseline (Time 1). All models controlled for antidepressant medication (ADM), number of past episodes, and residual symptoms.

**Table S27.** Mixed-effects model analysis of mean activation differences in the R. superior temporal gyrus between relapse and non-relapse patients with Major Depression Disorder (MDD).

|  | Group effect | | | Group and Time effect | | | | Group x Time interaction | | | | Group effect at Time 1 | | | | Group effect at Time 2 | | | |
| --- | --- | --- | --- | --- | --- | --- | --- | --- | --- | --- | --- | --- | --- | --- | --- | --- | --- | --- | --- |
| *Predictors* | *Estimates* | *CI* | *p* | *Estimates* | *CI* | *p* | *Estimates* | | *CI* | *p* | *Estimates* | | *CI* | *p* | *Estimates* | | *CI* | *p* |  |
| (Intercept) | -.17 | -.33; -.01 | **.040** | -.26 | -.43; -.08 | **.004** | -.33 | | -.54; -.12 | **.002** | -.33 | | -.53; -.12 | **.002** | -.03 | | -.24 ;.19 | .790 |  |
| Relapse (yes) | -.15 | -.30; .01 | .064 | -.14 | -.30; .01 | .073 | -.01 | | -.20; .19 | .960 | -.01 | | -.20; .19 | .948 | -.29 | | -.50; -.07 | **.009** |  |
| Time |  |  |  | .17 | .05; .28 | **.005** | .30 | | .04; .56 | **.025** |  | |  |  |  | |  |  |  |
| ADM (yes) | -.08 | -.22; .07 | .293 | -.08 | -.22; .07 | .292 | -.05 | | -.24; .14 | .598 | -.05 | | -.23; .13 | .588 | -.10 | | -.30; .09 | .302 |  |
| Episodes | .01 | -.02; .04 | .521 | .01 | -.02; .04 | .499 | .01 | | -.02; .05 | .536 | .01 | | -.02; .05 | .527 | .01 | | -.03 ; .05 | .627 |  |
| Symptoms | -.02 | -.10; .05 | .572 | -.03 | -.11 ; .04 | .388 | -.08 | | -.18; .02 | .117 | -.08 | | -.18; .02 | .125 | .01 | | -.09; .12 | .833 |  |
| Relapse x Time |  |  |  |  |  |  | -.29 | | -.54; -.03 | **.027** |  | |  |  |  | |  |  |  |
| ADM (yes) x Time |  |  |  |  |  |  | -.05 | | -.29; .19 | .669 |  | |  |  |  | |  |  |  |
| Episodes x Time |  |  |  |  |  |  | -.00 | | -.05 ;.04 | .918 |  | |  |  |  | |  |  |  |
| Symptoms x Time |  |  |  |  |  |  | .10 | | -.03; .24 | .138 |  | |  |  |  | |  |  |  |
| **Random effects** |  |  |  |  |  |  |  | |  |  |  | |  |  |  | |  |  |  |
| σ^2^ | .15 |  |  | . 14 |  |  | . 13 | |  |  |  | |  |  |  | |  |  |  |
| τ₀₀ | .03_ID_ |  |  | . 03_ID_ |  |  | . 04_ID_ | |  |  |  | |  |  |  | |  |  |  |
| ICC | .15 |  |  | .19 |  |  | .22 | |  |  |  | |  |  |  | |  |  |  |
| N | 81_ID_ |  |  | 81_ID_ |  |  | 81_ID_ | |  |  |  | |  |  |  | |  |  |  |
| Observations | 161 |  |  | 161 |  |  | 161 | |  |  | 81 | |  |  | 80 | |  |  |  |
| Marginal R^2^ / Conditional R^2^ | .044/.190 |  |  | .082/.258 |  |  | .108/ .300 | |  |  | .039/-.012 | |  |  | .111/.064 | |  |  |  |

**Note.** A significant main effect of Time and a Group × Time interactions were observed. Post hoc models indicated that non-relapsers showed higher activation at post-treatment (Time 2) compared to relapsers, while no group difference was present at baseline (Time 1). All models controlled for antidepressant medication (ADM), number of past episodes, and residual symptoms.

**Table S28.** Mixed-effects model analysis of mean activation differences in the R. inferior frontal gyrus between relapse and non-relapse patients with Major Depression Disorder (MDD).

|  | Group effect | | | Group and Time effect | | | | Group x Time interaction | | | | Group effect at Time 1 | | | | Group effect at Time 2 | | | |
| --- | --- | --- | --- | --- | --- | --- | --- | --- | --- | --- | --- | --- | --- | --- | --- | --- | --- | --- | --- |
| *Predictors* | *Estimates* | *CI* | *p* | *Estimates* | *CI* | *p* | *Estimates* | | *CI* | *p* | *Estimates* | | *CI* | *p* | *Estimates* | | *CI* | *p* |  |
| (Intercept) | -.03 | -.18; .12 | .682 | -.08 | -.23; .07 | .307 | -.17 | | -.35; .01 | .057 | -.16 | | -.33; .01 | .066 | .10 | | -.08; .28 | .258 |  |
| Relapse (yes) | -.14 | -.28; .00 | **.049** | -.13 | -.28; .01 | .060 | -.01 | | -.18; .15 | .892 | -.02 | | -.19; .14 | .783 | -.28 | | -.45; -.10 | **.002** |  |
| Time |  |  |  | .09 | .01 ; .18 | **.032** | .27 | | .08; .46 | **.006** |  | |  |  |  | |  |  |  |
| ADM (yes) | .02 | -.11; .15 | .749 | .02 | -.11; .15 | .753 | .08 | | -.08; .24 | .307 | .08 | | -.07; .24 | .289 | -.04 | | -.20; .13 | .647 |  |
| Episodes | -.00 | -.03; .02 | .749 | .00 | -.03 ; .02 | .777 | -.00 | | -.03; .03 | .936 | -.00 | | -.03 ; .03 | .878 | -.01 | | -.04; .02 | .680 |  |
| Symptoms | .00 | -.06; .07 | .943 | -.01 | -.07; .06 | .790 | -.03 | | -.11 ;.05 | .465 | -.01 | | -.09; .08 | .866 | .04 | | -.05 ;.13 | .340 |  |
| Relapse x Time |  |  |  |  |  |  | -.25 | | -.44; -.07 | **.007** |  | |  |  |  | |  |  |  |
| ADM (yes) x Time |  |  |  |  |  |  | -.12 | | -.29; .05 | .171 |  | |  |  |  | |  |  |  |
| Episodes x Time |  |  |  |  |  |  | -.00 | | -.04 ;.03 | .798 |  | |  |  |  | |  |  |  |
| Symptoms x Time |  |  |  |  |  |  | .05 | | -.05; .14 | .374 |  | |  |  |  | |  |  |  |
| **Random effects** |  |  |  |  |  |  |  | |  |  |  | |  |  |  | |  |  |  |
| σ^2^ | .08 |  |  | . 07 |  |  | . 07 | |  |  |  | |  |  |  | |  |  |  |
| τ₀₀ | .04_ID_ |  |  | .05_ID_ |  |  | . 05_ID_ | |  |  |  | |  |  |  | |  |  |  |
| ICC | .36 |  |  | .39 |  |  | .42 | |  |  |  | |  |  |  | |  |  |  |
| N | 81_ID_ |  |  | 81_ID_ |  |  | 81_ID_ | |  |  |  | |  |  |  | |  |  |  |
| Observations | 161 |  |  | 161 |  |  | 161 | |  |  | 81 | |  |  | 80 | |  |  |  |
| Marginal R^2^ / Conditional R^2^ | .037/.380 |  |  | .053/.419 |  |  | .088 / .469 | |  |  | .016/ -.035 | |  |  | .129/.083 | |  |  |  |

**Note.** A significant main effect of Time and a Group × Time interactions were observed. Post hoc models indicated that non-relapsers showed higher activation at post-treatment (Time 2) compared to relapsers, while no group difference was present at baseline (Time 1). All models controlled for antidepressant medication (ADM), number of past episodes, and residual symptoms.

**Table S29.** Mixed-effects model analysis of mean activation differences in the R. lateral occipital cortex between relapse and non-relapse patients with Major Depression Disorder (MDD).

|  | Group effect | | | Group and Time effect | | | | Group x Time interaction | | | | Group effect at Time 1 | | | | Group effect at Time 2 | | | |
| --- | --- | --- | --- | --- | --- | --- | --- | --- | --- | --- | --- | --- | --- | --- | --- | --- | --- | --- | --- |
| *Predictors* | *Estimates* | *CI* | *p* | *Estimates* | *CI* | *p* | *Estimates* | | *CI* | *p* | *Estimates* | | *CI* | *p* | *Estimates* | | *CI* | *p* |  |
| (Intercept) | -.49 | -.69; -.28 | **<.001** | -.59 | -.80; -.38 | **<.001** | -.67 | | -.91; -.44 | **<.001** | -.66 | | -.91; -.41 | **<.001** | -.30 | | -.52; -.08 | **.009** |  |
| Relapse (yes) | -.18 | -.37; .02 | .074 | -.16 | -.36; .03 | .100 | -.08 | | -.31; .14 | .459 | -.10 | | -.33; .14 | .414 | -.27 | | -.49; -.05 | **.017** |  |
| Time |  |  |  | .19 | .09; .29 | **<.001** | .37 | | .14; .60 | **.002** |  | |  |  |  | |  |  |  |
| ADM (yes) | .11 | -.08; .29 | .246 | .11 | -.08; .29 | .253 | .20 | | -.01; .41 | .061 | .20 | | -.02; .43 | .073 | .02 | | -.19; .22 | .882 |  |
| Episodes | .01 | -.02; .05 | .505 | .01 | -.02; .05 | .473 | .01 | | -.03; .05 | .534 | .01 | | -.03; .05 | .595 | .01 | | -.03; .05 | .571 |  |
| Symptoms | -.04 | -.13; .04 | .348 | -.07 | -.15; .02 | .117 | -.04 | | -.15; .06 | .412 | -.01 | | -.14; .11 | .811 | -.04 | | -.15; .07 | .498 |  |
| Relapse x Time |  |  |  |  |  |  | -.16 | | -.38; .07 | .169 |  | |  |  |  | |  |  |  |
| ADM (yes) x Time |  |  |  |  |  |  | -.19 | | -.40; .02 | .071 |  | |  |  |  | |  |  |  |
| Episodes x Time |  |  |  |  |  |  | -.00 | | -.04 ; .04 | .977 |  | |  |  |  | |  |  |  |
| Symptoms x Time |  |  |  |  |  |  | -.04 | | -.17; .08 | .502 |  | |  |  |  | |  |  |  |
| **Random effects** |  |  |  |  |  |  |  | |  |  |  | |  |  |  | |  |  |  |
| σ^2^ | .12 |  |  | . 11 |  |  | . 10 | |  |  |  | |  |  |  | |  |  |  |
| τ₀₀ | .10_ID_ |  |  | . 11_ID_ |  |  | . 11_ID_ | |  |  |  | |  |  |  | |  |  |  |
| ICC | .44 |  |  | .50 |  |  | .52 | |  |  |  | |  |  |  | |  |  |  |
| N | 81_ID_ |  |  | 81_ID_ |  |  | 81_ID_ | |  |  |  | |  |  |  | |  |  |  |
| Observations | 161 |  |  | 161 |  |  | 161 | |  |  | 81 | |  |  | 80 | |  |  |  |
| Marginal R^2^ / Conditional R^2^ | .053/.469 |  |  | .095/.550 |  |  | .113 / .574 | |  |  | .052/ .003 | |  |  | .102/.054 | |  |  |  |

**Note.** A significant main effect of Time was observed. Yet, no significant Group x Time interaction was observed. All models controlled for antidepressant medication (ADM), number of past episodes, and residual symptoms.

**Table S30.** Cox **proportional hazards models of dynamic treatment neural markers predicting relapse.**

|  | L. Cerebellum | | | L. Supramarginal gyrus | | | R. Precentral gyrus | | | R. Lateral occipital cortex | | |
| --- | --- | --- | --- | --- | --- | --- | --- | --- | --- | --- | --- | --- |
| *Predictors* | *Estimates* | *CI* | *p* | *Estimates* | *CI* | *p* | *Estimates* | *CI* | *p* | *Estimates* | *CI* | *p* |
| ROI change value  (Post - Baseline) | .27 | .12; .61 | **.002** | .25 | .10; .66 | **.005** | .23 | .07 ; .69 | **.009** | .63 | .25; 1.61 | .337 |
| Symptoms (Baseline) | 1.26 | .69; 2.30 | .462 | 1.11 | .65; 1.89 | .707 | 1.12 | .65; 1.92 | .683 | 1.13 | .67; 1.91 | .645 |
| Symptoms (Post-Intervention) | 1.61 | .99 ; 2.61 | .056 | 1.66 | 1.05; 2.63 | **.030** | 1.58 | .98; 2.56 | .061 | 1.56 | .96; 2.53 | .073 |
| ADM | 1.16 | .49; 2.71 | .737 | 1.41 | .59; 3.36 | .436 | 1.00 | .39; 2.61 | .993 | 1.53 | .65; 3.60 | .327 |
| Episodes | 1.08 | .92; 1.26 | .357 | 1.06 | .92; 1.23 | .415 | 1.05 | .90; 1.23 | .539 | 1.00 | .86; 1.16 | .975 |
| Observations | 81 |  |  | 81 |  |  | 81 |  |  | 81 |  |  |
| R^2^ Nagelkerke | .242 |  |  | .202 |  |  | .198 |  |  | .198 |  |  |

**Note.** Cox regression models examined whether change activation in the left occipital cortex, left supramarginal, and right precentral, superior temporal, and inferior frontal gyrus predicted time-to-relapse, controlling for residual symptoms, antidepressant medication (ADM), and past episodes. Higher activations in the left cerebellum, supramarginal, as well as the right precentral gyrus, the superior temporal gyrus was associated with reduced relapse risk

**Table S30.** Cox **proportional hazards models of dynamic treatment neural markers predicting relapse (continued).**

|  | R. Superior temporal gyrus | | | R. Inferior frontal gyrus | | |
| --- | --- | --- | --- | --- | --- | --- |
| *Predictors* | *Estimates* | *CI* | *p* | *Estimates* | *CI* | *p* |
| ROI change value  (Post - Baseline) | .35 | .14; .87 | **.023** | .19 | .06; .63 | **.006** |
| Symptoms (Baseline) | 1.09 | .65; 1.81 | .751 | 1.13 | .69; 1.87 | .626 |
| Symptoms (Post-Intervention) | 1.77 | 1.14; 2.75 | **.011** | 1.57 | .97; 2.54 | .069 |
| ADM | 1.43 | .61; 3.33 | .407 | 1.29 | .55; 3.01 | .561 |
| Episodes | 1.03 | .88; 1.19 | .720 | 1.05 | .91; 1.22 | .501 |
| Observations | 81 |  |  | 81 |  |  |
| R^2^ Nagelkerke | .179 |  |  | .206 |  |  |

**Note.** Cox regression models examined whether change activation in the left occipital cortex, left supramarginal, and right precentral, superior temporal, and inferior frontal gyrus predicted time-to-relapse, controlling for residual symptoms, antidepressant medication (ADM), and past episodes. Higher activations in the left cerebellum, supramarginal, as well as the right precentral gyrus, the superior temporal gyrus was associated with reduced relapse risk

**Table S31.** Combined Cox regression model of relapse risk including static and dynamic neural markers.

| *Predictors* | *Estimates* | *CI* | *p* |
| --- | --- | --- | --- |
| L. Subgenual (Static marker) | 1.32 | .86; 2.03 | .201 |
| R. Occipital cortex (Static marker) | 1.79 | .86; 3.72 | .121 |
| R. Postcentral gyrus (Static marker) | .16 | .05; .52 | **.002** |
| L. Cerebellum (Dynamic marker) | .53 | .21; 1.33 | .176 |
| L. Supramarginal (Dynamic marker) | .12 | .03; .51 | **.004** |
| R. Precentral gyrus (Dynamic marker) | 1.07 | .25; 4.54 | .930 |
| R. Superior temporal gyrus (Dynamic marker) | 1.55 | .57; 4.21 | .385 |
| R. Inferior frontal gyrus (Dynamic marker) | .26 | .05; 1.35 | .109 |
| Symptoms (Baseline) | 1.16 | .59; 2.25 | .671 |
| Symptoms (Post-intervention) | 1.06 | .54; 2.09 | .872 |
| ADM | 1.41 | .49; 4.08 | .530 |
| Episodes | 1.11 | .91; 1.34 | .301 |
| Observations | 81 | | |
| R^2^ Nagelkerke | .473 | | |

**Note.** A multivariate Cox proportional hazards model was estimated to examine relapse risk as a function of both static and dynamic neural biomarkers, while controlling for past depressive episodes, antidepressant medication (ADM) , and residual symptoms at baseline and post-treatment. Static markers included average activation in the **left subgenual cingulate, right lateral occipital cortex,** and **right postcentral gyrus**, whereas dynamic markers reflected change in activation (post − pre) in the **right inferior frontal gyrus, right superior temporal gyrus, right precentral gyrus, left supramarginal gyrus,** and **left cerebellum.**

**Table S32** . **Psychophysiological interaction (PPI) analysis: main effects and predictors of relapse.**

|  |  |  |  |  | Cluster size |  | MNI coordinates (mm) | | |
| --- | --- | --- | --- | --- | --- | --- | --- | --- | --- |
| *Seed region* | *Contrast* | *Region* | *Side* | *p (FDR)* | *k* | *z-score* | *x* | *y* | *z* |
| L. Subgenual cingulate |  |  |  |  |  |  |  |  |  |
|  | *PPI Self > Case* |  |  |  |  |  |  |  |  |
|  |  | Cerebellum | R | .028 | 558 | 3.66 | 32 | -78 | -36 |
|  | *PPI Case > Self* |  |  |  |  |  |  |  |  |
|  |  | Precuneus/ lateral occipital cortex | R | 0 | 2028 | 5.44 | 14 | -70 | 42 |
|  |  | Supramarginal gyrus | R | 0 | 2914 | 4.93 | 56 | -42 | 50 |
|  |  | mPFC | R | .003 | 585 | 5.04 | 44 | 48 | -6 |
|  |  | Supramarginal gyrus | L | .002 | 687 | 4.36 | -58 | -44 | 50 |
|  |  | DLPFC | R | .002 | 663 | 3.97 | 38 | 22 | 28 |
|  | *PPI Relapse > Non-relapse* |  |  |  |  |  |  |  |  |
|  |  | *no regions detected* |  |  |  |  |  |  |  |
|  | *PPI Non-relapse > Relapse* |  |  |  |  |  |  |  |  |
|  |  | *no regions detected* |  |  |  |  |  |  |  |
| R. Lateral occipital cortex |  |  |  |  |  |  |  |  |  |
|  | *PPI Self > Case* |  |  |  |  |  |  |  |  |
|  |  | *no regions detected* |  |  |  |  |  |  |  |
|  | *PPI Case > Self* |  |  |  |  |  |  |  |  |
|  |  | *no regions detected* |  |  |  |  |  |  |  |
|  | *PPI Relapse > Non-relapse* |  |  |  |  |  |  |  |  |
|  |  | *no regions detected* |  |  |  |  |  |  |  |
|  | *PPI Non-relapse > Relapse* |  |  |  |  |  |  |  |  |
|  |  | *no regions detected* |  |  |  |  |  |  |  |
| R. Postcentral gyrus |  |  |  |  |  |  |  |  |  |
|  | *PPI Self > Case* |  |  |  |  |  |  |  |  |
|  |  | Precuneus cortex | L | 0 | 7743 | 5.76 | -14 | -62 | 46 |
|  | *PPI Case > Self* |  |  |  |  |  |  |  |  |
|  |  | *no regions detected* |  |  |  |  |  |  |  |
|  | *PPI Relapse > Non-relapse* |  |  |  |  |  |  |  |  |
|  |  | *no regions detected* |  |  |  |  |  |  |  |
|  | *PPI Non-relapse > Relapse* |  |  |  |  |  |  |  |  |
|  |  | *no regions detected* |  |  |  |  |  |  |  |

**Note.** Whole-brain generalized psychophysiological interaction (gPPI) analyses were conducted using seed regions identified as static relapse markers (left subgenual cingulate, right lateral occipital cortex, and right postcentral gyrus). Seed time series were extracted as the first eigenvariate from each ROI. Connectivity was modeled condition-specific (i.e., self, other, case, and fixation), and the primary contrasts of interest were Self > Case and Case > Self. Results were thresholded at p < .005 (cluster extent k ≥ 400 voxels) and FDR-corrected.

**Figure S1. Study consort diagram.**

**
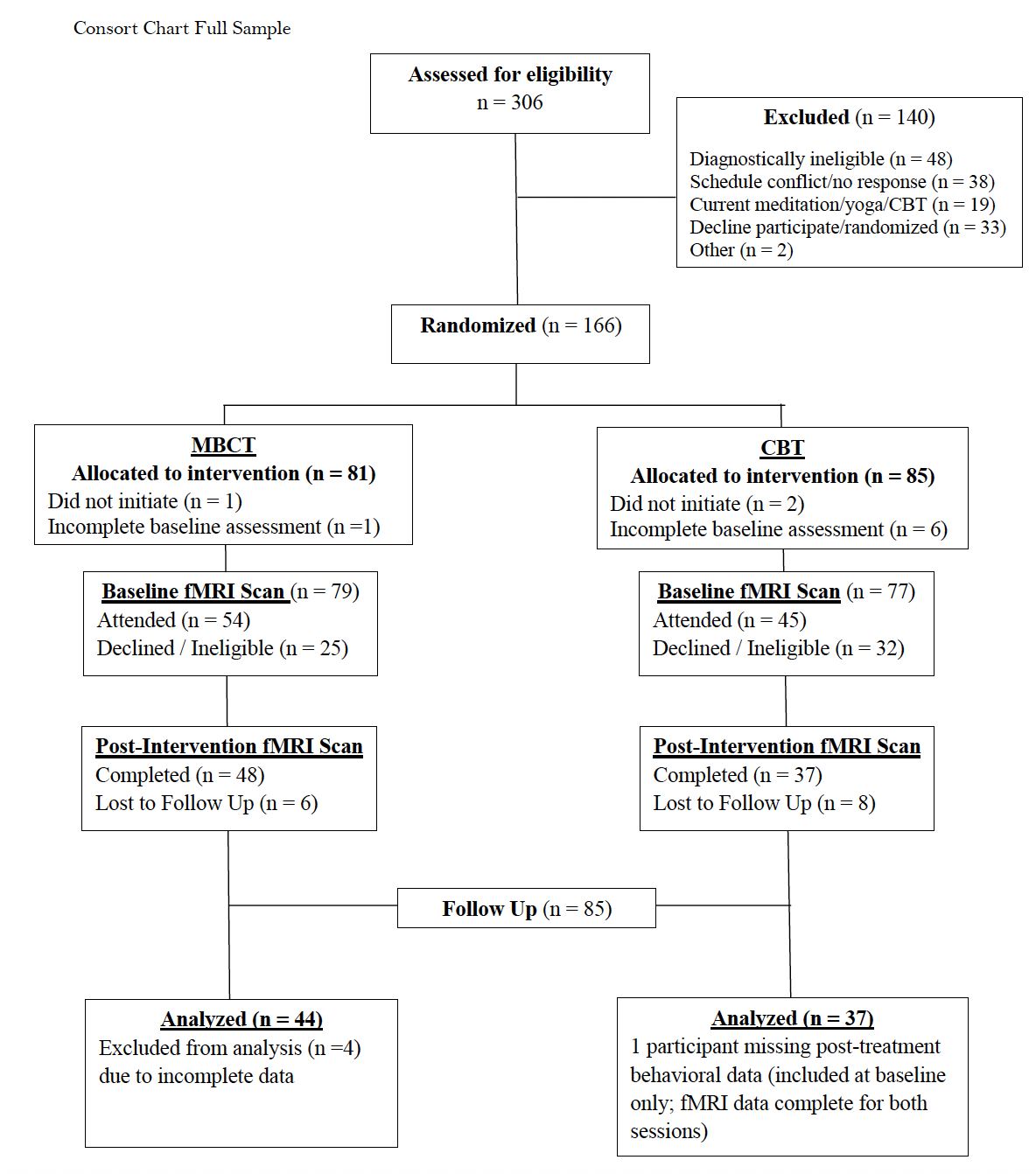
**

**Figure S2. Mixed-effects models of residual symptoms predicting negative self-referential bias.**


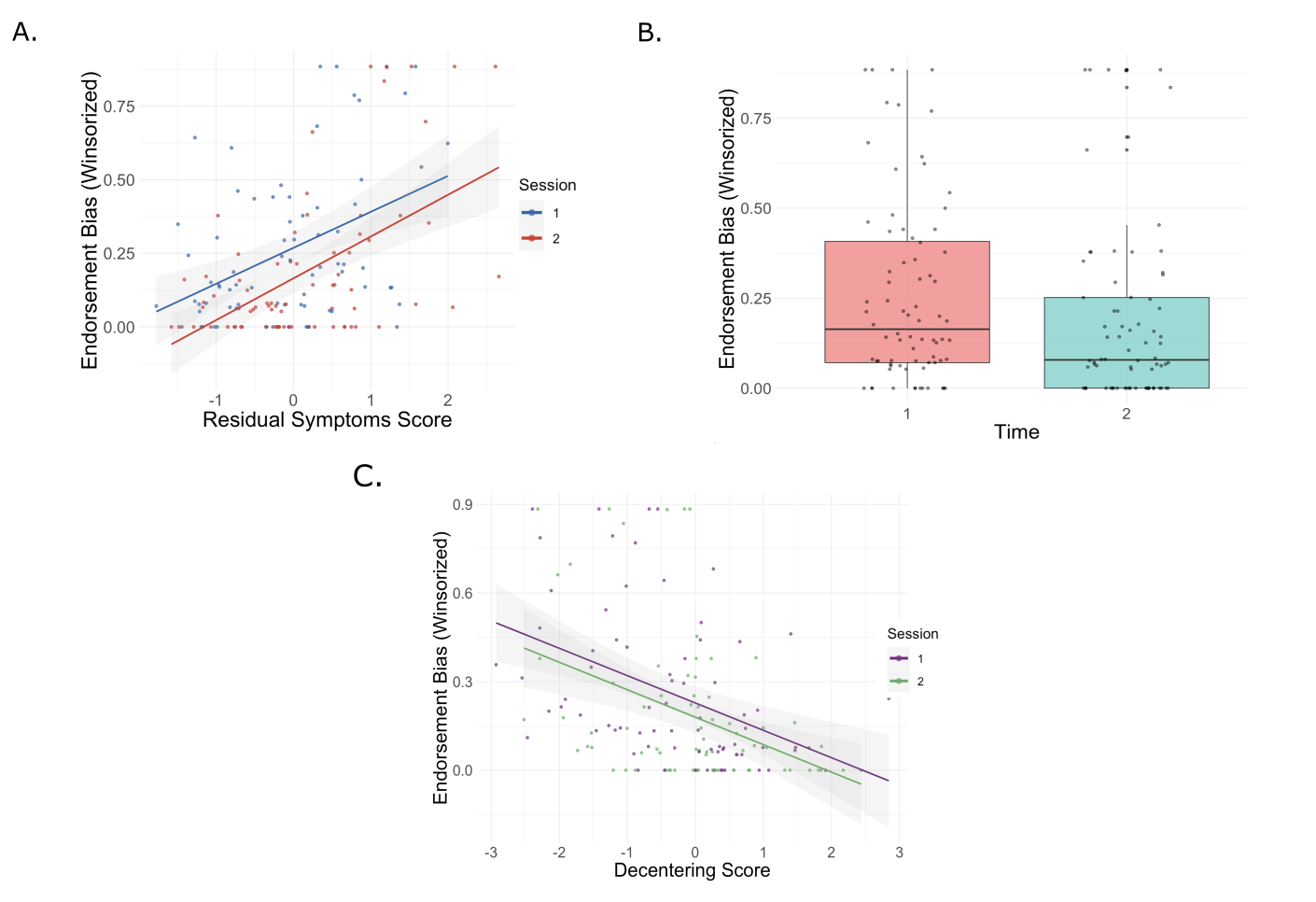


**Note.** Negative self-referential bias (negative/positive endorsements in the self condition) was higher with greater residual symptoms (Panel A) but decreased over time (Panel B). Higher decentering (Panel C) was associated with lower bias

**Figure S3.** ROC curves of baseline brain activation predicting relapse status.


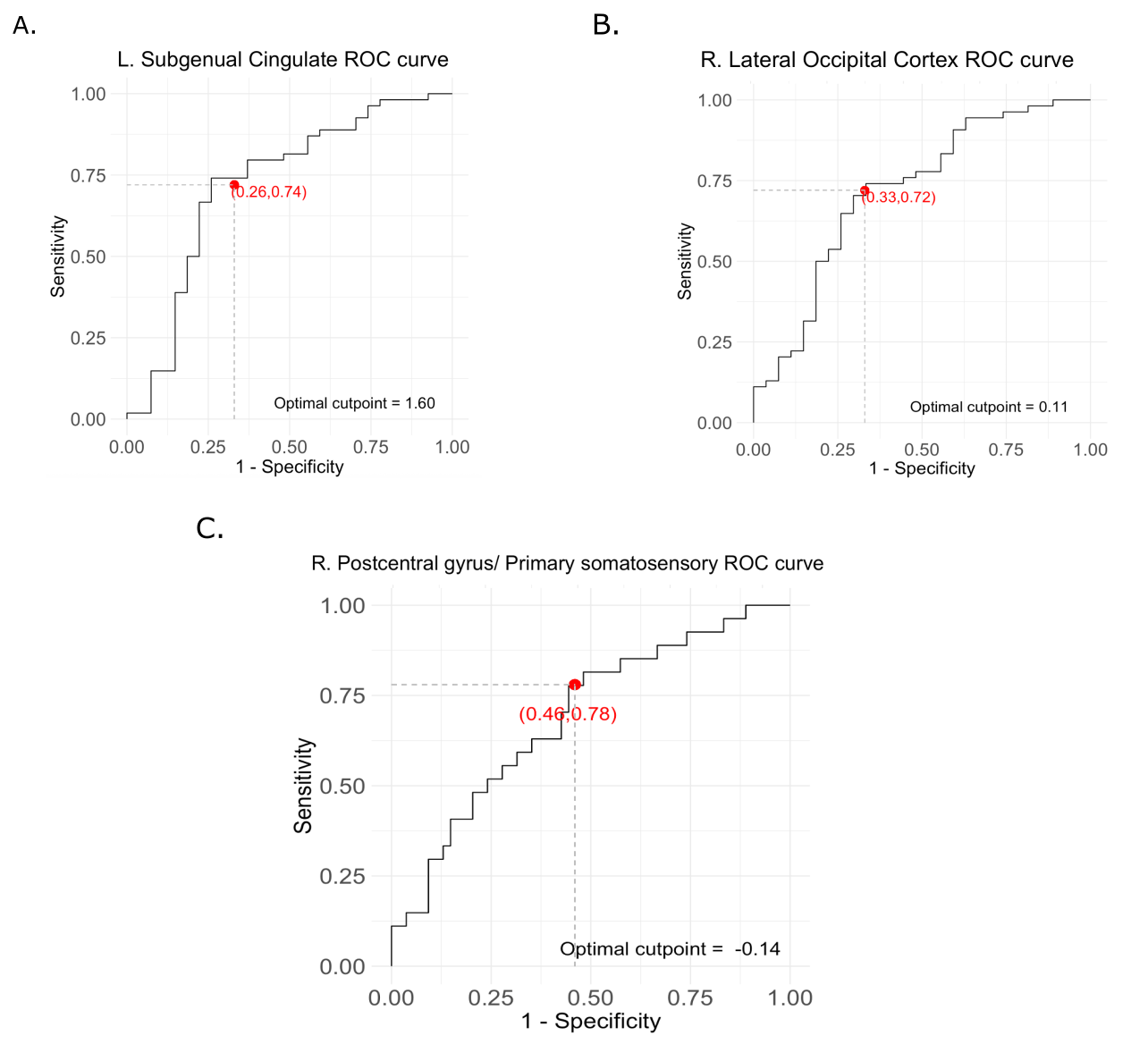


**Note.** Panel A shows the ROC curve for the left subgenual cingulate, with an optimal cutpoint of 1.60. Panel B depicts the ROC curve for the right lateral occipital cortex with an optimal cutpoint of 0.11. Panel C shows the ROC curve for the right postcentral gyrus/ primary somatosensory cortex, with an optimal cutpoint of -0.14

**Figure S4.** ROC curves of dynamic risk markers associated with treatment response.


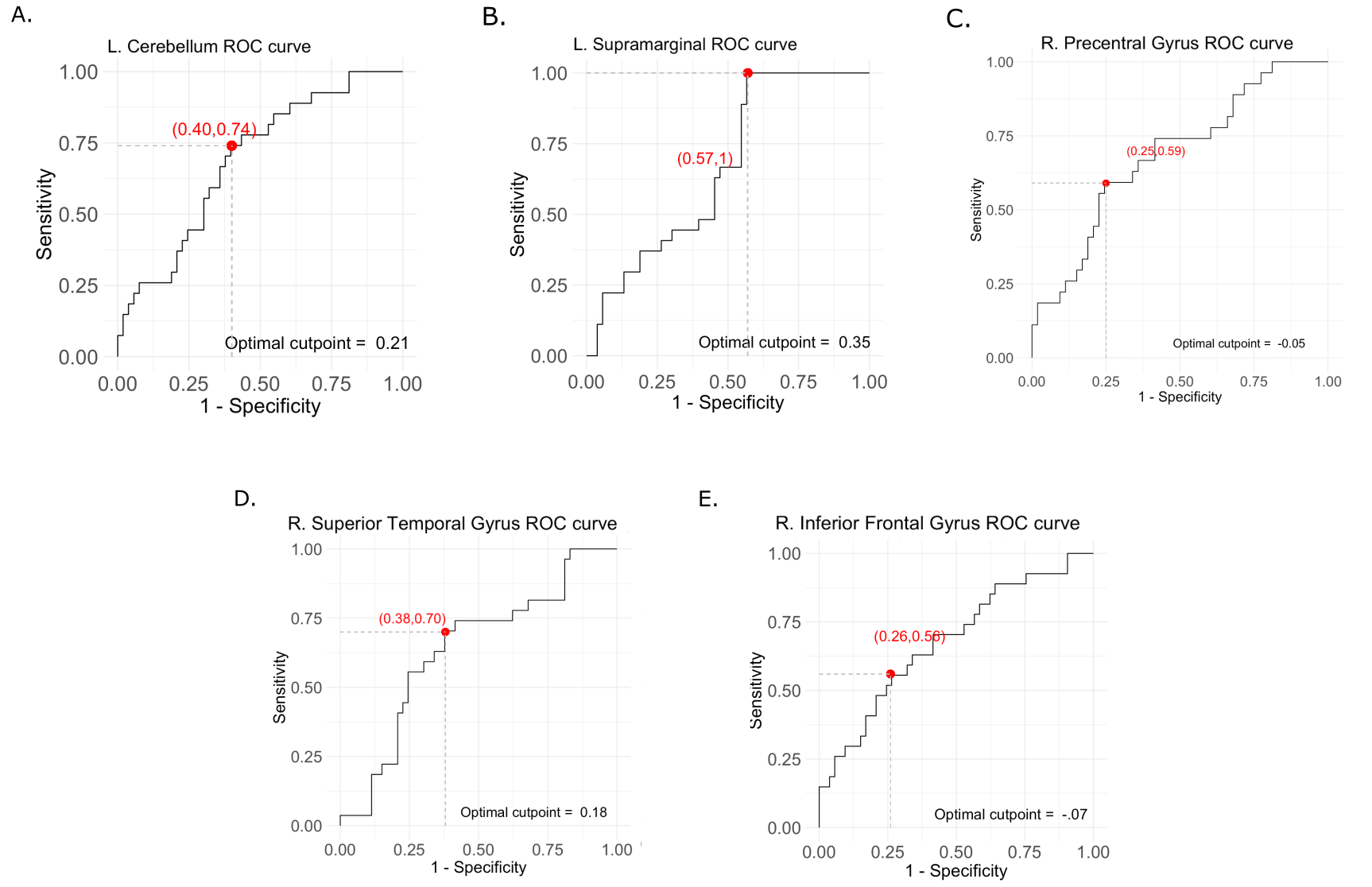


**Note.** Panel A shows the ROC curve for the **left cerebellum**, with an optimal cutpoint of 0.21. Panel B depicts the ROC curve for the **left supramarginal gyrus**, with an optimal cutpoint of 0.35. Panel C shows the ROC curve for the **right precentral gyrus**, with an optimal cutpoint of -0.05. Panel D presents the ROC curve for the **right superior temporal gyrus**, with an optimal cutpoint of 0.18. Panel E shows the ROC curve for the **right inferior frontal gyrus**, with an optimal cutpoint of –0.07


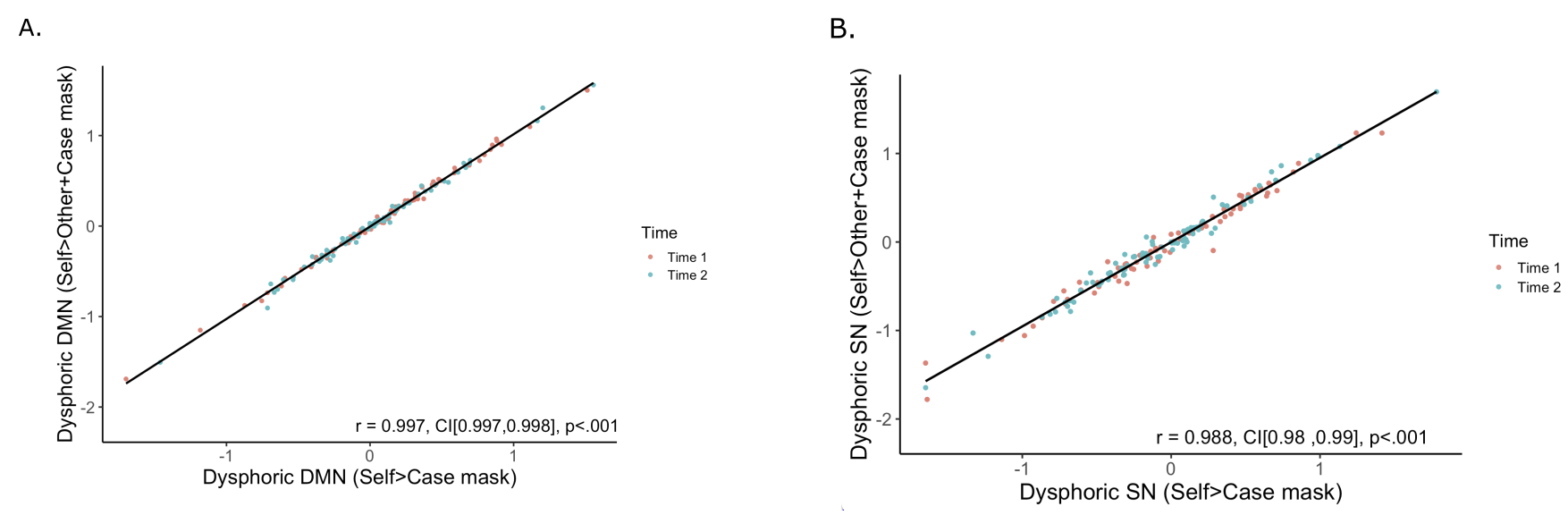
**Figure S5.** Correlation analysis of the frontal default mode network (DMN; superior frontal gyrus and paracingulate gyrus) and salience network (SN; anterior cingulate cortex) dysphoric activity across self-referential contrasts ( Self>Case vs. Self>Other+Case)

**Note.** Dysphoric DMN responses showed high convergence across the two contrast definitions (Panel A). A similarly strong pattern of convergence was observed for dysphoric SN responses (Panel B). Dysphoric self-reference was indexed using the contrast Self_neg > Self_pos constrained within the Self > Case mask, isolating neural responses to self-referential negative versus positive traits. Higher values indicate stronger negative self-referential bias.

**References**

1. Farb N, Anderson A, Ravindran A, Hawley L, Irving J, Mancuso E, et al. Prevention of relapse/recurrence in major depressive disorder with either mindfulness-based cognitive therapy or cognitive therapy. Journal of Consulting and Clinical Psychology. 2018 Feb;86(2):200–4. doi:10.1037/ccp0000266

2. Segal ZV, Anderson AK, Gulamani T, Williams LAD, Desormeau P, Ferguson A, et al. Practice of therapy acquired regulatory skills and depressive relapse/ recurrence prophylaxis following cognitive therapy or mindfulness based cognitive therapy. Journal of Consulting and Clinical Psychology. 2019 Feb 1;87(2):161–70. doi:10.1037/ccp0000351.supp PubMed PMID: 30431297.

3. Farb N. Minding One’s Emotions: Mindfulness Training Alters the Neural Expression of Sadness. Emotion. 2010. doi:10.1037/a0017151.supp

4. Farb NAS, Anderson AK, Bloch RT, Segal ZV. Mood-Linked Responses in Medial Prefrontal Cortex Predict Relapse in Patients with Recurrent Unipolar Depression. Biological Psychiatry. 2011 Aug;70(4):366–72. doi:10.1016/j.biopsych.2011.03.009

5. Farb NAS, Desormeau P, Anderson AK, Segal ZV. Static and treatment-responsive brain biomarkers of depression relapse vulnerability following prophylactic psychotherapy: Evidence from a randomized control trial. Neuroimage Clin. 2022 Feb 19;34:102969. doi:10.1016/j.nicl.2022.102969 PubMed PMID: 35367955; PubMed Central PMCID: PMC8978278.

6. Beck TA, Rush AJ, Shaw BF, Emery G. Cognitive Therapy of Depression. New York, NY, US: Guilford Press; 1979. 442 p.

7. Fava GA, Rafanelli C, Grandi S, Conti S, Belluardo P. Prevention of recurrent depression with cognitive behavioral therapy: preliminary findings. Arch Gen Psychiatry. 1998 Sep;55(9):816–20. doi:10.1001/archpsyc.55.9.816 PubMed PMID: 9736008.

8. Esteban O, Markiewicz CJ, Blair RW, Moodie CA, Isik AI, Erramuzpe A, et al. fMRIPrep: a robust preprocessing pipeline for functional MRI. Nat Methods. 2019 Jan;16(1):111–6. doi:10.1038/s41592-018-0235-4 PubMed PMID: 30532080; PubMed Central PMCID: PMC6319393.

9. Esteban O, Ciric R, Finc K, Blair RW, Markiewicz CJ, Moodie CA, et al. Analysis of task-based functional MRI data preprocessed with fMRIPrep. Nat Protoc. 2020 Jul;15(7):2186–202. doi:10.1038/s41596-020-0327-3

10. Tustison NJ, Avants BB, Cook PA, Zheng Y, Egan A, Yushkevich PA, et al. N4ITK: Improved N3 Bias Correction. IEEE Transactions on Medical Imaging. 2010 Jun;29(6):1310–20. doi:10.1109/TMI.2010.2046908

11. Avants BB, Epstein CL, Grossman M, Gee JC. Symmetric diffeomorphic image registration with cross-correlation: Evaluating automated labeling of elderly and neurodegenerative brain. Medical Image Analysis. 2008 Feb 1;Special Issue on The Third International Workshop on Biomedical Image Registration – WBIR 200612(1):26–41. doi:10.1016/j.media.2007.06.004

12. Zhang Y, Brady M, Smith S. Segmentation of brain MR images through a hidden Markov random field model and the expectation-maximization algorithm. IEEE Trans Med Imaging. 2001 Jan;20(1):45–57. doi:10.1109/42.906424 PubMed PMID: 11293691.

13. Reuter M, Rosas HD, Fischl B. Highly accurate inverse consistent registration: a robust approach. Neuroimage. 2010 Dec;53(4):1181–96. doi:10.1016/j.neuroimage.2010.07.020 PubMed PMID: 20637289; PubMed Central PMCID: PMC2946852.

14. Dale AM, Fischl B, Sereno MI. Cortical surface-based analysis. I. Segmentation and surface reconstruction. Neuroimage. 1999 Feb;9(2):179–94. doi:10.1006/nimg.1998.0395 PubMed PMID: 9931268.

15. Klein A, Ghosh SS, Bao FS, Giard J, Häme Y, Stavsky E, et al. Mindboggling morphometry of human brains. PLoS Comput Biol. 2017 Feb;13(2):e1005350. doi:10.1371/journal.pcbi.1005350 PubMed PMID: 28231282; PubMed Central PMCID: PMC5322885.

16. Fonov V, Evans A, McKinstry R, Almli C, Collins D. Unbiased nonlinear average age-appropriate brain templates from birth to adulthood. NeuroImage. 2009 Jul 1;Organization for Human Brain Mapping 2009 Annual Meeting47:S102. doi:10.1016/S1053-8119(09)70884-5

17. Greve DN, Fischl B. Accurate and robust brain image alignment using boundary-based registration. Neuroimage. 2009 Oct 15;48(1):63–72. doi:10.1016/j.neuroimage.2009.06.060 PubMed PMID: 19573611; PubMed Central PMCID: PMC2733527.

18. Jenkinson M, Bannister P, Brady M, Smith S. Improved Optimization for the Robust and Accurate Linear Registration and Motion Correction of Brain Images. NeuroImage. 2002 Oct 1;17(2):825–41. doi:10.1006/nimg.2002.1132

19. Cox RW, Hyde JS. Software tools for analysis and visualization of fMRI data. NMR Biomed. 1997;10(4–5):171–8. doi:10.1002/(sici)1099-1492(199706/08)10:4/5%3C171::aid-nbm453%3E3.0.co;2-l PubMed PMID: 9430344.

20. Power JD, Mitra A, Laumann TO, Snyder AZ, Schlaggar BL, Petersen SE. Methods to detect, characterize, and remove motion artifact in resting state fMRI. NeuroImage. 2014 Jan 1;84:320–41. doi:10.1016/j.neuroimage.2013.08.048

21. Behzadi Y, Restom K, Liau J, Liu TT. A component based noise correction method (CompCor) for BOLD and perfusion based fMRI. Neuroimage. 2007 Aug 1;37(1):90–101. doi:10.1016/j.neuroimage.2007.04.042 PubMed PMID: 17560126; PubMed Central PMCID: PMC2214855.

22. Satterthwaite TD, Elliott MA, Gerraty RT, Ruparel K, Loughead J, Calkins ME, et al. An improved framework for confound regression and filtering for control of motion artifact in the preprocessing of resting-state functional connectivity data. NeuroImage. 2013 Jan 1;64:240–56. doi:10.1016/j.neuroimage.2012.08.052

23. Lanczos C. Evaluation of Noisy Data. Journal of the Society for Industrial and Applied Mathematics Series B Numerical Analysis. 1964 Jan;1(1):76–85. doi:10.1137/0701007

24. Mikl M, Marecek R, Hlustík P, Pavlicová M, Drastich A, Chlebus P, et al. Effects of spatial smoothing on fMRI group inferences. Magn Reson Imaging. 2008 May;26(4):490–503. doi:10.1016/j.mri.2007.08.006 PubMed PMID: 18060720.
